# Probing the non-covalent forces key to the thermodynamics of β-hairpin unfolding†

**DOI:** 10.1039/d4sc03464c

**Published:** 2024-08-26

**Authors:** Thien H. Tran, Priyanka Prusty, Meghan Ricciardi, Christopher R. Travis, Marcey L. Waters, Bruce C. Gibb

**Affiliations:** a Department of Chemistry, Tulane University School of Science and Engineering New Orleans LA 70118 USA bgibb@tulane.edu; b Department of Chemistry, University of North Carolina at Chapel Hill Chapel Hill NC 27599 USA

## Abstract

Although it is well understood that the graph of the free energy of unfolding (Δ*G*) of a globular protein with temperature approximates to a negative parabola, there is as yet no link between this global (G) Δ*G*_G_(*T*) function and the individual structural elements—residue type and the non-covalent forces between groups—contributing to it. As such, there is little understanding of how each structural element contributes to the globally assessed changes of enthalpy (Δ*H*_G_), entropy (Δ*S*_G_), and heat capacity (Δ*C*_p(G)_) of unfolding calculated from the Δ*G*_G_(*T*) function. To address this situation, we consider here an alternative approach to examining fold stability. Specifically, we examine the local (L) reporting of the thermodynamics of unfolding provided by each residue. By using ^1^H NMR spectroscopy to monitor the response of the individual mainchain amide N–H groups of β-hairpin peptides with temperature, we generate local Δ*G*_L_(*T*) functions, using these to calculate the local enthalpy (Δ*H*_L_), entropy (Δ*S*_L_), and heat capacity (Δ*C*_p(L)_) of unfolding. Mapping the thermodynamic changes in this way, for specific point-mutations, provides new information about how specific residues, non-covalent forces, and secondary structure type, contribute to folding. This type of information provides new details of the factors contributing to the typically measured global Δ*G*_G_(*T*) function.

## Abstract 2

Per-residue analysis of the thermodynamics of unfolding of β-hairpins provides insight into the non-covalent interactions between residues, and the individual contributions of residues and secondary structure type to the Δ*H*, Δ*S*, and Δ*C*_p_ of unfolding.

## Graphical abstract

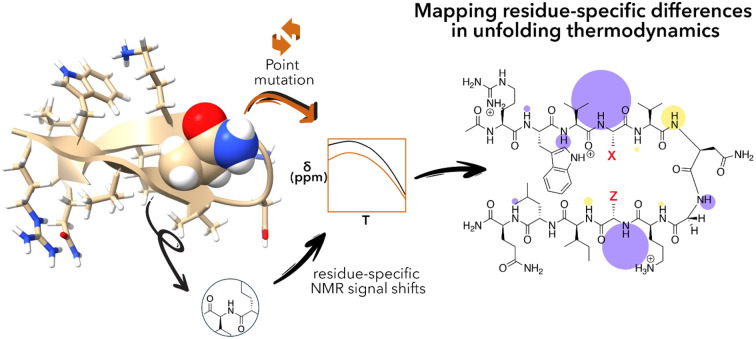

## Introduction

The multi-billion-dollar biologics market^1,2^ is reliant on the stability of the biomacromolecular components during manufacture, storage, and transport. Beyond formulation control with osmolytes and salts, temperature control protocols such as those involving freeze/thaw cycles are key considerations to maintaining activity. This last point is intimately linked to the phenomenon of cold denaturation, whereby folded proteins exhibit a temperature of maximum stability (*T*_max_) from which both increasing and decreasing the temperature leads to unfolding. Whereas heat denaturation of proteins is intuitive, cold denaturation^3–25^ is less so; a lowering of the temperature below *T*_max_ induces the protein to release heat and become more disordered.

Despite substantial effort, there are many open questions surrounding cold denaturation, and the parabolic nature of the free energy of unfolding with temperature (Δ*G*_u_(*T*)) function in general. The Δ*G*_u_(*T*) function is probed using a combination of the two-state model and the Gibbs–Helmholtz equation (eqn (1)) relating it to changes in enthalpy 
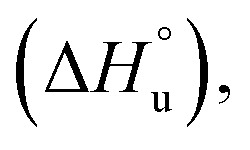
 entropy 
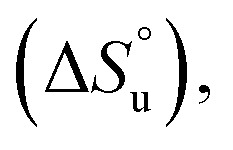
 and heat capacity 
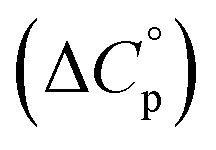
 of unfolding. Here, the positive 
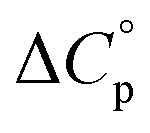
 typically observed with protein unfolding, leads to Δ*G*_u_(*T*) approximating to a negative parabola, with 
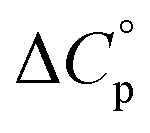
 defining the curvature and 
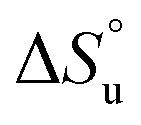
 and 
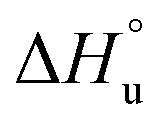
 defining its position on the *x*- and *y*-axes respectively. The maximum of the parabola, *T*_max_, is controlled by 
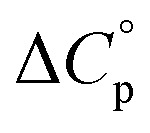
 and 
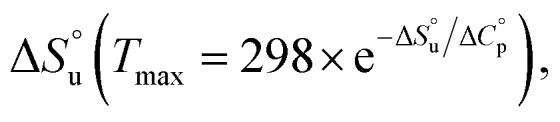
 and it follows from *S* = −d*G*/d*T* that 
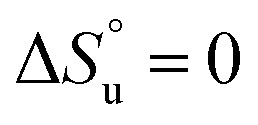
 at *T*_max_, *i.e.*, the (un)folding process at *T*_max_ is entirely driven by enthalpy.^26^1
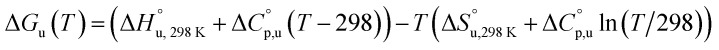


Normally, techniques such as circular dichroism (CD) spectroscopy are used to assess the global (G) stability with temperature: Δ*G*_G_(*T*), and hence determine the globally defined enthalpy (Δ*H*_G_), entropy (Δ*S*_G_), and heat capacity (Δ*C*_p(G)_) of unfolding. However, this approach cannot explain why the Δ*G*_G_(*T*) for metmyoglobin and ribonuclease A are very different;^27^ the *T*_max_ value of the former approaches 40 °C, whereas that of ribonuclease A is well below 0 °C. In other words, there is a disconnect between protein structure, and the nature of the Δ*G*_G_(*T*) parabola. In general terms, it is understood from small molecule studies^10,28–32^ and large proteins^3–10,33^ that a positive 
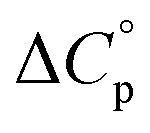
 is characteristic of non-polar groups being solvated upon unfolding, and that 
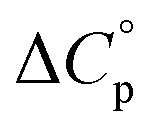
 is proportional to the non-polar surface area.^34^ On the other hand, polar residues are assumed to contribute lower or negative 
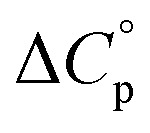
 values. However, beyond these generalities it is unclear how particular residues, secondary-structure type, or the multitude of non-covalent interactions (NCIs) within a folded structure contribute to the Δ*G*_G_(*T*) parabola.^6,13–16,35–39^ This situation is unfortunately made more complicated by the relatively high freezing point of water that—despite workarounds using supercooled solutions^40,41^ reverse micelles,^19,21,42^ organic^43^ or mixed solvents,^44–46^ osmolytes or denaturants,^39,47^ or high pressure,^18,48,49^—can severely limit analysis. New approaches are therefore needed to forge a stronger link between structure and the Δ*G*_G_(*T*) function, and hence improve our understanding of biologics stability and the properties of proteins in general.

As an alternative to the normal approaches, here we investigate the stability of β-hairpin peptides using each individual mainchain amide N–Hs as a reporter. Thus, we utilize ^1^H NMR spectroscopy to map the local (L) Δ*G*_L_(*T*) responses of each amide signal, and so obtain the local Δ*H*_L_, Δ*S*_L_, Δ*C*_p(L)_ and *T*_max(L)_ value as reported by each residue. Thus, our approach uses the two-state model to treat each residue as a unique, uncoupled system fully independent of the other residues. This approach is of course incongruous with the fact that the non-covalent interactions between residues dictate that they do not act independently, but are coupled. Nevertheless, as we discuss this per-residue analysis does reveal new details of the individual contributions from each, the non-covalent interactions between them, as well as trends in the thermodynamic parameters that shed light on how β-sheet and β-turns contribute to (un)folding and hence the form of Δ*G*_G_(*T*) parabolae. As such, this approach amounts to a first step towards a new model relating individual residue contributions to global protein stability.

## Results

**Fig. 1:**
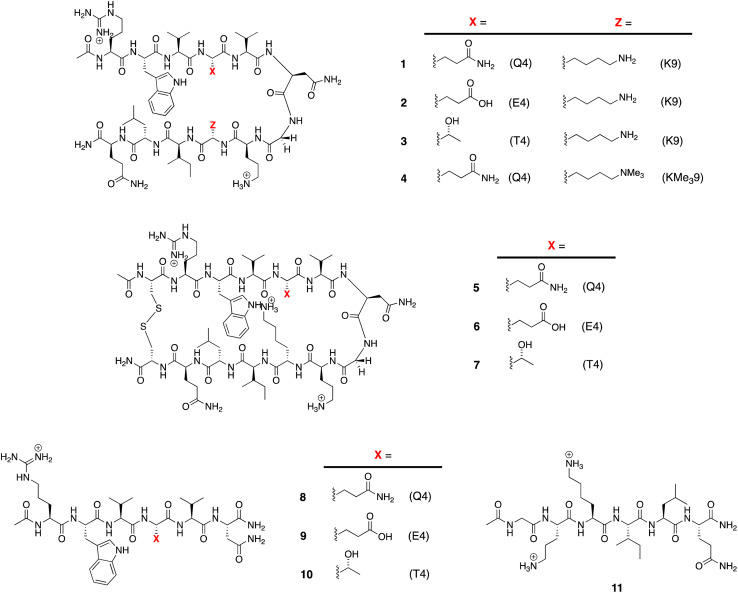
Peptides used in this study.

The structures of the β-hairpins used are shown in Fig. 1 (see Section 1 of the ESI for synthetic details†). Their general design originated in the Gellman group,^50–52^ but included two modifications from the Waters' lab;^37,53–55^ specifically, a tyrosine to tryptophan (Y2W) mutation to give an enhanced W2⋯Z9 cation–π interaction, and a proline to asparagine (P6N) mutation to give a _5_VNGO_8_ (O = ornithine, Orn) type 1′ turn.^56,57^ Thus, these β-hairpins are designed to be held together by a core formed of a cation–π interaction between W2 and X9 (Lys-9 (K9) or trimethyl Lys-9 (KMe_3_9)), and non-polar interactions between W2 and leucine-11 (L11), and valine-3 (V3), V5, and isoleucine-10 (I10).

Following literature precedent, ^1^H NMR spectroscopy was first used to determine the extent of global folding by measuring the signal separation of the C_α_H protons of glycine-7 (G7).^37,58^ By comparison to macrocycles 5–7 as surrogates for the fully-folded state, and half-peptides 8–11 as surrogates for the unfolded state, G7 can be used as a global reporter of peptide-folding *via*eqn (2):

2 Fraction folded (*f*) = [*δ*_obs_ − *δ*_0_]/[*δ*_100_ − *δ*_0_]

where *δ*_obs_ is the observed G7 signal split of the C_α_H protons (in ppm) of the hairpin, and *δ*_100_ and *δ*_0_ are respectively the signal differences in the corresponding (100% folded) macrocyclic surrogate and (0% folded) 6-mer peptides. By this approach, β-hairpins 1–4 were determined to be respectively 52%, 77% 79% and 78% folded at room temperature (Section 4, ESI†). Thus, each of the three, single-point mutations upon peptide 1, to give 2, 3, or 4, increased the folding percentage by ∼26%. Fraction folded values (*f*) can be readily converted to the global free energy of unfolding Δ*G*_G_ using eqn (3). These values are given in Table 1.^59^

3 Δ*G*_G_ = −*RT* ln((1 − *f*)/*f*)

**Table 1:** Room temperature (298 K), global (G) thermodynamic parameters of unfolding of peptides 1–4 as reported by G7^a^^,^^b^

Thermodynamic parameters	1	2	3	4
Δ*G*_G_ (kJ mol^−1^)	0.20 (0.02)	3.04 (0.52)	3.28 (0.36)	3.14 (0.41)
Δ*H*_G_ (kJ mol^−1^)	12.76 (0.51)	12.95 (1.83)	20.88 (0.29)	13.64 (1.78)
Δ*S*_G_ (J mol^−1^ K^−1^)	42.47 (1.73)	33.03 (6.16)	59.55 (0.97)	35.03 (6.01)
Δ*C*_p(L)_ (J mol^−1^ K^−1^)	554.3 (43.8)	677.4 (156.4)	414.8 (24.6)	543.6 (27.1)
*T*_max(G)_ (K)	276 (2)	284 (4)	258 (2)	279 (3)

a Errors are shown in parenthesis, and are determined by: error propagation using eqn (1) and (2) (Δ*G*_G_), and fitting to eqn (3) (Δ*H*_G_, Δ*S*_G_, and Δ*C*_p(L)_).

b The data for peptide 2 and 4 was similar to that obtained previously by the same approach.^38,53,55^

To determine the remaining thermodynamic parameters, we monitored the G7 C_α_H protons signal-splitting from 10 to 60 °C. This data (Section 4, ESI†) was then fitted to the Gibbs–Helmholtz equation (eqn (1)) to yield the complete, thermodynamic profile of unfolding (Table 1).^60^ This global analysis revealed similar endothermicities for unfolding 1, 2 and 4, but a much larger endothermicity of unfolding of 3. Additionally, each unfolding process was entropically promoted and involved positive Δ*C*_p(L)_ values attributed to the hydration of non-polar groups upon unfolding.^10^

The majority of work with hairpins involve pH values ∼ 7, where amide N–H exchange rates are close to the NMR timescale and their signals not always observable. Consequently, to observe each mainchain amide N–H signal we carried out all studies at pH = 2.3.^61^ Full experimental details are provided in the ESI.†

**Fig. 2:**
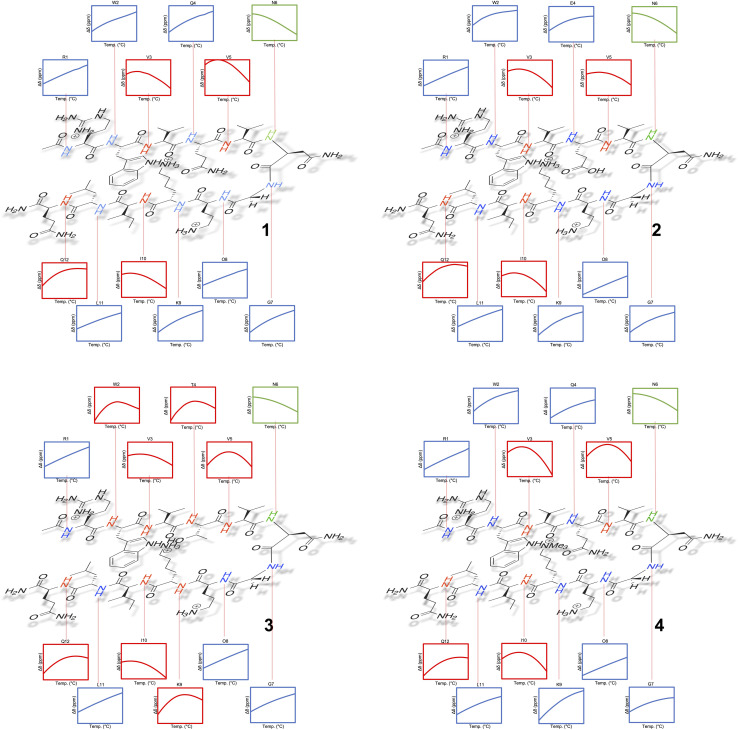
Temperature dependent ^1^H NMR signal shifts (*δ*) for each individual mainchain amide N–H group of peptide 1–4. For each box of data, the height of the *y*-axis is 0.3 ppm (normalized scale, *δ* at 298 *K* = 0), and the width along the *x*-axis is 10–60 °C (283–333 K). Shown functions are the average obtained from the triplication of data.

Tracking the mainchain N–H amide signals of each peptide as a function of temperature (*δ*(*T*)) resulted in twelve curves for each peptide, which were classified in three ways (Fig. 2): data that is approximately linear with a positive gradient (blue), data that is approximately linear and possessing a negative gradient (green), and data this is distinctly non-monotonic (red). Although this is *δ*(*T*) data rather than Δ*G*_L_(*T*) data, several points are worthy of note. First, the blue functions arises largely from outward-pointing amide groups. The two exceptions are the N-terminal R1 amide that is least likely to be involved in inter-strand hydrogen bonding, and the O8 amide involved in a weak, non-linear hydrogen bond because of its tight proximity to the turn and high solvation. Due to a lack of curvature, this data suggests a low Δ*C*_p(L)_ and a high *T*_max(L)_. Second, the N6 signal function (green) suggests a low Δ*C*_p(L)_ and a low *T*_max(L)_. Asn-6 is generally considered to be an unusual residue because of its extreme down-field position and its sensitivity to folding/unfolding; hence the negative gradient. Third, the non-monotonic functions (red) are all from strongly hydrogen bonded amides intimately associated with the cation–π–hydrophobe core of the peptide. These have observable (or close to observable) *δ*_max_ values, and suggest relatively high Δ*C*_p(L)_ values. As we discuss below, these three assessments on Δ*C*_p(L)_ residue values large hold when the thermodynamic data is calculated.

Separately, a comparison of the response of each residue in the four peptides demonstrates that the Q4T mutation (1 → 3) is intrinsically different from the other mutations. Thus, the *δ*(*T*) response of the X4 residue in 1 and 3 are very different, whereas the differences in the responses of X4 in 1 and 2 are quite similar (and the difference in the response of X9 in 1 and 4 essentially the same).^62^ Moreover, relative to the other mutations, the T4 residue in 3 induces non-monotonicity in the data of two additional residues: outward pointing W2 and K9.

**Fig. 3:**
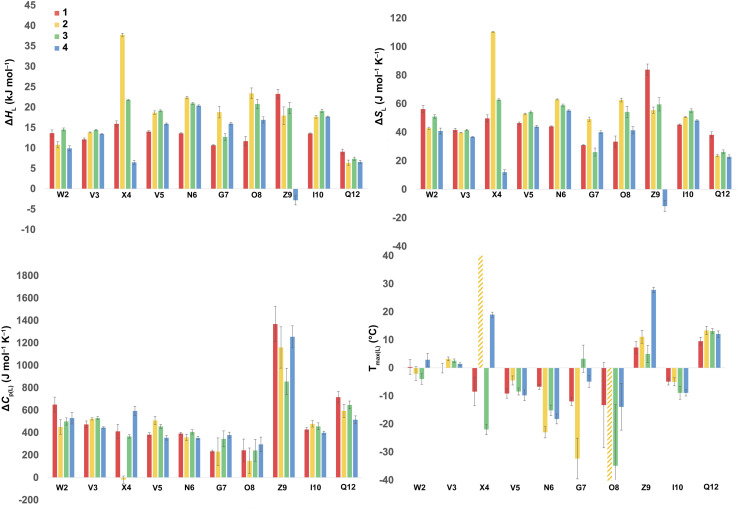
Δ*H*_L_, Δ*S*_L_, and Δ*C*_p(L)_ of unfolding and *T*_max(L)_ values as reported by each residue of peptides 1–4. Values are derived from the ^1^H NMR signal shift, for each individual mainchain amide N–H group, as a function of temperature (using eqn (1)–(3)). Because of signal overlap between each peptide and its reference 6-mer half peptide, data could not be obtained for R1 and L11. Extreme changes in *T*_max(L)_ for residues X4 (very high) and O8 (−77 °C) in 2 are cut off and highlighted with hatched color. Shown error bars are fitting errors.

Using the corresponding reference peptides and eqn (1) and (3), we determined the local (L) Δ*G*_L_(*T*) curves and calculated the thermodynamics of unfolding reported by each residue.^63^Fig. 3 shows this Δ*H*_L_ Δ*S*_L_ and Δ*C*_p(L)_ data for the reporting residues, as well as their calculated *T*_max(L)_ values. *En masse*, the Δ*H*_L_ and Δ*S*_L_ data is complex, but the Δ*C*_p(L)_ data does reveal that the smallest heat capacity changes are reported by the turn residues _6_NGO_8_, consistent with their relatively high degree of solvation in the folded state. The Δ*C*_p(L)_ data also reveals remarkably high values for Z9. As (similarly-cationic) O8 does not show such extreme values, we assume this is intrinsic to the W2⋯Z9 cation–π interaction. Between these extremes are values reported by Q12, W2, and the non-polar residues in the core. Separately, the *T*_max(L)_ data reveals that turn residues have generally lower values than those toward the termini.

**Fig. 4:**
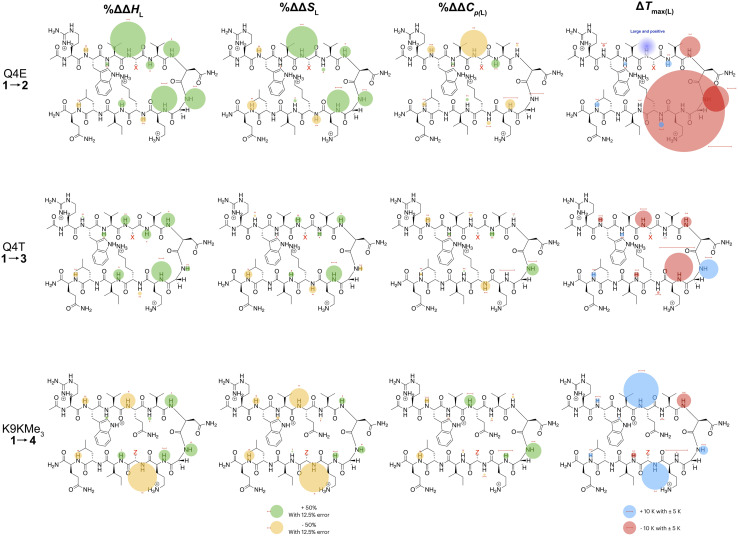
Per-residue reporting of the % ΔΔ*H*_L_, % ΔΔ*S*_L_ and % ΔΔ*C*_p(L)_ and Δ*T*_max(L)_ for unfolding of the mutations: top row, Q4E (1 → 2); middle row, Q4T (1 → 3), and; bottom row, K9KMe_3_ (1 → 4). Because of signal overlap between each peptide and its reference 6-mer half peptide, data could not be obtained for R1 and L11. For the Δ*H*_L_, Δ*S*_L_ and Δ*C*_p(L)_ data, changes are shown as percentage increases (green) or decreases (yellow) proportional to the area of the bubble. For Δ*T*_max(L)_, changes are shown in kelvin (K). All values are indicated, though bubbles for insignificant changes may not be apparent. Scaling bubbles and error bars for the two types of bubbles are shown at the foot of the figure. Specific values of Δ*H*_L_, Δ*S*_L_ and Δ*C*_p(L)_ as reported by each amide N–H are given in Fig. 3.

In the following discussion, we will refer to the data shown in Fig. 3 periodically. However, analyzing this data by considering the changes upon each point-mutation of 1 provides more insight, and we represent the per-residue differences between the two peptides using the ‘bubble maps’ shown in Fig. 4. Each row in Fig. 4 shows the percentage change in Δ*H*_L_, Δ*S*_L_, and Δ*C*_p(L)_, as well as the calculated *T*_max(L)_ changes reported by each residue, for the mutations: Q4E (1 → 2), Q4T (1 → 3), and K9KMe_3_ (1 → 4).

In the Q4E mutation, the enthalpy map demonstrates the increased folding percentage (globally, 52 to 77% folded) is largely driven by the turn residues, where all residues report an increased endothermicity of unfolding. In contrast, despite the lack of data from R1 and L11, the enthalpic contributions from the terminal “half” of the peptide are small. The largest change is observed for X4. As Fig. 3 shows, the E4 residue of 2 is quite distinctive in the magnitude of the reported endothermicity of unfolding. Modeling suggests that the E4 carboxylic acid carbonyl can readily form a hydrogen bond to the G7 amide N–H (Section 2.3, ESI†); an extra non-covalent interaction supported by the downfield shift of the proton upon the Q4E mutation. We assume this additional non-covalent interaction is a major contributor to the enhanced stability of 2. In contrast, conspicuous in its absence upon the Q4E mutation is a sizeable change in the enthalpy change reported by K9. This suggests that the mutation has little effect on any X4⋯K9 ion–dipole interaction (–NH^3+^⋯O=C), or despite the low pH, the presence of a salt-bridge in peptide 2. Indeed, the only observable NOEs involving the sidechain methylenes of E4 are with the amides of V5, G7, and O8.^64^ Taken together, this data is consistent with a E4⋯G7 hydrogen bond in 2 that augments the extent of global folding.

The entropy bubble map reveals that, without exception, all residues report enthalpy–entropy compensation (see Section 5.2 of ESI†). In other words, the tighter fold of the Q4E mutation results in the majority of the increased ordering of the system occurring at the turn.

Examining the heat capacity map reveals a significant change at the mutation site. The specific Δ*C*_p(L)_ values for X4 of 1 and 2, are +412 and −19 J mol^−1^ K^−1^ (Fig. 3) confirm a surprising large change in the reported Δ*C*_p(L)_ upon the minor change of replacing the –C(O)NH_2_ of 1 with a –C(O)OH in 2. As there is no evidence of a meaningful E4⋯K9 interaction, we tentatively attribute the low Δ*C*_p(L)_ value of E4 in 2 to its hydrogen bond to the amide of G7. However, solvation changes may also play a role here.

**Fig. 5:**
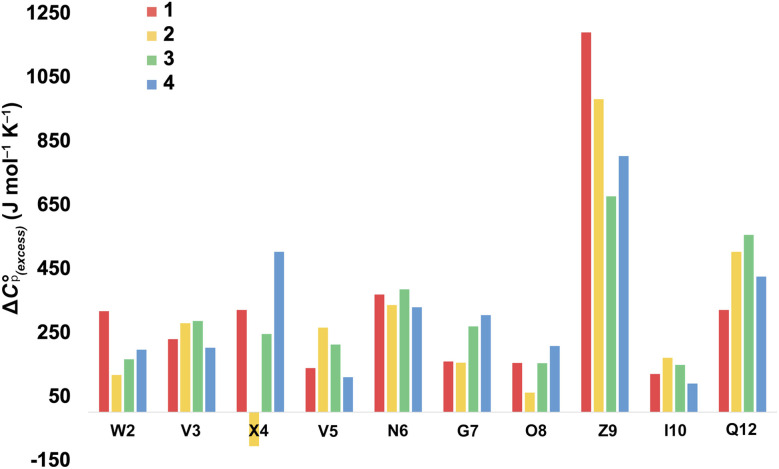
Excess heat capacity Δ*C*_p(excess)_ reported by each residue. The shown values are differences between Privalov's calculated heat capacity change of hydration of amino-acid sidechains (*Δ*^w^_g_*C*_p_) and the Δ*C*_p(L)_ reported in Fig. 3.

For more information concerning the heat capacity differences between X4 of 1 and 2, we compared the Δ*C*_p(L)_ values reported by each residue of each peptide (Fig. 3) to the partial molar heat capacity change of hydration of the amino acid side-chains (*Δ*^w^_g_*C*_p_) calculated by Privalov.^65^ Subtraction of the *Δ*^w^_g_*C*_p_ values from the data given in Fig. 3 reveals the excess heat capacity Δ*C*_p(excess)_ within each residue in each folded peptide.^66^Fig. 5 shows this data. Focusing on the data for X4, whereas the Q4 residue of 1 reports a positive Δ*C*_p(excess)_ suggesting that this residue is poorly solvated in the folded state, the E4 residue of 2 reports a negative Δ*C*_p(excess)_. Since *Δ*^w^_g_*C*_p_ corresponds to the fully solvated side chain, we attribute this negative Δ*C*_p(excess)_ to the hydrogen bonding between E4 with G7 acting as a strong heat sink that is lost upon the unfolding of 2.

Other significant changes in Δ*C*_p(L)_ from the Q4E mutation (Fig. 4) occur at W2, V5, and O8. The increase at V5 and decrease at O8 may result from hydration changes induced respectively by the sidechain methylenes and terminal carboxylic acid of E4. In contrast, W2 is remote from E4, and so this change may simply reflect a subtle repacking of the core.

The Δ*T*_max(L)_ values for the Q4E mutation are shown in Fig. 4. Because E4 in 2 reports a very small Δ*C*_p(L)_ and a relatively large error, and because of the corresponding large increase in Δ*S*_L_, the *T*_max_ reported by E4 is unreliable. However, the large increases in *T*_max(L)_ for turn residues _6_NGO_8_ are significant, and are all rooted in the increased order (Δ*S*_L_) of the turn. The value for O8 is especially large because in addition to the change in order, there is also an increase in local hydration (Δ*C*_p(L)_) upon hydrogen bonding of E4 to neighboring G7.^67^

For the Q4T mutation (1 → 3), the enthalpy map also demonstrates that the increased folding percentage (globally, 52 to 79% folded) is driven by the turn. These enthalpy changes are generally smaller than in the Q4E mutation. In the energy-minimized structure of 3 the T4 OH group hydrogen bonds to the V3 carbonyl, whilst the T4 methyl fills the small concavity created by the turn residues (see Section 2.4 of ESI†) In contrast, in the structure of 2, the E4 carboxylic acid hydrogen bonds to the G7 amide (Section 2.3, ESI†). The enthalpy bubble maps for the Q4E and Q4T mutations suggest that the E4⋯G7 hydrogen bond contributes more to folding than does the T4 sidechain interactions.

As with the Q4E mutation, the enthalpic contributions from the terminal “half” of the peptide are relatively small. Furthermore, the enthalpy and entropy bubble maps reveal that all residues—barring G7—report enthalpy–entropy compensation (see Section 5.2 of the ESI†).

Examining the Δ*C*_p(L)_ map for Q4T reveals only weak patterning. However, as with the Q4E mutation the inner-core amides of V3, V5, and I10 all report small increases in Δ*C*_p(L)_, suggesting that these protons are more isolated from water in 2 and 3 than they are in 1. The excess heat capacity values reported by these residues (Fig. 5) also support this notion. However, the biggest changes in heat capacity for the Q4T mutation are observed at G7 (47% increase from 234 to 344 J mol^−1^ K^−1^) and K9 (37% decrease from 1369 to 858 J mol^−1^ K^−1^).^68^ In hydrogen bonding to the V3 carbonyl, the T4 OH group is located at the end of the groove between the indole of W2 and the sidechain of K9 (3.0 Å from the K9 C_β_ methylene). And as just discussed, the T4 methyl rests in the concavity of the turn (see Section 2.4 of ESI†). Thus, we attribute the increase reported by G7 to a degree of shielding provided by the proximal (3.2 Å) T4 methyl, and the decrease reported by K9 to the hydration of the W2–K9 groove by the OH group. Heat capacity changes elsewhere are minor. Thus, as with the Q4E mutation, the Q4T mutation is not felt by the _1_RW_2_ and _10_ILQ_12_ sections of the hairpin.

The largest decreases in *T*_max(L)_ for the Q4T mutation (Fig. 4) are for X4, N6, and O8, which are all rooted in the increased structure (order) in more folded 3, whilst the large increase in *T*_max(L)_ for G7 is rooted in both its large increase in Δ*C*_p(L)_ and its decrease in the entropy of unfolding.

A similar analysis (Fig. 4) can be carried out for the K9KMe_3_ mutation (1 → 4), which increases (global) folding from 52 to 78%. From the enthalpy map it is apparent that half the residues report increases in the endothermicity of unfolding, and half decreases. The largest decreases are reported by W2, Q4, and Z9. For Z9, the cation–π interaction switches from one that enthalpically disfavors unfolding in 1 (Δ*H*_L_ = 23.3 kJ mol^−1^), to one that enthalpically promotes unfolding in 4 (Δ*H*_L_ = −2.8 kJ mol^−1^). These values are in contrast to the global values reported by G7 (Table 1). The other large reductions in Δ*H*_L_ reported by W2 and Q4, suggesting a non-covalent network in 4 involving W2⋯Z9⋯Q4. This was not apparent in 1–3, but presumably the large size of KMe_3_ is key here. Counteracting these sizable destabilizations are four turn residues that, as is the case with the other mutations, all report enhanced endothermicities of unfolding.

The entropy data for the K9KMe_3_ mutation again reveals good enthalpy–entropy compensation, with the largest change in Δ*S*_L_ reported by Z9. Thus, although the cation–π interaction becomes energetically repulsive in folded 4, this is more than compensated for by this interaction being promoted by entropy. As reported in Table 1, global analysis suggests the entropic benefit for unfolding 4 is smaller than that of 1, but this hides the actual entropic benefits of forming the W2⋯KMe_3_9 interaction.

The corresponding heat capacity data for K9KMe_3_ shows that the most significant differences are at Q4 and G7. The change in absolute value for Δ*C*_p(L)_ reported by Z9 (Fig. 3) is nullified by a large error, but despite this there is evidently a ‘knock-on’ effect at Q4, and in turn, G7. For each residue the increase in Δ*C*_p(L)_ is indicative of a less polar environment for the turn residues, which we interpret as the large and non-polar headgroup of KMe_3_9 of 4 crowding neighboring Q4 and reducing the hydration of the turn.

The largest Δ*T*_max(L)_ values for the K9KMe_3_ mutation are reported by Q4, N6, and Z9 (Fig. 4). The positive change at Q4 is the result of order (Δ*S*_L_) and solvation (Δ*C*_p(L)_) changes, whilst the positive change at Z9 is rooted in entropy; as is the decrease in *T*_max(L)_ for N6. Thus, the favorable entropy of the W2⋯KMe_3_ interaction dominates the large *T*_max(L)_ increases reported by Q4 and K9.

## Discussion

Considering the data collectively, we envision two important questions. First, what do these local reported thermodynamic values tell us about the physical forces at play within β-hairpins? And second, how does local data relate to the global data typically measured? Regarding the first question, local reporting evidently provides thermodynamic information that ties in with structural information from NMR and computation. We envision that ultimately we will be able to identify what can be called, thermodynamic signatures; singular or multifactorial thermodynamic measures that are characteristic of structural elements such as residue types/functional groups, secondary structure type, or NCIs between residues. The work here has focused on just four peptides, and it is obvious that a much larger set of data is required before a comprehensive list of definitive thermodynamic signatures can be formulated. Nevertheless, the data in hand does suggest some specific thermodynamic signatures, and points to others that will need further studies to confirm.

Comparing all three mutations, an increase in folding percentage is rooted mostly in the enthalpy and entropy changes in the turn region _4_XVNGO_8_. In terms of enthalpy, the largest changes in the turn are seen in the Q4E mutation (1 → 2). Smaller enthalpic enhancements are seen for the Q4T mutation (1 → 3) and the K9KMe_3_ mutation (1 → 4); though they are smaller and the changes in the latter are masked somewhat by the change in the thermodynamics of the cation–π interaction reported by W2 and X9. The details of these differences are rooted in the different ways that each mutation enhances stability: the E4 acid group of 2 can hydrogen bond to the G7 amide; the T4 sidechain of 3 is involved in hydrogen bonding to the V3 carbonyl and packing the concavity of the turn, whilst the K9KMe_3_ mutation changes the nature of the cation⋯π interaction to one that is enthalpically repulsive but entropically attractive. Thus, the K9KMe_3_ mutation leads to a reported exothermicity of unfolding; the opposite recorded by synthetic hosts^69,70^ designed to recognize KMe_3_ residues in histone proteins.^38,53,55,71–73^

From the limited set of four peptides, the introduction of a carboxylic acid (Q4E) or trimethylammonium (K9KMe_3_) group induces large changes in the enthalpy and entropy data reported by the mutation site itself; the corresponding effect of the Q4T mutation is small. Moreover, observed enthalpy/entropy changes in neighboring residues are more apparent with the Q4E or K9KMe_3_ mutations. However, more information is needed to determine if, for example, the E4 residue of 2 affects the neighboring turn residues directly or indirectly *via* solvent mediation. Similarly, the enthalpy/entropy data for the single K9KMe_3_ hints at an X4⋯Z9 interaction in 4, but other examples are needed to explore such a cross-strand interaction. In summary, enthalpy changes for the mutations studied are most evident at the turn residues. There are specifics still to explore here, but generally the observed enthalpy–entropy compensation means that increased stability is reflected in greater ordering of the turn residues.

In terms of the heat capacity changes, the effect of each mutation is generally focused on the turn residues, although the Q4E and K9KMe_3_ mutations also affect the X4 (*i* − 1) site. We ascribe this to local changes in solvation brought about by mutation. However, further mutants are required to understand the subtilties of the patterning of positive and negative changes in the values reported by the different residues. This stated, the Δ*C*_p(excess)_ values reported in Fig. 5 reveal that the majority of residues report a positive excess heat capacity. The most striking observation here is the large, positive Δ*C*_p(excess)_ reported by X9. Each cation–π interaction of Z9 residues reports as ‘extremely hydrophobic’, with Δ*C*_p(excess)_ values demonstrating that unfolding leads to a very large increase in the heat storage capabilities of an ammonium group as water molecules replace the indole ring of W2 upon unfolding. We currently therefore view this high Δ*C*_p(L)_ of the cation–π interaction as a (singular) thermodynamic signature of this familiar NCI. In contrast, neighboring O8 is well solvated in the folded state and there is little Δ*C*_p(excess)_ in this residue. These contrasting properties clearly demonstrate that context is key; the local environment of a positive charge dictates how it contributes to the thermodynamics of unfolding.

On the other hand, the hydrogen bonding of E4 in folded 2 appears itself to be a heat-reservoir, and unfolding leads to a distinct loss of heat capacity. This loss is evidently larger than any loss due to solvation changes, resulting in a negative Δ*C*_p(excess)_. However, more examples of peptides containing glutamic and aspartic acid residues are required to solidify or modify this concept.

In general, we attribute the Δ*C*_p(excess)_ demonstrated by all residues to the NCIs between them; including those between the mainchain atoms. Nevertheless, there are many open questions regarding the data in Fig. 5. For example, what is the cause of the relatively large positive Δ*C*_p(excess)_ values for N6 and Q12? Are the values reported by N6 rooted in the tightness of the turn? Modeling suggests that the relatively large positive Δ*C*_p(excess)_ value reported by Q12 is in part rooted to the hydrogen bonding between its sidechain and the sidechain of R1, and its packing to adjacent V3 and I10 (see Section 2.5 of the ESI†), but more is needed here to confirm this. Also, is the increase in Δ*C*_p(excess)_ values for G7 in folded 3 and 4 rooted in a decreased solvation of the residue induced by respectively the methyl T4 in 3 and the KMe_3_ group in 4?

Studies of cold denaturation are facilitated by high *T*_max_ values, and the entropy and heat capacity data obtained here allows us to come to some intriguing conclusions that we have not noted to have been previously articulated. First, because turn residues are more solvent exposed, their changes in heat capacity upon unfolding are generally small. This inevitably contributes to lowering *T*_max(L)_ values. Moreover, when mutation increases the extent of folding, there is an inevitable increase in order in the turn residues. This also tends to lower *T*_max(L)_ values. Thus, in general turn residues have intrinsically lower *T*_max(L)_ values relative to those in sheet structure, and enhanced folding exacerbates this difference. Finally, from the limited set of data thus far gathered a key design principal that can counter the low intrinsic *T*_max(L)_ values for turn residues is to introduce hydrophobicity near to or at the turn. These lead to less well solvated turn residues that display larger Δ*C*_p(excess)_ values. Regardless, the combination of entropy and heat capacity changes at a turn means that a thermodynamic signature of this type of secondary structure are low intrinsic *T*_max(L)_ values.

Returning to the second question of how local data relates to the global data typically measured, simply averaging the local (L) values give global averages that are reasonable, and sometimes very close to the value obtained by the global (G) assessment using the G7 methylene splitting (see Table 1 and Section 4.4 of the ESI†). However, we believe that a better understanding of the relationship between local and global thermodynamic data requires more peptides and statistical analysis.

## Conclusions

We have examined the unfolding of β-hairpin peptides using each mainchain amide as a local reporter. This provides a wealth of thermodynamic data that can be linked to structural elements such as the NCIs that occur between residues. Thus, the four peptides discussed here suggest that a thermodynamic signature of the cation–π interaction is an extremely high Δ*C*_p(excess)_ of unfolding. Our work here also suggests that hydrogen bonding carboxylic acids may be heat reservoirs. Finally, the results described here demonstrate that turn residues have intrinsically low *T*_max(L)_ values, which presumably contribute to reducing the globally assessed *T*_max(G)_ value of a folded peptide. This suggests that proteins with fewer turns will tend to exhibit higher *T*_max(G)_ values. We are carrying out further studies to reveal more about how specific residues, secondary structure type, and NCI contribute to fold stability and the phenomenon of cold denaturation. We will report these findings in due course.

## Data availability

The data that support the findings of this study are available in the ESI† of the article.

## Author contributions

THT performed the complete analysis of peptides 1, 3 and 4. PP carried out the complete analysis of peptide 2. MR and CRT performed the synthesis of peptides 1–7. MLW oversaw the peptide synthesis and provided insight into β-hairpin design. BCG designed the overall study and advised THT and PP.

## Conflicts of interest

There are no conflicts to declare.

## Supplementary Material

SC-OLF-D4SC03464C-s001
