# Supplementary material for: Probing the non-covalent forces key to the thermodynamics of β-hairpin unfolding
Source: Chem Sci. 2024 Aug 26;15(38):15588–98. doi: 10.1039/d4sc03464c (PMC11369966; doi:10.1039/d4sc03464c)
Supplement: SC-015-D4SC03464C-s001 [file SC-015-D4SC03464C-s001.pdf]

Supplementary Information (SI) for Chemical Science.
This journal is © The Royal Society of Chemistry 2024

Supporting Information

Probing the non-covalent forces key to the thermodynamics of β-hairpin unfolding

Thien H. Tran,[1] Priyanka Prusty,[1] Meghan Ricciardi,[2] Christopher R. Travis,[2] Marcey L. Waters,[2] Bruce C. Gibb[1]

[1] Department of Chemistry
Tulane University School of Science and Engineering
New Orleans, LA 70118, USA

[2] Department of Chemistry
University of North Carolina at Chapel Hill
Chapel Hill, NC 27599, USA

Thien H. Tran orcid.org/0009-0001-9359-5829
Priyanka Prusty orcid.org/0009-0008-2706-6428
Meghan Ricciardi orcid.org/0000-0003-3249-2241
Christopher R. Travis orcid.org/0000-0003-0673-6978
Marcey L. Waters orcid.org/0000-0002-4917-5755
Bruce C. Gibb orcid.org/0000-0002-4478-4084

TABLE OF CONTENTS

Acronyms and abbreviations..S4
1. Materials, instrumentation, and sample preparation S5
1.1. Peptide synthesis and purification..S5
1.1.1. Peptide sequences...S5
1.1.2. General peptide synthesis protocol S6
1.1.3. Peptide workup and cleavage protocol S6
1.1.4. Cyclization of fully folded β-hairpin controls S7
1.1.5. LCMS Data of β-Hairpin peptides S7
1.2. Other materials and instrumentation S7
1.3. NMR sample preparation S7
2. Peptide characterization S8
2.1. NMR assignments..S8
2.2. Quantifying the extent of folding..S12
2.3. Structure prediction by AlphaFold and energy minimization S13
2.4. Orientation of the 4th residue side chain...S26
2.5. Side chain packing between R1 and Q12 ....S28
3. VT NMR experiments..S30
3.1. VT NMR data of peptide 1...S31
3.2. VT NMR data of peptide 2...S32
3.3. VT NMR data of peptide 3...S34
3.4. VT NMR data of peptide 4...S35
3.5. VT NMR data of peptide 5...S36
3.7. VT NMR data of peptide 7...S38
3.8. VT NMR data of peptide 8...S39
3.9. VT NMR data of peptide 9...S40
3.10. VT NMR data of peptide 10...S41
3.11. VT NMR data of peptide 11 ....S42
4. Data fitting..S43
4.1. Error analysis S43
4.2. Thermodynamic data of unfolding by glycine splitting..S43
4.3. Residue-wise thermodynamics fitting..S46
4.3.1. Visualization of VT NMR data...S46
4.3.2. Methodology..S50
4.3.3. Residue-wise thermodynamics data S51
4.4. Comparison between local fitting and global fitting S55
5. Data visualizations S56

5.1. Unfolding of 1-4 .... S56
5.2. Enthalpy-entropy compensation of 1-4 .... S59
5.3. Enthalpy-entropy compensation of single mutations S62
6. Side chain non-monotonicity S65
References S67
Appendix I S68

Acronyms and abbreviations

Ac	Acetyl
ACN	Acetonitrile
DCM	Dichloromethane
DIC	*N,N'*-diisopropycarbodiimide
DMF	*N,N*-dimethylformamide
Eq	Equivalent
Fmoc	Fluorenylmethoxycarbonyl
HPLC	High performance liquid chromatography
HTML	Hyper-Text Markup Language
LCMS	Liquid chromatography – mass spectroscopy
NMR	Nuclear magnetic resonance
O (in a peptide sequence)	Orn – ornithine
Oxyma	Ethyl cyanohydroxyiminoacetate
R (in equations)	Ideal gas constant, R = 8.314 J/K·mol
T	Temperature
TFA	Trifluoroacetic acid

1. Materials, instrumentation, and sample preparation

1.1. Peptide synthesis and purification

1.1.1. Peptide sequences

Three β-hairpin peptides **1**, **2**, **3** and **4** were chosen for this study based on previous report from Waters group (Figure S1).[1] As references, corresponding cyclic peptides of **5**, **6** and **7**, as well as 6-mer 'half-peptides' **5**, **6** and **7** were also synthesized to quantify the degree of folding of **1**, **2**, **3** and **4**. Note that **8** and **11** were used for the case of **4** since *N*-methylation of lysine does not induce significant change in chemical shifts as shown in the previous study.[1] Table S1 summarizes the primary sequences of the peptides studied.

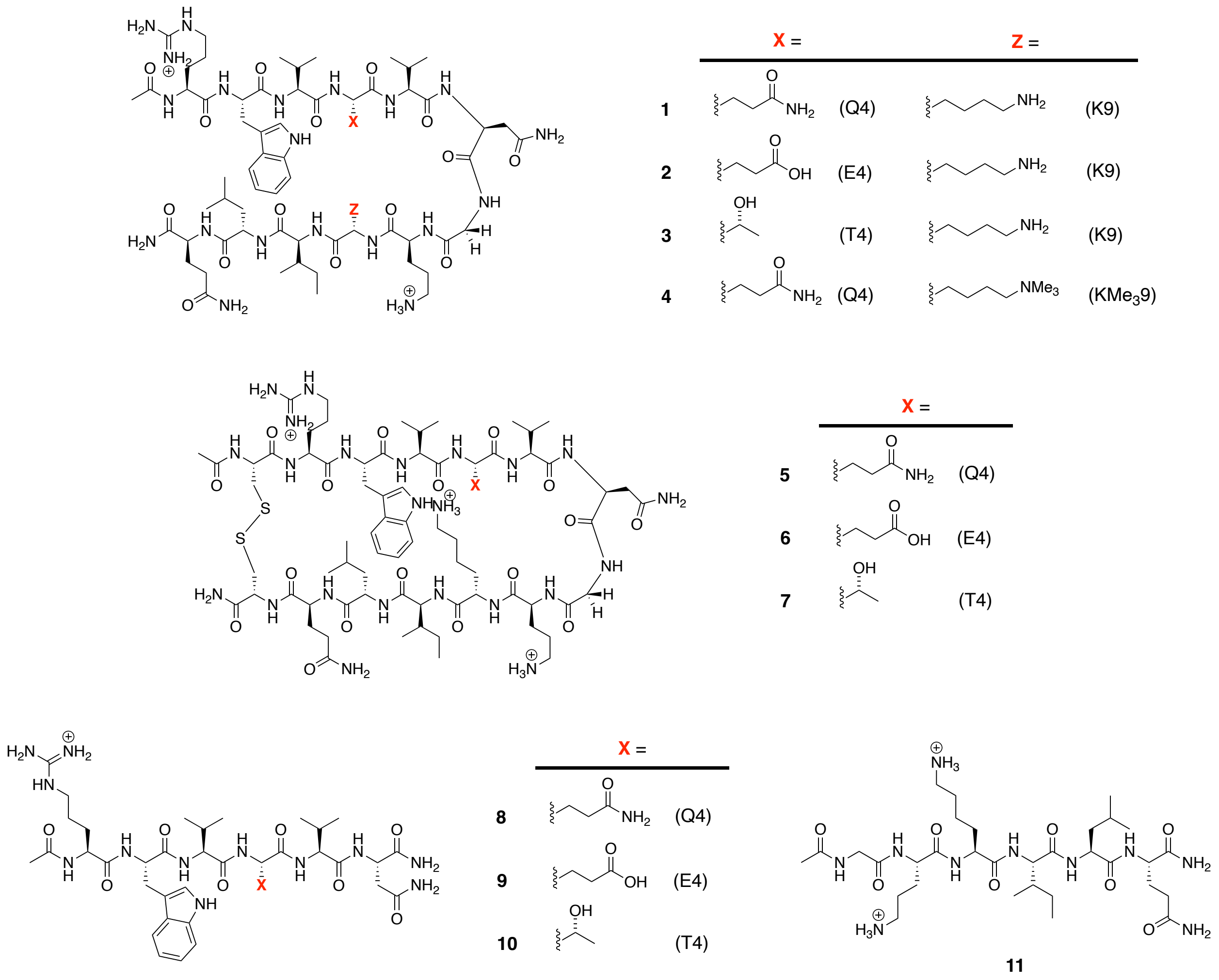

Figure S1. *Peptides' primary structures in the study.*

Table S1. *One-letter code sequence of each peptide in the study*

Peptide	Sequence
**1**	Ac-RWVQVNGOKILQ-NH_2
**2**	Ac-RWVEVNGOKILQ-NH_2
**3**	Ac-RWVTVNGOKILQ-NH_2
**4**	Ac-RWVQVNGO(KMe_3)ILQ-NH_2
**5**	Ac-CRWVQVNGOKILQC-NH_2
**6**	Ac-CRWVEVNGOKILQC-NH_2
**7**	Ac-CRWVTVNGOKILQC-NH_2
**8**	Ac-RWVQVN-NH_2
**9**	Ac-RWVEVN-NH_2
**10**	Ac-RWVTVN-NH_2
**11**	Ac-GOKILQ-NH_2

1.1.2. General peptide synthesis protocol

All peptides (except **3**, **9** and **10**, which were purchased from GenScript and characterized by mass spectroscopy analysis) were synthesized utilizing a CEM Liberty Blue Peptide Synthesizer. Standard Oxyma-DIC couplings were conducted with Fmoc protected amino acids with Rink Amide AM resin. 5 eq of each canonical amino acid (4 eq of non-canonical amino acids) was coupled using 5 eq of Oxyma and 10 eq of DIC during two coupling cycles at 90 °C for 4 minutes (10 minutes for Arg) in DMF. A solution of 20% piperidine in DMF was bubbled through the resin for two cycles each 1 minute long, washing the resin with DMF between each cycle.

Synthesis of unnatural amino acid Fmoc-LysMe$_3$-OH

This amino acid was synthesized via previously described protocol utilizing a reductive amination reaction followed by an S_N2 reaction.[2] 2.0 g of Fmoc-Lysine-OH (5 mmol) was dissolved in 3:1 MeOH (300 mL) to ACN (100 mL). To this mixture, 10 eq of sodium triacetoxyborohydride (STAB) (50 mmol) and 10 eq of formaldehyde was added and allowed to stir at room temperature for 16 hours. After 16 hours, an additional 10 eq of both STAB and formaldehyde was added to the mixture and the reaction was left to stir an additional 16 hours. 10 eq of iodomethane (50 mmol) and 10 eq of $KHCO_3$ was then added and the mixture was stirred for an additional 16 hours. The reaction was monitored via LCMS and when completed was dried down and purified via reverse phase column chromatography. A gradient of 0-100% B was used with solvent A comprising of 95:5 water: ACN with 0.1% TFA and solvent B is 95:5 ACN:water with 0.1% TFA. Product purity was confirmed via LCMS with pure product being concentrated and lyophilized.

1.1.3. Peptide workup and cleavage protocol

All peptides had the final Fmoc deprotection occur in a peptide flask with subsequent *N*-terminal acetylation. Each β-hairpin was transferred to a peptide flask and bubbled with a 20% piperidine in DMF solution for 15 minutes each, twice, washing with DMF between cycles. After the deprotection, 6% 2,5-lutidine and 5% acetic anhydride in DMF was added and bubbled through the resin for 30 minutes. The resin was then washed 3 times with DMF, followed by DCM and MeOH alternating 3 times ending on DCM. The peptide was then cleaved using 95%: 2.5%: 2.5% TFA:triisopropylsilane:water bubbling for 3 hours. Peptides are then purified via reverse phase column chromatography using a gradient of 0-1 % B with solvent A comprising of 95:5 water:ACN with 0.1% TFA and solvent B is 95:5 ACN:water with 0.1% TFA. Additional purification was conducted using reverse phase HPLC with a gradient of 0-70% B in 60 minutes with solvent

A containing 95:5 water: ACN with 0.1% TFA and solvent B having 95:5 ACN: water with 0.1% TFA. Pure peptides were concentrated and lyophilized to dryness, with purity being confirmed via LCMS.

1.1.4. Cyclization of fully folded β-hairpin controls

Cyclic peptides **5, 6** and **7** were formed via disulfide bond formation of cystine residues at *C* and *N* terminus of the peptides following previous protocols.[3] After cleavage of the cyclic peptides from the resin and reverse phase column purification, the lyophilized peptides were then dissolved in 10 mM phosphate buffer at pH 7.4 with 1% DMSO and left overnight at room temperature. Upon reaction completion (confirmed via LCMS), the peptides were then purified via reverse phase HPLC as described above.

1.1.5. LCMS Data of β-Hairpin peptides

The main LCMS peaks and their expected mass are summarized in the table below.

Table S2. *Expected and observed mass of the peptides from LCMS.*

Peptide	Expected Mass (amu)	Observed Mass (amu)
**1**	1495.80	1494.76
**2**	1498.88	1496.10
**3**	1468.75	1468.40
**4**	1538.89	1537.22
**5**	1700.06	1698.89
**6**	1702.89	1700.23
**7**	1673.03	1671.56
**8**	841.97	841.41
**9**	842.95	842.50
**10**	814.94	814.90
**11**	712.89	712.52

1.2. Other materials and instrumentation

All solutions were prepared in ultra-pure water (resistivity of 18.2 MΩ·cm at 25 °C). All peptide structures were characterized by collecting nuclear magnetic resonance (NMR) spectra on a Bruker 700 MHz spectrometer. All variable temperature (VT) experiments were performed on a Bruker 700 MHz. All data were analyzed by either MATLAB 2022a or Microsoft Excel.

Variable temperature NMR spectra were collected for **1-11** from the range 10-60 °C on a Bruker 700 MHz with cryoprobe. The spectra were collected using 8-16 scans at each temperature, after the probe was equilibrated for at least 10 minutes. The probe temperature was calibrated using methanol and ethylene glycol standards.

1.3. NMR sample preparation

The peptide concentration was fixed at 5 mM, except for the three peptides **8, 9** and **10** which were studied at 1 mM due to their low solubilities. Unless otherwise noted, all solutions were 90% H_2O:10% D_2O buffered to pH 2.3 with 50 mM H_3PO_4.

2. Peptide characterization

2.1. NMR assignments

Chemical shift (in ppm) values of all proton signals are reported below (Table S3-S13).

Table S3.** Chemical shift values (δ, ppm) for peptide **1

Residue/ Group	Amide N–H	H_α	H_β	H_γ	H_δ	H_ε	Side chain N–H
N-Acetyl		1.82					
R1	8.00	4.19	1.56	1.47, 1.39	3.01		7.13, 7.00
W2	8.18	4.86	3.02	7.13, 7.31	7.39	7.31	10.06
V3	8.51	4.20	1.92	0.77			
Q4	8.36	4.62	1.93, 1.83	2.17			7.34, 6.69
V5	8.55	4.07	1.89	0.81			
N6	9.02	4.46	2.89, 2.67				7.50, 6.83
G7	8.47	3.96, 3.69					
O8	7.87	4.43	1.80	1.71, 1.62	2.93		7.52
K9	8.35	4.52	1.60	1.19	1.33	2.60	7.24
I10	8.72	4.32	1.75	1.34, 1.08	0.79		
L11	8.25	4.06	1.32, 0.92	1.13	0.50, 0.37		
Q12	8.45	4.20	1.96, 1.80	2.19			7.30, 6.76
C-Amide							7.55, 7.01

Table S4.** Chemical shift values (δ, ppm) for peptide **2

Residue/ Group	Amide N–H	H_α	H_β	H_γ	H_δ	H_ε	Side chain N–H
N-Acetyl		1.82					
R1	7.97	4.26	1.56	1.53, 1.39	3.03		7.02
W2	8.22	4.98	2.98	7.13, 6.96	7.22	7.39	10.07
V3	8.89	4.33	1.92	0.76			
E4	8.43	4.80	1.90, 1.82	2.19			
V5	8.79	4.10	1.83	0.81			
N6	9.30	4.37	2.96, 2.66				7.51, 6.83
G7	8.50	3.97, 3.62					
O8	7.74	4.50	1.73	1.72, 1.62	2.90		7.53
K9	8.39	4.60	1.57	1.24	1.11	2.46	7.14
I10	9.00	4.44	1.77	1.31, 1.08	0.78		
L11	8.23	3.96	1.25, 0.95	0.66	0.40, 0.19		
Q12	8.55	4.20	1.92, 1.77	2.16			7.23, 6.76
C-Amide							7.57, 7.00

__Table S5.__ Chemical shift values (δ, ppm) for peptide __3__

Residue/ Group	Amide N–H	H_α	H_β	H_γ	H_δ	H_ε	Side chain N–H
N-Acetyl		1.82					
R1	7.96	4.30	1.61	1.45	3.07		7.01
W2	8.25	4.99	3.00				10.07
V3	8.92	4.49	2.00	0.78			
T4	8.39	4.84	3.92	0.94			
V5	8.78	4.11	1.85	0.84			
N6	9.33	4.38	2.94, 2.67				7.54, 6.84
G7	8.38	4.01, 3.54					
O8	7.78	4.46	1.76	1.62	2.93		7.55
K9	8.45	4.72	1.59	1.19	1.27	2.51	7.14
I10	9.09	4.52	1.78	1.34, 1.07	0.77		
L11	8.20	3.94	1.22, 0.61	0.91	0.38, 0.17		
Q12	8.56	4.22	1.96, 1.79	2.17			7.24, 6.76
C-Amide							7.58, 7.01

__Table S6.__ Chemical shift values (δ, ppm) for peptide __4__

Residue/ Group	Amide N–H	H_α	H_β	H_γ	H_δ	H_ε	Side chain N–H
N-Acetyl		1.96					
R1	7.96	4.27	1.58	1.53, 1.40	3.04		6.96
W2	8.21	5.02	2.99	7.13, 6.96	7.24	7.40	10.13
V3	8.96	4.35	1.95	0.76			
Q4	8.40	4.98	1.87	2.07			7.34, 6.61
V5	8.73	4.12	1.84	0.81			
N6	9.31	4.36	2.96, 2.66				7.51, 6.84
G7	8.56	4.01, 3.60					
O8	7.77	4.51	1.74	1.71, 1.61	2.94		7.53
K(Me_3)9	8.40	4.89	1.65	1.21	1.34	2.44	Me: 2.46
I10	9.04	4.49	1.78	1.32, 1.08	0.79		
L11	8.22	4.04	1.29, 0.97	0.77	0.37, 0.13		
Q12	8.58	4.23	1.94, 1.76	2.16			7.29, 6.76
C-Amide							7.58, 7.01

Table S7. *Chemical shift values (δ, ppm) for peptide* ***5***

Residue/ Group	Amide N–H	H_α	H_β	H_γ	H_δ	H_ε	Side chain N–H
N-Acetyl		1.96					
C0	8.27	5.11	2.91, 2.31				
R1	8.62	4.51	1.72	1.59, 1.41	3.08		7.01
W2	8.57	5.04	2.99, 2.84	7.13, 6.99	7.68	7.38	10.05
V3	9.44	4.49	1.98	0.79			
Q4	8.47	4.98	1.87	2.07			7.29, 6.62
V5	8.92	4.16	1.83	0.81			
N6	9.49	4.32	3.02, 2.69				7.53, 6.85
G7	8.61	4.07, 3.57					
O8	7.69	4.60	1.80	1.78, 1.65	2.97		7.55
K9	8.45	4.89	1.65	1.21	1.34	2.44	7.09
I10	9.27	4.62	1.78	1.34, 1.10	0.79		
L11	8.25	3.81	1.21, -0.02	0.66	0.29, -0.44		
Q12	8.94	4.47	1.98, 1.74	2.16			7.23, 6.74
C13	8.83	4.95	2.95, 2.84				
C-Amide							7.43, 7.12

Table S8. *Chemical shift values (δ, ppm) for peptide* ***6***

Residue/ Group	Amide N–H	H_α	H_β	H_γ	H_δ	H_ε	Side chain N–H
N-Acetyl		1.96					
C0	8.27	5.10	2.87, 2.29				
R1	8.62	4.50	1.70	1.55, 1.39	3.05		7.00
W2	8.57	5.04	2.99, 2.83	7.13, 6.99	7.18	7.40	10.05
V3	9.44	4.48	1.95	0.77			
E4	8.47	4.94	1.90, 1.80	2.21			
V5	9.00	4.14	1.81	0.79			
N6	9.51	4.31	3.02, 2.65				7.54, 6.83
G7	8.55	4.01, 3.57					
O8	7.67	4.57	1.88	1.72, 1.62	2.96		7.54
K9	8.44	4.84	1.62	1.21	1.29	2.44	7.08
I10	9.30	4.62	1.76	1.30, 1.07	0.79		
L11	8.24	3.78	1.19, -0.04	0.66	0.28, -0.44		
Q12	8.95	4.46	2.05, 1.71	2.13			7.21, 6.72
C13	8.83	4.93	2.91, 2.83				
C-Amide							7.52, 7.11

Table S9. *Chemical shift values (δ, ppm) for peptide* ***7***

Residue/ Group	Amide N–H	H_α	H_β	H_γ	H_δ	H_ε	Side chain N–H
N-Acetyl		1.96					
C0	8.28	5.13	2.91, 2.31				
R1	8.62	4.52	1.73	1.58, 1.42	3.07		7.01
W2	8.58	5.04	2.99, 2.86	7.39, 7.12		7.18	10.05
V3	9.43	4.53	2.00	0.78			
T4	8.47	4.96	3.88	0.91			
V5	8.98	4.14	1.81	0.81			
N6	9.57	4.30	2.99, 2.68				7.53, 6.85
G7	8.40	4.06, 3.46					
O8	7.71	4.60	1.82	1.72, 1.63	2.97		7.56
K9	8.53	4.95	1.63	1.23	1.30	2.49	7.10
I10	9.41	4.60	1.76	1.34, 1.07	0.81		
L11	8.21	3.79	1.20, -0.07	0.66	0.27, -0.43		
Q12	8.93	4.47	2.02, 1.73	2.14			7.23, 6.72
C13	8.83	4.95	2.94, 2.83				
C-Amide							7.49, 7.11

Table S10. *Chemical shift values (δ, ppm) for peptide* ***8***

Residue/ Group	Amide N–H	H_α	H_β	H_γ	H_δ	H_ε	Side chain N–H
N-Acetyl		1.85					
R1	8.06	4.05	1.49	1.32	2.98		6.99
W2	8.06	4.62	3.22, 3.14	7.53, 7.40	7.13		10.04
V3	7.66	3.90	1.85	0.74			
Q4	8.19	4.15	2.20	1.89			
V5	8.15	3.99	1.98	0.83			
N6	8.42	4.60	2.74, 2.64				7.49, 6.81
C-Amide							7.14, 7.04

Table S11. *Chemical shift values (δ, ppm) for peptide* ***9***

Residue/ Group	Amide N–H	H_α	H_β	H_γ	H_δ	H_ε	Side chain N–H
N-Acetyl		1.85					
R1	8.06	4.05	1.50	1.31	2.98		6.98
W2	8.05	4.61	3.20, 3.11	7.53, 7.40	7.06		10.03
V3	7.66	3.91	1.83	0.72			
E4	8.14	4.17	1.95, 1.82	2.33			
V5	8.12	3.96	1.95	0.83			
N6	8.39	4.58	2.72, 2.62				7.48, 6.80
C-Amide							7.14, 7.03

Table S12. *Chemical shift values (δ, ppm) for peptide **10***

Residue/ Group	Amide N–H	H_α	H_β	H_γ	H_δ	H_ε	Side chain N–H
N-Acetyl		1.83					
R1	8.04	4.02	1.47	1.37	2.96		6.96
W2	8.00	4.67	3.24, 3.13	7.55, 7.40	7.07		10.04
V3	7.76	4.03	1.91	0.77			
T4	8.10	4.25	4.05	1.10			
V5	8.12	4.02	1.99	0.83			
N6	8.39	4.58	2.72, 2.62				7.48, 6.81
C-Amide							7.12, 7.03

Table S13. *Chemical shift values (δ, ppm) for peptide **11***

Residue/ Group	Amide N–H	H_α	H_β	H_γ	H_δ	H_ε	Side chain N–H
N-Acetyl		1.96					
G7	8.21	3.82					
O8	8.23	4.29	1.80	1.65	2.93		7.45
K9	8.30	4.21	1.66	1.31	1.30	2.92	7.51
I10	8.15	4.05	1.75	1.38, 1.11	0.79		
L11	8.28	4.29	1.61	1.48	0.82		
Q12	8.24	4.21	2.01, 1.91	2.28			7.44, 6.78
C-Amide							7.45, 7.03

2.2. Quantifying the extent of folding

To determine the chemical shifts of the fully folded state, 14-residue disulfide-linked analogs of peptides **1**, **2** and **3** were synthesized with a sequence of Ac-CRWVXVNGOKILQC-NH_2, where X represents glutamine/glutamic acid/threonine, and the C residues are lined via a cystine bridge. To determine the chemical shifts of each residue in the unfolded state, the 6-mer peptides were synthesized with sequences Ac-RWVXVN-NH_2, Ac-GOKILQ-NH_2, where X represents glutamine/glutamic acid/threonine. Note that for **4**, the peptides **5** and **11** were used as surrogates as *N*-methylation has minimal effects on the backbone amide shifts. The chemical shifts for residues in the strand and one turn residue were obtained from each 6-mer peptide. The fraction folded was determined from equation (1).

$$\text{Fraction Folded } (f) = [\delta_{obs} - \delta_0]/ [\delta_{100} - \delta_0] \qquad \textbf{(1)}$$

where δ_{obs} is the observed G7 split (in ppm) of the hairpin, δ_{100} is the G7 split (in ppm) of its cyclic form, and δ_0 is the G7 split of its corresponding 6-mer half.

Table S14. *Fraction folded of peptide **1-4** (room temperature).*

Peptide	δ_{obs} (ppm)	δ_0 (ppm)	δ_{100} (ppm)	Fraction folded
**1**	0.268	0	0.518	0.52
**2**	0.344	0	0.448	0.77
**3**	0.471	0	0.598	0.79
**4**	0.404	0	0.518	0.78

2.3. Structure prediction by AlphaFold and energy minimization

The sequences of beta hairpin **1**, **2**, **3**, and **5** (cyclic counterpart of **1**) were used to predict their 3-D structure using AlphaFold[4] with ColabFold[5] interface powered by Many-against-Many sequence searching (MMseqs2) cluster. The intrinsic cation-π interaction of the hairpin was characterized on the predicted structure using Gaussian 16 package with M06/6-31(d,p)[6] method. The optimized structure was then confirmed by ROESY-NMR[1]. More descriptions of the predicted structures are provided in Figure S3 and S4.

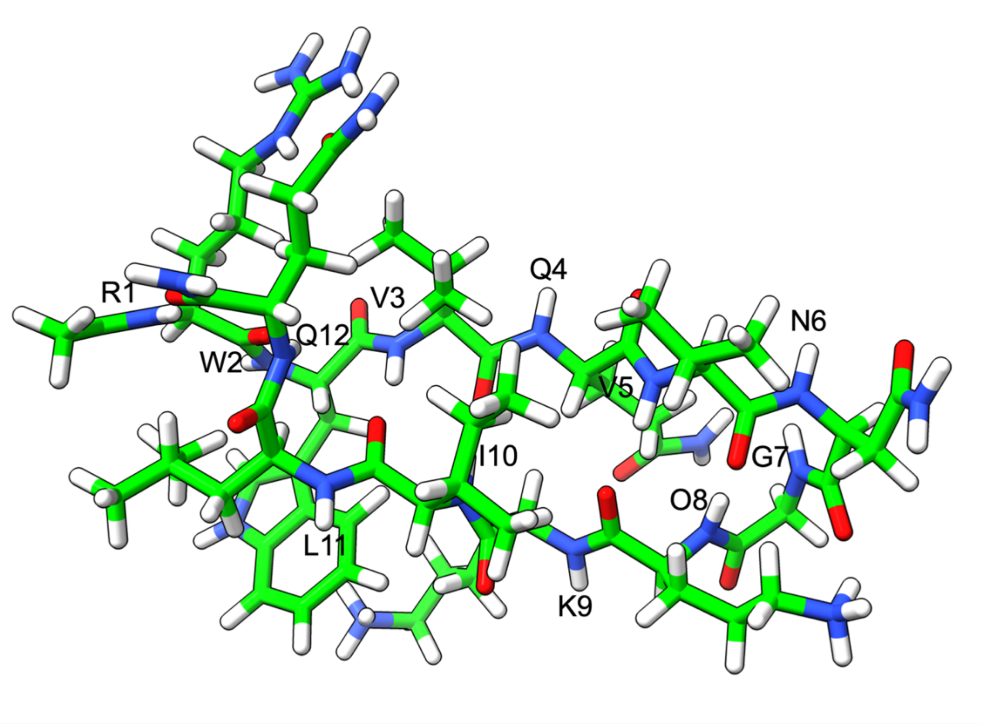

Figure S2. *Energy minimized structure of* ***1***. *All backbone amide protons are labelled.*

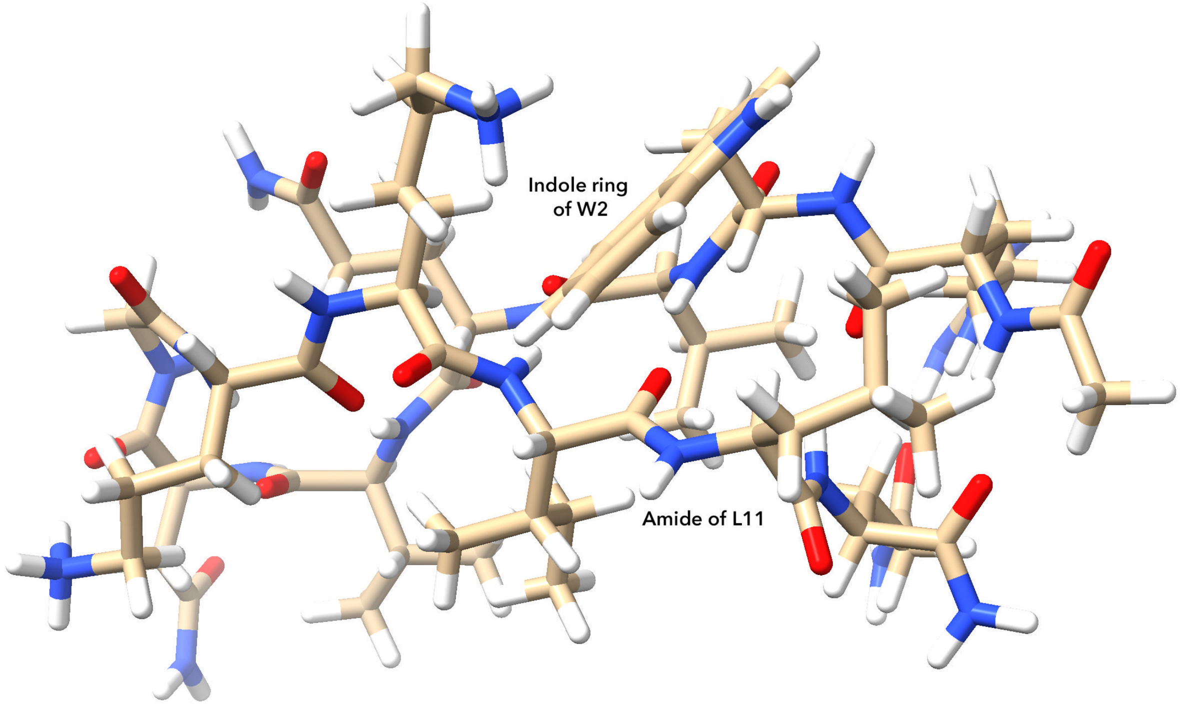

Figure S3. *Non-covalent interactions at the hydrophobic core K9 ammonium – W2 indole ring – L11 side chain of peptide 1, which are also observed by ROESY NMR data.*

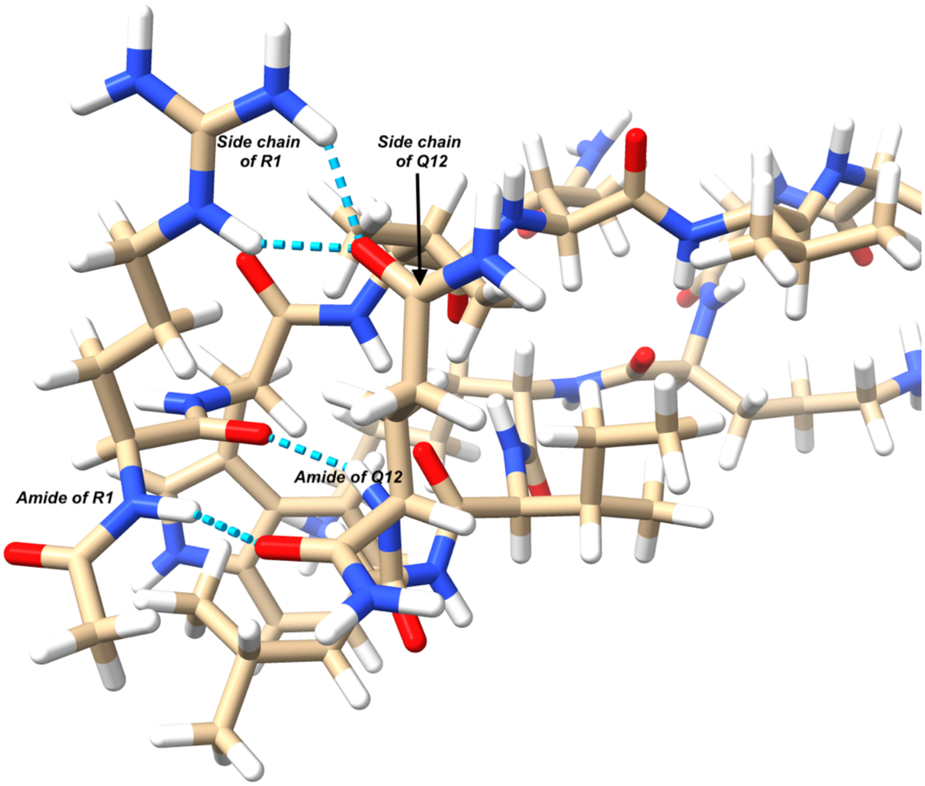

Figure S4. *Bifurcated side chain hydrogen bonding between R1 and Q12. Main chain and said chain hydrogen bonds are depicted by dashed cyan lines.*

*Table S15. Cartesian coordinates of the energy-minimized structure of **1***

Atom	x	y	z
H	9.205	0.935	2.573
C	9.414	0.032	1.985
H	10.491	-0.147	2.033
H	8.865	-0.801	2.430
C	9.021	0.294	0.557
O	9.732	0.945	-0.207
N	7.813	-0.202	0.182
H	7.241	-0.732	0.843
C	7.289	0.076	-1.135
H	7.792	0.984	-1.494
C	7.555	-1.053	-2.145
H	7.321	-0.686	-3.155
H	8.633	-1.264	-2.122
C	6.750	-2.312	-1.866
H	6.916	-2.646	-0.828
H	5.676	-2.097	-1.961
C	7.083	-3.439	-2.822
H	6.921	-3.099	-3.855
H	8.139	-3.735	-2.728
N	6.204	-4.566	-2.546
H	5.742	-4.622	-1.634
C	5.944	-5.556	-3.394
N	6.564	-5.622	-4.576
H	7.366	-5.046	-4.779
H	6.362	-6.373	-5.218
N	5.017	-6.462	-3.073
H	4.613	-6.423	-2.138
H	4.933	-7.312	-3.609
C	5.797	0.303	-1.030
O	5.111	-0.336	-0.230
N	5.255	1.179	-1.894
H	5.841	1.681	-2.549
C	3.819	1.367	-1.917
H	3.479	1.417	-0.873
C	3.458	2.680	-2.615
H	2.367	2.687	-2.769
H	3.916	2.698	-3.614
C	3.873	3.853	-1.795
C	4.987	4.635	-1.939
H	5.746	4.616	-2.712
N	5.061	5.548	-0.911
H	5.783	6.244	-0.801
C	3.99 0	5.369	-0.072
C	3.629	6.044	1.096
H	4.238	6.852	1.491
C	2.458	5.646	1.728
H	2.144	6.153	2.637
C	1.664	4.601	1.212
H	0.742	4.331	1.727
C	2.037	3.921	0.058
H	1.429	3.096	-0.320
C	3.214	4.305	-0.602
C	3.135	0.186	-2.601
O	3.497	-0.230	-3.697
N	2.096	-0.286	-1.889
H	1.881	0.153	-0.996
C	1.142	-1.253	-2.371
H	1.250	-1.309	-3.465
C	1.394	-2.658	-1.784
H	0.717	-3.356	-2.302
C	2.827	-3.097	-2.058
H	3.090	-3.006	-3.119
H	2.985	-4.135	-1.741
H	3.525	-2.468	-1.484
C	1.101	-2.703	-0.290
H	0.049	-2.487	-0.064
H	1.328	-3.696	0.117
H	1.720	-1.965	0.243
C	-0.243	-0.690	-2.046
O	-0.366	0.450	-1.591
N	-1.311	-1.478	-2.258
H	-1.235	-2.407	-2.658
C	-2.644	-0.996	-1.945
H	-2.560	-0.301	-1.101
C	-3.243	-0.259	-3.146
H	-2.604	0.612	-3.346
H	-3.189	-0.919	-4.023
C	-4.674	0.212	-2.917
H	-5.388	-0.618	-3.007
H	-4.768	0.598	-1.894
C	-5.040	1.372	-3.816
O	-4.370	2.398	-3.846
N	-6.177	1.223	-4.542
H	-6.598	0.315	-4.674
H	-6.407	1.944	-5.211
C	-3.502	-2.180	-1.538
O	-3.441	-3.244	-2.146
N	-4.329	-1.933	-0.497
H	-4.310	-1.021	-0.035
C	-5.295	-2.902	-0.016
H	-5.377	-3.691	-0.776
C	-4.868	-3.525	1.324
H	-4.732	-2.687	2.027
C	-5.955	-4.448	1.861
H	-6.875	-3.909	2.121
H	-6.212	-5.223	1.125
H	-5.612	-4.956	2.768
C	-3.549	-4.265	1.171
H	-3.227	-4.673	2.135
H	-2.753	-3.607	0.803
H	-3.653	-5.101	0.467
C	-6.610	-2.165	0.155
O	-6.661	-1.126	0.822
N	-7.698	-2.677	-0.454
H	-7.657	-3.605	-0.859
C	-9.029	-2.152	-0.169
H	-9.695	-2.610	-0.911
C	-9.511	-2.555	1.211
H	-10.491	-2.104	1.413
H	-8.825	-2.181	1.984
C	-9.629	-4.065	1.312

O	-9.529	-4.796	0.332
N	-9.842	-4.532	2.559
H	-9.962	-3.92 0	3.352
H	-9.975	-5.524	2.692
C	-9.090	-0.645	-0.401
O	-9.684	0.124	0.357
N	-8.508	-0.214	-1.538
H	-8.070	-0.893	-2.147
C	-8.566	1.171	-1.944
H	-9.570	1.570	-1.767
H	-8.371	1.237	-3.019
C	-7.605	2.124	-1.250
O	-7.704	3.327	-1.457
N	-6.687	1.567	-0.423
H	-6.719	0.568	-0.225
C	-5.889	2.370	0.488
H	-6.119	3.419	0.265
C	-6.223	2.020	1.940
H	-5.88 0	0.988	2.098
H	-5.625	2.652	2.607
C	-7.718	2.134	2.252
H	-7.943	3.067	2.783
H	-8.294	2.155	1.318
C	-8.194	0.941	3.057
H	-7.875	0.979	4.101
H	-7.835	0.008	2.607
N	-9.691	0.86 0	3.031
H	-10.128	1.752	3.275
H	-10.048	0.161	3.685
H	-9.980	0.587	2.070
C	-4.426	2.053	0.223
O	-3.988	0.917	0.439
N	-3.656	3.029	-0.284
H	-4.021	3.968	-0.376
C	-2.246	2.761	-0.512
H	-2.181	1.758	-0.946
C	-1.604	3.713	-1.513
H	-2.245	3.732	-2.408
H	-0.658	3.245	-1.829
C	-1.315	5.126	-1.028
H	-2.232	5.634	-0.696
H	-0.666	5.074	-0.141
C	-0.654	5.947	-2.128
H	0.188	5.388	-2.568
H	-1.364	6.105	-2.949
C	-0.172	7.307	-1.672
H	0.069	7.964	-2.509
H	-0.905	7.809	-1.035
N	1.092	7.192	-0.861
H	1.866	6.837	-1.432
H	1.002	6.549	-0.058
H	1.383	8.100	-0.490
C	-1.533	2.749	0.843
O	-1.775	3.591	1.704
N	-0.638	1.750	0.986
H	-0.464	1.123	0.200

C	0.082	1.517	2.219
H	0.038	2.447	2.803
C	-0.549	0.388	3.067
H	0.083	0.291	3.966
C	-1.954	0.779	3.506
H	-2.340	0.073	4.249
H	-2.646	0.780	2.652
H	-1.969	1.779	3.953
C	-0.523	-0.951	2.330
H	-1.078	-0.849	1.378
H	0.514	-1.188	2.054
C	-1.097	-2.108	3.131
H	-0.913	-3.062	2.623
H	-0.635	-2.168	4.125
H	-2.180	-2.016	3.272
C	1.525	1.199	1.865
O	1.818	0.663	0.796
N	2.454	1.511	2.788
H	2.178	1.962	3.653
C	3.870	1.254	2.551
H	3.993	1.200	1.462
C	4.749	2.346	3.126
H	4.334	3.328	2.846
H	4.716	2.274	4.223
C	6.197	2.241	2.631
H	6.474	1.169	2.588
C	6.345	2.828	1.232
H	7.350	2.644	0.826
H	6.188	3.917	1.259
H	5.616	2.418	0.520
C	7.137	2.942	3.600
H	8.171	2.942	3.232
H	7.125	2.467	4.588
H	6.836	3.992	3.728
C	4.216	-0.110	3.157
O	4.485	-0.250	4.345
N	4.146	-1.142	2.287
H	4.161	-0.925	1.292
C	4.563	-2.464	2.692
H	3.955	-2.783	3.550
C	4.353	-3.418	1.520
H	3.299	-3.354	1.222
H	4.947	-3.061	0.665
C	4.711	-4.860	1.854
H	5.796	-4.966	1.992
H	4.242	-5.164	2.799
C	4.315	-5.800	0.745
O	4.569	-5.540	-0.439
N	3.683	-6.923	1.104
H	3.467	-7.134	2.066
H	3.424	-7.595	0.395
C	6.040	-2.458	3.088
O	6.883	-1.915	2.379
N	6.350	-3.141	4.202
H	5.641	-3.491	4.829
H	7.314	-3.182	4.500

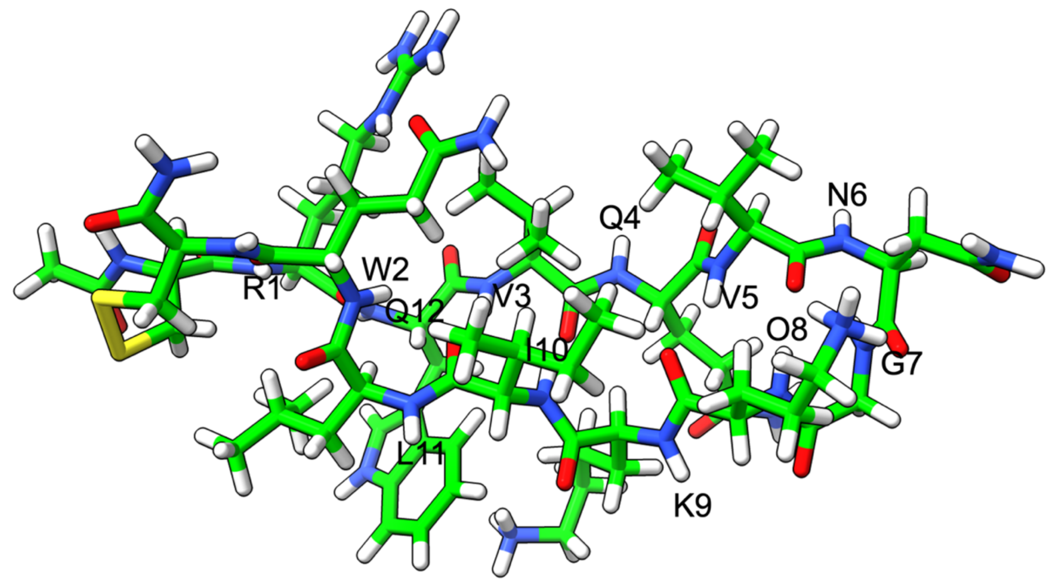

Figure S5. *Energy minimized structure of **5**. All backbone amide protons are labelled*

Table S16. *Cartesian coordinates of the energy-minimized structure of **5***

Atom	x	y	z
C	-11.118	-0.569	-1.108
C	-12.558	-0.242	-1.412
O	-10.699	-1.720	-1.131
H	-12.737	0.816	-1.618
H	-12.882	-0.838	-2.268
H	-13.173	-0.536	-0.555
N	-10.350	0.497	-0.755
C	-8.946	0.412	-0.446
C	-8.112	0.165	-1.719
C	-8.593	-0.654	0.595
O	-8.626	-0.100	-2.796
S	-9.664	-0.755	2.076
H	-10.792	1.404	-0.707
H	-8.643	1.377	-0.040
H	-8.627	-1.623	0.098
H	-7.570	-0.479	0.927
N	-6.770	0.227	-1.540
C	-3.093	5.203	-4.209
N	-2.259	6.069	-3.636
C	-5.874	-0.170	-2.601
C	-4.575	-0.629	-1.973
C	-5.549	0.957	-3.597
O	-4.207	-0.177	-0.890
C	-4.706	2.079	-3.010
C	-4.436	3.151	-4.046
N	-3.610	4.210	-3.485
N	-3.387	5.315	-5.507
H	-6.395	0.552	-0.661
H	-6.318	-1.004	-3.144
H	-5.006	0.525	-4.438
H	-6.484	1.379	-3.965
H	-3.757	1.669	-2.665
H	-5.234	2.521	-2.165
H	-3.919	2.705	-4.896
H	-5.383	3.573	-4.382
H	-3.418	4.192	-2.494
H	-2.030	5.977	-2.657
H	-1.856	6.818	-4.181
H	-2.985	6.063	-6.053
H	-4.012	4.651	-5.942
N	-3.831	-1.483	-2.705
C	-2.433	-1.680	-2.392
C	-1.579	-0.627	-3.090
C	-1.960	-3.077	-2.813
O	-1.835	-0.227	-4.222
C	-2.518	-4.151	-1.946
C	-3.545	-5.013	-2.221
C	-2.080	-4.458	-0.615
C	-2.883	-5.528	-0.145

C	-1.080	-3.938	0.219
N	-3.767	-5.843	-1.145
C	-1.699	-5.566	1.921
C	-2.706	-6.089	1.122
C	-0.888	-4.502	1.476
H	-4.251	-1.986	-3.473
H	-2.300	-1.578	-1.315
H	-2.275	-3.258	-3.841
H	-0.872	-3.112	-2.765
H	-4.100	-5.038	-3.147
H	-0.468	-3.111	-0.110
H	-1.533	-5.982	2.904
H	-3.330	-6.900	1.467
H	-0.108	-4.120	2.119
H	-4.469	-6.568	-1.099
N	-0.505	-0.266	-2.360
C	0.633	0.444	-2.885
C	1.877	-0.290	-2.405
C	0.690	1.912	-2.402
O	1.808	-1.127	-1.501
C	-0.613	2.625	-2.731
C	0.997	1.993	-0.911
H	-0.498	-0.509	-1.380
H	0.600	0.424	-3.974
H	1.494	2.413	-2.940
H	-0.560	3.658	-2.386
H	-0.773	2.610	-3.809
H	-1.440	2.118	-2.234
H	1.934	1.475	-0.704
H	1.087	3.038	-0.615
H	0.190	1.524	-0.348
N	3.052	0.097	-2.930
C	4.303	-0.307	-2.305
C	5.250	0.872	-2.463
C	4.858	-1.591	-2.906
O	5.455	1.362	-3.568
C	6.070	-2.119	-2.146
C	6.369	-3.555	-2.526
N	7.644	-3.821	-2.879
O	5.499	-4.420	-2.487
H	3.067	0.670	-3.761
H	4.124	-0.469	-1.242
H	4.077	-2.351	-2.888
H	5.144	-1.402	-3.941
H	6.936	-1.501	-2.383
H	5.873	-2.065	-1.075
H	8.331	-3.080	-2.888
H	7.912	-4.761	-3.134
N	5.791	1.331	-1.309
C	6.530	2.580	-1.270
C	7.806	2.315	-0.502
C	5.730	3.706	-0.602
O	7.757	1.987	0.693
C	6.571	4.975	-0.539
C	4.424	3.948	-1.343
H	5.678	0.795	-0.461
H	6.782	2.877	-2.288

H	5.494	3.400	0.417
H	5.996	5.769	-0.063
H	6.845	5.280	-1.549
H	7.474	4.784	0.04 0
H	3.871	4.750	-0.854
H	3.826	3.036	-1.333
H	4.638	4.230	-2.374
N	8.967	2.388	-1.169
C	10.244	2.125	-0.514
C	10.175	0.821	0.293
C	10.716	3.302	0.319
O	10.494	0.751	1.479
C	12.138	3.091	0.799
N	12.457	3.715	1.952
O	12.933	2.414	0.158
H	8.957	2.631	-2.149
H	10.982	1.975	-1.302
H	10.675	4.207	-0.287
H	10.060	3.418	1.181
H	11.764	4.268	2.436
H	13.389	3.629	2.332
N	9.767	-0.251	-0.419
C	9.728	-1.588	0.129
C	8.478	-1.975	0.902
O	8.348	-3.120	1.314
H	9.476	-0.113	-1.376
H	9.833	-2.287	-0.700
H	10.587	-1.711	0.788
N	7.587	-0.983	1.145
C	6.550	-1.131	2.153
C	5.210	-1.245	1.447
C	6.589	0.053	3.108
O	4.667	-0.261	0.933
C	7.881	0.053	3.924
C	8.299	1.418	4.426
N	8.882	2.226	3.305
H	7.645	-0.125	0.616
H	6.732	-2.046	2.716
H	6.53 0	0.977	2.533
H	5.737	-0.004	3.785
H	7.741	-0.600	4.786
H	8.683	-0.352	3.307
H	7.428	1.936	4.828
H	9.044	1.301	5.213
H	9.787	1.853	3.056
H	8.984	3.187	3.600
H	8.268	2.183	2.504
N	4.707	-2.490	1.374
C	3.459	-2.803	0.689
C	2.315	-2.571	1.683
C	3.491	-4.229	0.168
O	2.231	-3.238	2.711
C	2.386	-4.475	-0.848
C	2.425	-5.873	-1.452
C	2.031	-6.989	-0.508
N	0.558	-6.942	-0.196
H	5.217	-3.243	1.814

H	3.338	-2.122	-0.153
H	4.455	-4.411	-0.306
H	3.368	-4.918	1.004
H	1.424	-4.338	-0.353
H	2.476	-3.743	-1.650
H	1.743	-5.893	-2.302
H	3.435	-6.066	-1.814
H	2.594	-6.886	0.419
H	2.27 0	-7.948	-0.968
H	0.323	-7.695	0.434
H	0.030	-7.041	-1.051
H	0.334	-6.058	0.238
N	1.480	-1.560	1.359
C	0.530	-0.988	2.291
C	-0.847	-0.999	1.652
C	0.918	0.467	2.633
O	-0.999	-0.580	0.501
C	2.278	0.534	3.329
C	-0.163	1.155	3.459
C	2.948	1.889	3.165
H	1.520	-1.185	0.422
H	0.512	-1.584	3.204
H	1.006	1.011	1.693
H	2.137	0.34 0	4.392
H	2.928	-0.235	2.912
H	-1.117	1.090	2.936
H	0.101	2.203	3.602
H	-0.246	0.665	4.429
H	3.91 0	1.884	3.678
H	2.312	2.663	3.595
H	3.102	2.092	2.105
N	-1.868	-1.442	2.408
C	-3.251	-1.348	1.955
C	-3.793	0.011	2.408
C	-4.126	-2.473	2.465
O	-4.413	0.173	3.455
C	-5.500	-2.439	1.783
C	-5.460	-3.091	0.407
C	-6.556	-3.087	2.664
H	-1.672	-1.850	3.311

H	-3.258	-1.375	0.865
H	-3.644	-3.427	2.252
H	-4.256	-2.368	3.542
H	-5.777	-1.394	1.647
H	-4.696	-2.608	-0.202
H	-6.432	-2.982	-0.075
H	-5.224	-4.150	0.513
H	-7.522	-3.051	2.160
H	-6.620	-2.549	3.610
H	-6.283	-4.125	2.854
N	-3.473	1.039	1.585
C	-4.025	2.346	1.857
C	-5.547	2.291	1.817
C	-3.543	3.346	0.816
O	-6.150	1.752	0.895
C	-2.050	3.603	0.912
C	-1.587	4.657	-0.061
N	-0.396	5.215	0.198
O	-2.257	4.969	-1.053
H	-2.860	0.894	0.796
H	-3.703	2.673	2.846
H	-3.768	2.955	-0.176
H	-4.075	4.287	0.955
H	-1.816	3.933	1.924
H	-1.516	2.674	0.709
H	0.129	4.929	1.012
H	-0.024	5.921	-0.421
N	-6.191	2.939	2.821
C	-7.637	2.873	2.865
C	-8.274	4.055	3.578
C	-8.142	1.549	3.431
O	-9.37 0	3.928	4.112
S	-9.822	1.194	2.771
H	-5.675	3.454	3.520
H	-7.979	2.916	1.831
H	-7.462	0.748	3.141
H	-8.185	1.612	4.518
N	-7.586	5.211	3.540
H	-8.012	6.04 0	3.929
H	-6.722	5.302	3.028

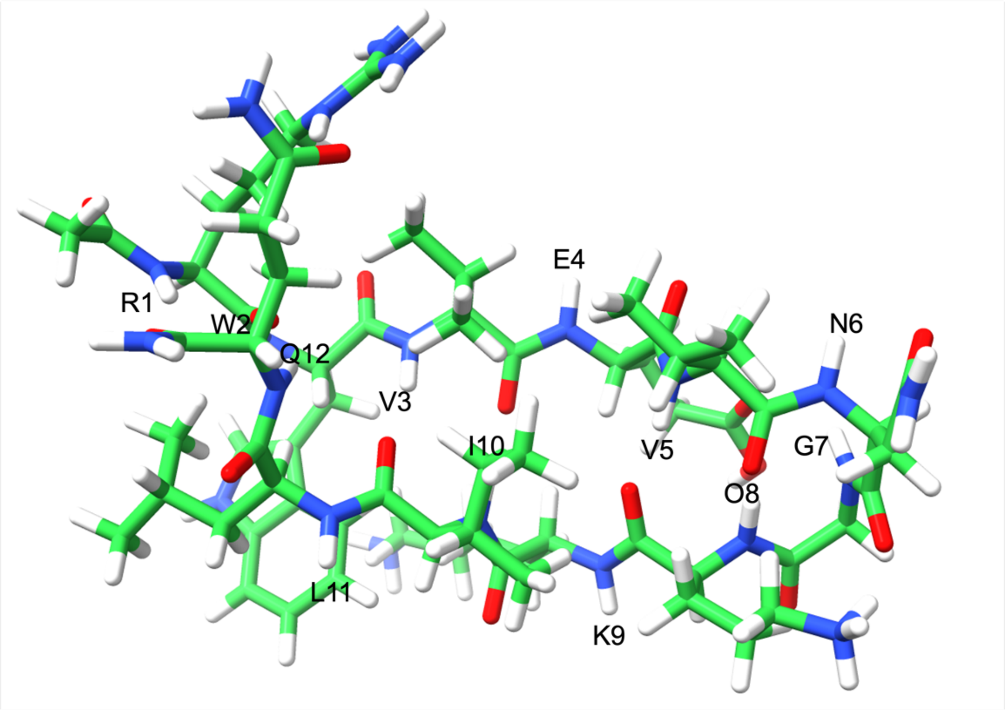

Figure S6. *Energy minimized structure of* ***2***. *All backbone amide protons are labelled.*

Table S17. *Cartesian coordinates of the energy-minimized structure of* ***2***

Atom	x	y	z
N	7.599	-1.215	-0.085
C	7.064	-0.634	-1.297
C	5.690	-0.070	-1.006
C	6.883	-1.657	-2.439
O	4.919	-0.636	-0.226
C	6.166	-2.912	-1.974
C	5.659	-3.766	-3.118
N	4.926	-4.881	-2.536
N	4.153	-5.753	-4.535
N	3.405	-6.585	-2.510
C	4.168	-5.744	-3.204
N	5.320	1.023	-1.696
C	3.917	1.387	-1.725
C	3.110	0.292	-2.424
C	3.726	2.727	-2.438
O	3.523	-0.263	-3.438
C	4.274	3.859	-1.640
C	5.456	4.529	-1.811
C	3.659	4.413	-0.467
C	4.521	5.426	0.018
C	2.449	4.163	0.196
N	5.614	5.466	-0.815
C	2.993	5.934	1.771
C	4.202	6.193	1.140
C	2.123	4.931	1.305
N	1.923	0.053	-1.836
C	0.944	-0.870	-2.353

C	-0.431	-0.219	-2.199
C	0.998	-2.245	-1.645
O	-0.590	0.825	-1.568
C	2.347	-2.915	-1.853
C	0.699	-2.112	-0.160
N	-1.453	-0.874	-2.782
C	-2.831	-0.414	-2.680
C	-3.605	-1.647	-2.236
C	-3.337	0.145	-4.012
O	-3.665	-2.632	-2.966
C	-4.378	1.240	-3.878
C	-5.730	0.807	-3.380
O	-6.031	-0.310	-3.017
O	-6.655	1.777	-3.362
N	-4.087	-1.601	-0.979
C	-4.839	-2.704	-0.421
C	-6.166	-2.136	0.028
C	-4.105	-3.370	0.749
O	-6.211	-1.183	0.814
C	-4.973	-4.442	1.394
C	-2.781	-3.945	0.269
N	-7.280	-2.700	-0.479
C	-8.581	-2.396	0.097
C	-8.847	-0.897	0.069
C	-8.744	-2.999	1.479
O	-9.399	-0.308	1.001
C	-8.672	-4.513	1.412
N	-8.563	-5.125	2.609

O	-8.704	-5.124	0.349
N	-8.541	-0.278	-1.087
C	-8.813	1.128	-1.256
C	-7.839	2.074	-0.576
O	-8.116	3.265	-0.497
N	-6.693	1.533	-0.097
C	-5.775	2.304	0.711
C	-4.380	2.228	0.106
C	-5.731	1.750	2.141
O	-3.869	1.137	-0.159
C	-7.122	1.692	2.781
C	-7.358	0.372	3.488
N	-3.746	3.402	-0.083
C	-2.338	3.467	-0.459
C	-1.531	3.411	0.838
C	-2.038	4.745	-1.224
O	-1.628	4.308	1.673
C	-0.548	4.883	-1.499
C	-0.209	6.113	-2.327
C	1.290	6.280	-2.432
N	1.638	7.491	-3.249
N	-0.774	2.305	0.985
C	-0.090	2.007	2.223
C	1.328	1.569	1.902
C	-0.835	0.943	3.064
O	1.616	1.040	0.829
C	-0.833	-0.419	2.374
C	-2.247	1.421	3.373
C	-1.515	-1.518	3.172
N	2.242	1.767	2.869
C	3.647	1.458	2.646
C	3.964	0.117	3.301
C	4.521	2.586	3.166
O	4.182	0.013	4.503
C	6.019	2.401	2.921
C	6.322	2.046	1.470
C	6.751	3.673	3.327
N	3.937	-0.942	2.457
C	4.421	-2.229	2.911
C	5.870	-2.083	3.384
C	4.371	-3.227	1.756
O	6.692	-1.465	2.710
C	4.742	-5.575	0.982
N	5.748	-6.455	0.901
O	3.843	-5.519	0.128
N	-8.827	0.172	3.718
C	4.757	-4.640	2.165
H	7.732	0.164	-1.651
H	6.303	-1.170	-3.238
H	7.870	-1.900	-2.848
H	6.822	-3.521	-1.332
H	5.300	-2.633	-1.356
H	4.989	-3.170	-3.759

H	6.490	-4.131	-3.739
H	4.768	-4.870	-1.527
H	3.616	-6.441	-5.041
H	4.802	-5.192	-5.064
H	2.861	-7.302	-2.963
H	3.406	-6.516	-1.496
H	5.943	1.411	-2.394
H	3.566	1.482	-0.688
H	2.645	2.864	-2.598
H	4.185	2.671	-3.435
H	6.210	4.408	-2.580
H	1.773	3.380	-0.156
H	6.388	6.107	-0.737
H	2.712	6.521	2.642
H	4.871	6.971	1.500
H	1.172	4.768	1.814
H	1.678	0.550	-0.980
H	1.155	-1.015	-3.423
H	0.222	-2.878	-2.104
H	3.144	-2.301	-1.409
H	2.574	-3.056	-2.918
H	2.368	-3.892	-1.354
H	1.460	-1.489	0.334
H	0.699	-3.096	0.325
H	-0.280	-1.649	0.024
H	-1.282	-1.702	-3.341
H	-2.844	0.361	-1.904
H	-3.716	-0.675	-4.635
H	-2.477	0.571	-4.540
H	-4.534	1.725	-4.851
H	-4.020	2.039	-3.208
H	-4.112	-0.709	-0.481
H	-4.981	-3.443	-1.223
H	-3.907	-2.575	1.488
H	-5.296	-5.185	0.650
H	-5.872	-4.024	1.866
H	-4.416	-4.974	2.173
H	-2.950	-4.751	-0.458
H	-2.213	-4.364	1.108
H	-2.159	-3.179	-0.212
H	-7.211	-3.578	-0.981
H	-9.319	-2.859	-0.570
H	-9.712	-2.707	1.905
H	-7.971	-2.617	2.159
H	-8.568	-6.134	2.642
H	-8.589	-4.616	3.479
H	-7.959	-0.738	-1.784
H	-9.805	1.364	-0.860
H	-8.808	1.365	-2.323
H	-6.548	0.525	-0.115
H	-6.146	3.336	0.705
H	-5.041	2.358	2.741
H	-5.295	0.743	2.079

H	-7.890	1.786	2.001
H	-7.272	2.528	3.475
H	-6.852	0.309	4.455
H	-7.035	-0.464	2.859
H	-4.172	4.252	0.267
H	-2.119	2.589	-1.081
H	-2.613	4.742	-2.160
H	-2.379	5.608	-0.632
H	-0.011	4.947	-0.542
H	-0.173	3.976	-2.002
H	-0.651	6.030	-3.330
H	-0.648	7.005	-1.856
H	1.760	6.415	-1.451
H	1.766	5.426	-2.924
H	1.263	8.347	-2.829
H	1.257	7.427	-4.198
H	2.650	7.613	-3.334
H	-0.694	1.642	0.213
H	-0.066	2.941	2.802
H	-0.273	0.849	4.009
H	-1.326	-0.321	1.389
H	0.205	-0.719	2.172
H	-2.866	1.373	2.465
H	-2.255	2.454	3.737
H	-2.724	0.790	4.131
H	-2.592	-1.342	3.277
H	-1.093	-1.596	4.183
H	-1.384	-2.490	2.681
H	1.990	2.262	3.716
H	3.763	1.365	1.559
H	4.183	3.512	2.671

H	4.340	2.708	4.244
H	6.370	1.571	3.555
H	5.862	2.770	0.778
H	5.945	1.048	1.217
H	7.404	2.042	1.285
H	6.444	4.508	2.680
H	7.836	3.557	3.232
H	6.528	3.955	4.364
H	3.964	-0.764	1.454
H	3.796	-2.579	3.746
N	6.185	-2.727	4.521
H	5.045	-2.866	0.966
H	3.360	-3.228	1.325
H	-9.264	0.990	4.149
H	-9.013	-0.625	4.330
H	-9.278	-0.010	2.798
H	5.744	-4.659	2.646
H	4.044	-5.030	2.906
H	7.128	-2.658	4.876
H	5.476	-3.113	5.126
H	5.755	-7.136	0.153
H	6.476	-6.505	1.598
C	8.833	-1.781	-0.065
H	7.015	-1.245	0.752
C	9.179	-2.496	1.214
H	8.580	-3.413	1.289
H	8.936	-1.890	2.091
H	10.237	-2.762	1.208
O	9.588	-1.742	-1.033
H	-6.291	2.623	-3.661

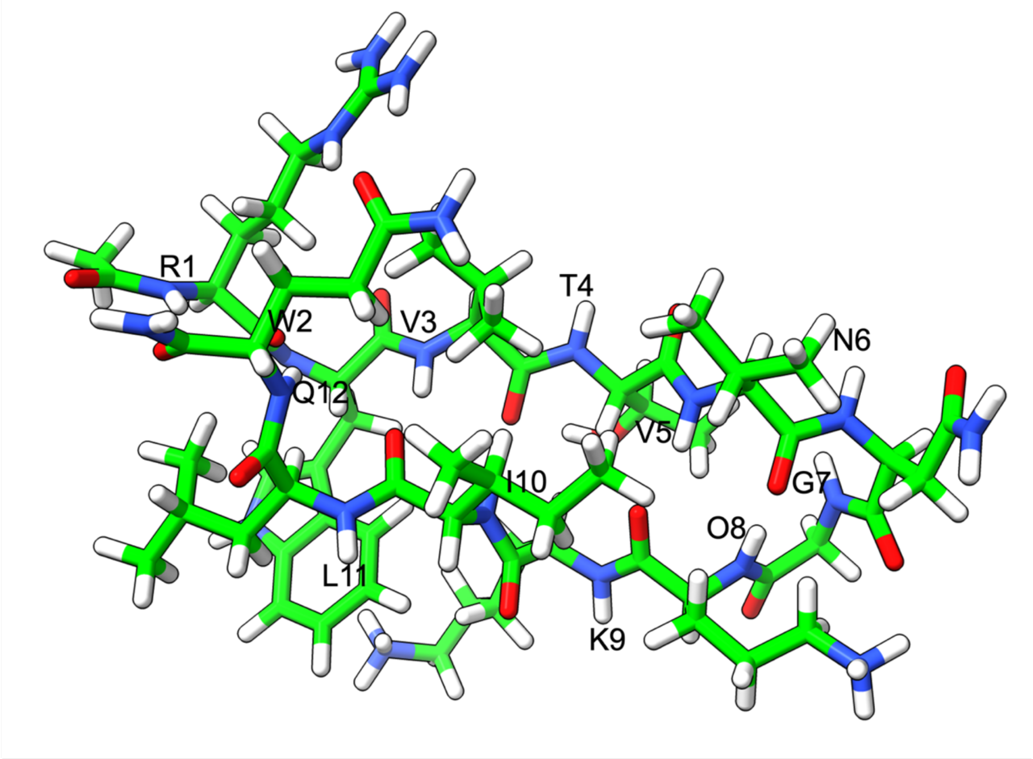

Figure S7. *Energy minimized structure of **3**. All backbone amide protons are labelled.*

Table S18. *Cartesian coordinates of the energy-minimized structure of **3***

Atom	x	y	z
H	9.877	-1.218	-2.347
C	10.150	-0.582	-1.499
H	11.213	-0.704	-1.290
H	9.968	0.458	-1.795
C	9.382	-0.918	-0.244
O	9.955	-1.127	0.819
N	8.022	-0.954	-0.337
H	7.519	-1.080	0.545
C	7.246	-0.740	-1.538
H	7.748	0.003	-2.172
C	7.026	-2.018	-2.374
H	6.488	-1.740	-3.292
H	8.007	-2.402	-2.684
C	6.256	-3.097	-1.633
H	5.268	-2.718	-1.338
H	6.784	-3.364	-0.705
C	6.045	-4.342	-2.469
H	5.520	-4.079	-3.401
H	7.009	-4.799	-2.739
N	5.238	-5.275	-1.696
H	4.965	-5.019	-0.745
C	4.743	-6.418	-2.163
N	5.037	-6.828	-3.399
H	5.716	-6.343	-3.964
H	4.686	-7.707	-3.746
N	3.933	-7.138	-1.388
H	3.716	-6.789	-0.454
H	3.594	-8.039	-1.684
C	5.874	-0.219	-1.154
O	5.293	-0.649	-0.158
N	5.310	0.657	-2.009
H	5.789	0.862	-2.878
C	3.882	0.890	-1.955
H	3.602	1.025	-0.901
C	3.504	2.152	-2.740
H	2.409	2.156	-2.852
H	3.916	2.076	-3.756
C	3.953	3.399	-2.059
C	5.106	4.107	-2.270
H	5.890	3.940	-2.999
N	5.189	5.156	-1.383
H	5.942	5.827	-1.345
C	4.083	5.146	-0.571
C	3.718	5.990	0.479
H	4.347	6.827	0.776
C	2.528	5.713	1.142
H	2.225	6.338	1.979
C	1.712	4.628	0.758
H	0.791	4.433	1.308
C	2.077	3.797	-0.295
H	1.450	2.947	-0.570
C	3.279	4.046	-0.970
C	3.136	-0.319	-2.523
O	3.547	-0.934	-3.502
N	1.988	-0.574	-1.868

H	1.768	-0.015	-1.045
C	0.971	-1.488	-2.322
H	1.184	-1.724	-3.375
C	0.960	-2.804	-1.514
H	0.274	-3.496	-2.026
C	2.345	-3.436	-1.520
H	2.747	-3.529	-2.536
H	2.317	-4.433	-1.064
H	3.047	-2.818	-0.938
C	0.466	-2.588	-0.088
H	-0.587	-2.273	-0.055
H	0.548	-3.513	0.495
H	1.065	-1.812	0.413
C	-0.361	-0.746	-2.239
O	-0.405	0.455	-1.933
N	-1.479	-1.448	-2.476
H	-1.448	-2.426	-2.748
C	-2.792	-0.932	-2.109
H	-2.672	-0.239	-1.266
C	-3.437	-0.163	-3.281
H	-3.249	-0.773	-4.185
C	-4.929	0.047	-3.138
H	-5.279	0.686	-3.954
H	-5.468	-0.906	-3.196
H	-5.167	0.538	-2.185
O	-2.843	1.113	-3.412
H	-1.900	1.042	-3.194
C	-3.642	-2.115	-1.686
O	-3.575	-3.184	-2.288
N	-4.459	-1.870	-0.640
H	-4.463	-0.942	-0.209
C	-5.480	-2.811	-0.215
H	-5.594	-3.554	-1.016
C	-5.116	-3.529	1.096
H	-4.923	-2.740	1.840
C	-6.277	-4.389	1.581
H	-7.145	-3.789	1.882
H	-5.975	-4.980	2.452
H	-6.604	-5.091	0.801
C	-3.858	-4.365	0.919
H	-3.012	-3.767	0.564
H	-4.029	-5.173	0.195
H	-3.570	-4.822	1.873
C	-6.754	-2.008	-0.029
O	-6.755	-0.998	0.682
N	-7.850	-2.438	-0.685
H	-7.830	-3.351	-1.126
C	-9.173	-1.879	-0.429
H	-9.831	-2.326	-1.186
C	-9.702	-2.265	0.940
H	-9.042	-1.881	1.730
H	-10.678	-1.793	1.101
C	-9.849	-3.770	1.051
O	-9.761	-4.512	0.077
N	-10.080	-4.226	2.300
H	-10.189	-3.607	3.089
H	-10.236	-5.214	2.437

C	-9.220	-0.370	-0.652
O	-9.942	0.355	0.024
N	-8.463	0.092	-1.677
H	-7.932	-0.571	-2.226
C	-8.447	1.486	-2.047
H	-8.249	1.578	-3.119
H	-9.436	1.921	-1.863
C	-7.447	2.375	-1.321
O	-7.378	3.563	-1.611
N	-6.709	1.788	-0.348
H	-6.768	0.782	-0.188
C	-5.764	2.539	0.457
H	-5.983	3.602	0.291
C	-5.849	2.190	1.945
H	-5.048	2.741	2.455
H	-5.608	1.122	2.055
C	-7.179	2.492	2.632
C	-4.359	2.186	-0.006
O	-3.972	1.015	0.056
N	-3.580	3.185	-0.464
H	-3.893	4.141	-0.350
C	-2.176	2.915	-0.722
H	-2.145	1.933	-1.199
C	-1.544	3.897	-1.699
H	-0.579	3.464	-2.007
H	-2.172	3.913	-2.601
C	-1.306	5.314	-1.201
H	-2.239	5.781	-0.852
H	-0.640	5.284	-0.326
C	-0.707	6.169	-2.311
H	0.121	5.630	-2.799
H	-1.457	6.330	-3.095
C	-0.217	7.525	-1.852
H	-0.085	8.224	-2.680
H	-0.888	7.980	-1.118
N	1.130	7.410	-1.191
H	1.439	8.305	-0.803
H	1.151	6.723	-0.420
H	1.843	7.113	-1.864
C	-1.445	2.837	0.620
O	-1.687	3.618	1.536
N	-0.542	1.833	0.693
H	-0.448	1.206	-0.107
C	0.074	1.397	1.933
H	-0.116	2.170	2.690
C	-0.547	0.054	2.364
H	-0.520	-0.574	1.457
C	0.264	-0.636	3.453
H	-0.231	-1.560	3.773
H	1.274	-0.906	3.115
H	0.364	0.010	4.336
C	-2.011	0.236	2.769
H	-2.053	0.545	3.825
H	-2.465	1.049	2.189
C	-2.837	-1.018	2.539
H	-3.888	-0.863	2.817
H	-2.466	-1.881	3.108

H	-2.816	-1.291	1.474
C	1.558	1.218	1.679
O	1.939	0.546	0.715
N	2.413	1.784	2.545
H	2.057	2.326	3.323
C	3.858	1.659	2.381
H	4.019	1.431	1.320
C	4.559	2.954	2.745
H	4.114	3.755	2.134
H	4.347	3.190	3.799
C	6.074	2.937	2.528
H	6.518	2.258	3.274
C	6.458	2.429	1.142
H	7.532	2.571	0.963
H	6.249	1.359	1.027
H	5.914	2.976	0.354
C	6.625	4.338	2.757
H	7.719	4.351	2.697
H	6.240	5.026	1.989
H	6.333	4.734	3.737
C	4.337	0.479	3.227
O	4.586	0.590	4.423
N	4.404	-0.694	2.555
H	4.423	-0.652	1.536
C	5.034	-1.837	3.170
H	4.584	-1.984	4.163

C	4.820	-3.085	2.318
H	5.395	-3.913	2.750
H	5.228	-2.902	1.312
C	3.349	-3.458	2.241
H	2.896	-3.442	3.242
H	2.794	-2.711	1.651
C	3.097	-4.800	1.604
O	3.902	-5.329	0.826
N	1.922	-5.374	1.905
H	1.289	-4.973	2.582
H	1.681	-6.265	1.495
C	6.534	-1.587	3.344
O	7.167	-0.875	2.573
N	7.101	-2.246	4.373
H	6.563	-2.787	5.033
H	8.098	-2.170	4.514
C	-8.294	1.620	2.093
N	-9.494	1.573	2.993
H	-7.439	3.554	2.527
H	-7.050	2.302	3.706
H	-7.971	0.582	1.967
H	-8.678	1.962	1.133
H	-10.221	1.007	2.545
H	-9.286	1.156	3.904
H	-9.887	2.503	3.162

2.4. Orientation of the 4th residue side chain

As shown above, mutation at the 4th residue may cause dramatic global foldedness among the peptides. Therefore, looking at the orientation of the 4th residue side chain may shed light on how the structures of **1**, **2** and **3** differ, and hence explains the big differences in stability. ROESY NMR spectra of three peptide **1-3** were inspected as depicted below.

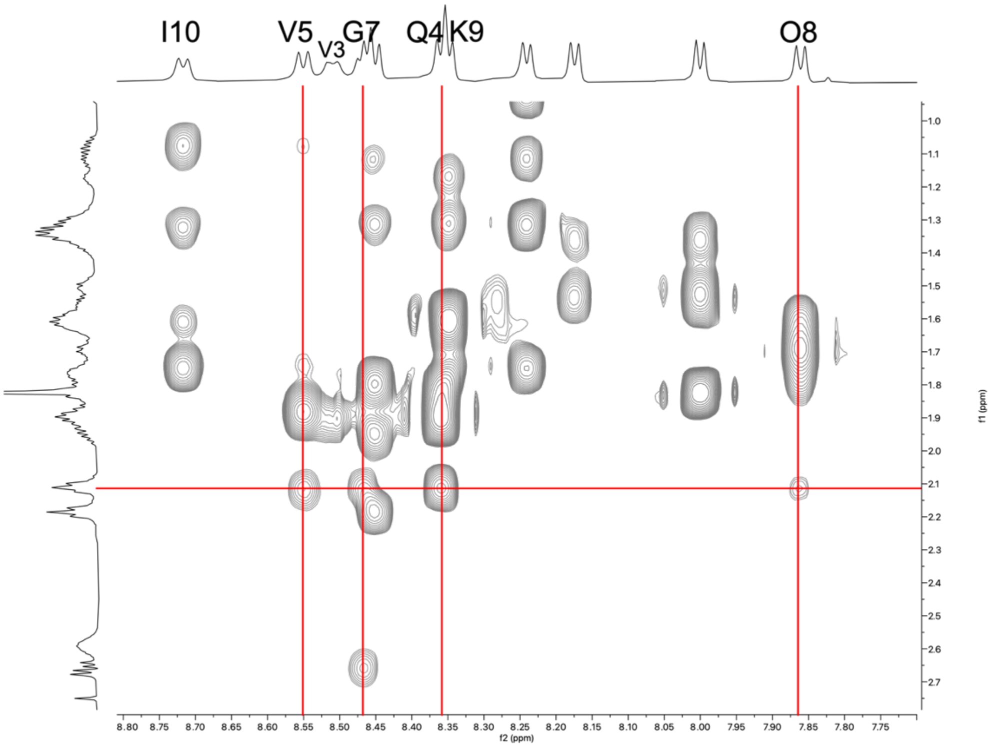

Figure S8. *A zoom-in of ROESY spectrum of **1**. Cross peaks of Q4's H_γ (marked by the horizontal line, δ = 2.12 ppm) with amide protons of V5, G7, O8 (marked by vertical lines) indicate that Q4 side chain faces towards the turn.*

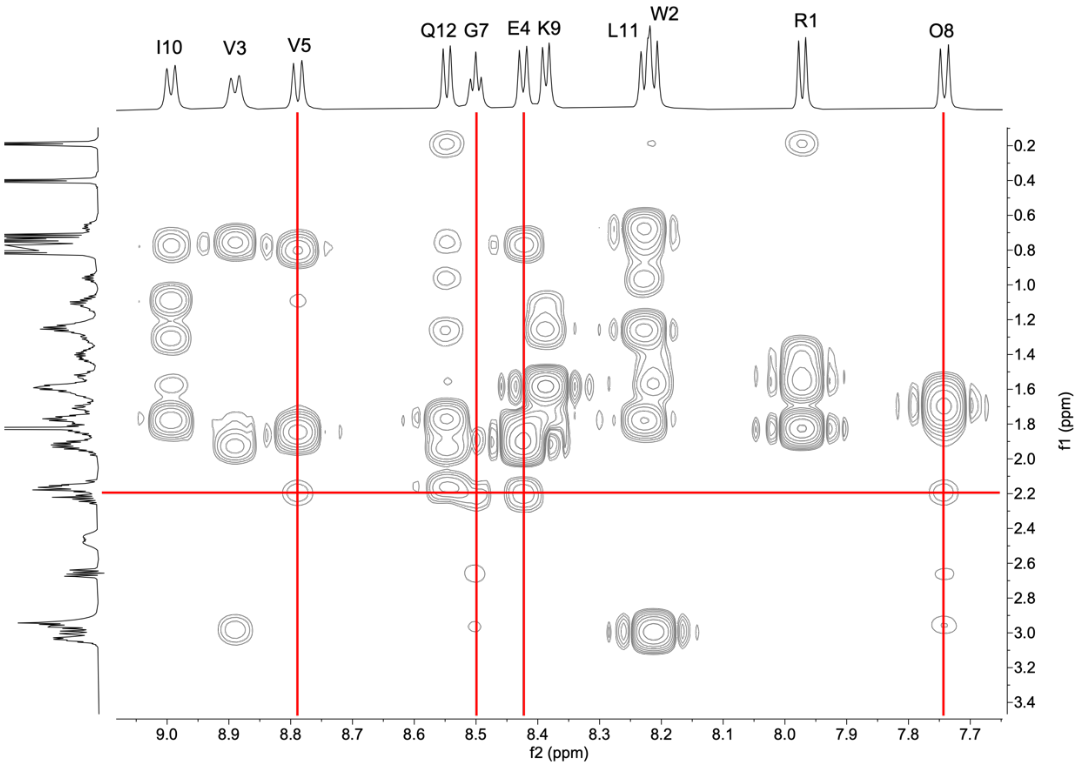

Figure S9. *A zoom-in of ROESY spectrum of **2**. Cross peaks of E4's* H_{γ} *(marked by the horizontal line, δ = 2.19 ppm) with amide protons of V5, G7, O8 (marked by vertical lines) indicate that Q4 side chain faces towards the turn.*

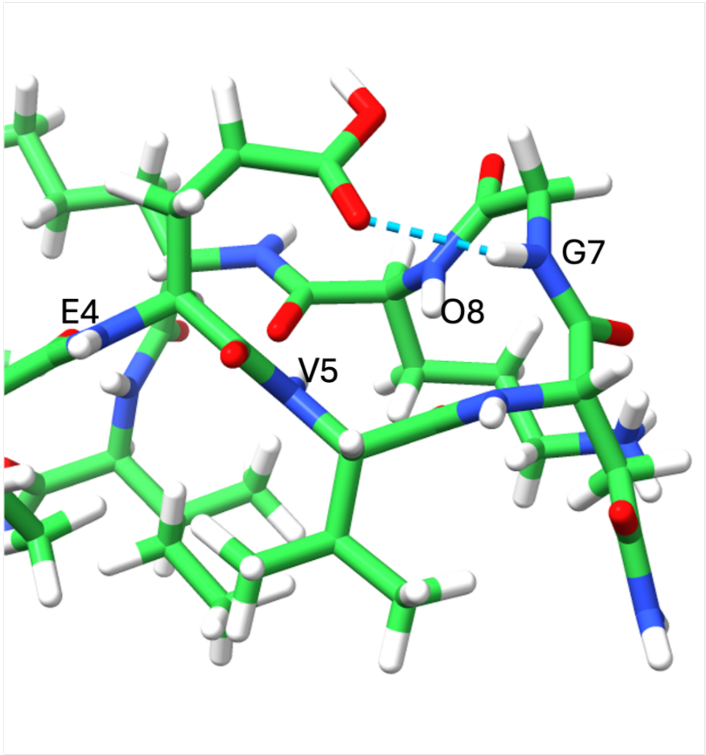

Figure S10. *Zoom-in of energy minimized structure of peptide **2**. E4's carbonyl group forms a hydrogen bond with G7 amide (highlighted by a dashed blue line).*

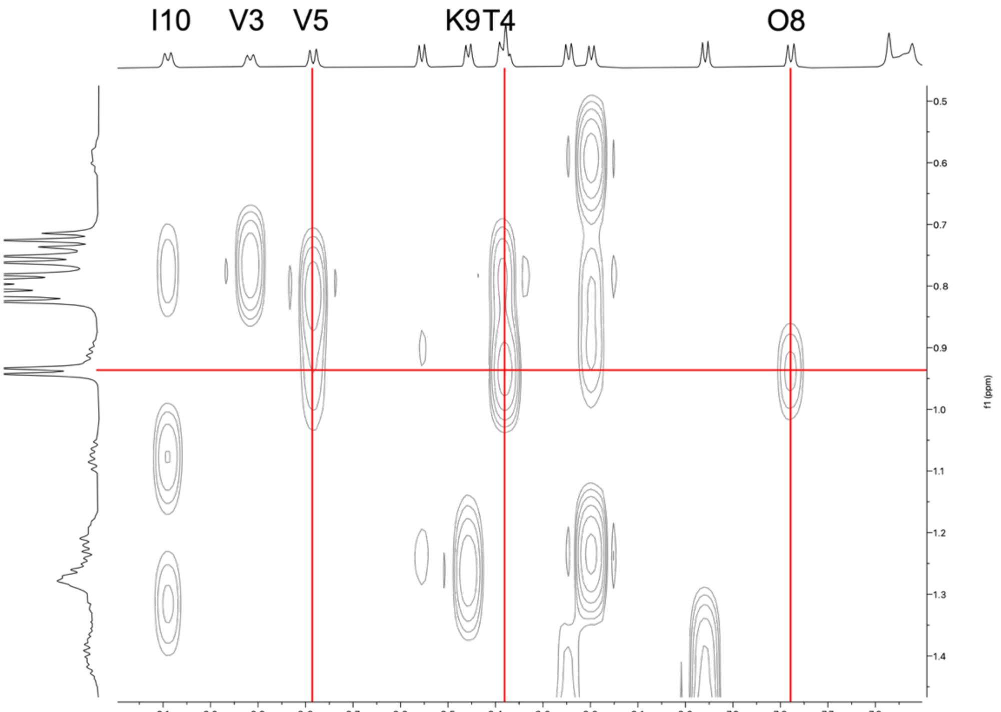

Figure S11. *A zoom-in of the ROESY spectrum of **3**. Cross peaks of T4's* H_{γ} *(marked by the horizontal line,* $\delta = 0.94$ *ppm) with amide protons of V5, O8 (marked by vertical lines) indicate that the T4 methyl group faces towards the turn and the hydroxyl group faces towards the termini.*

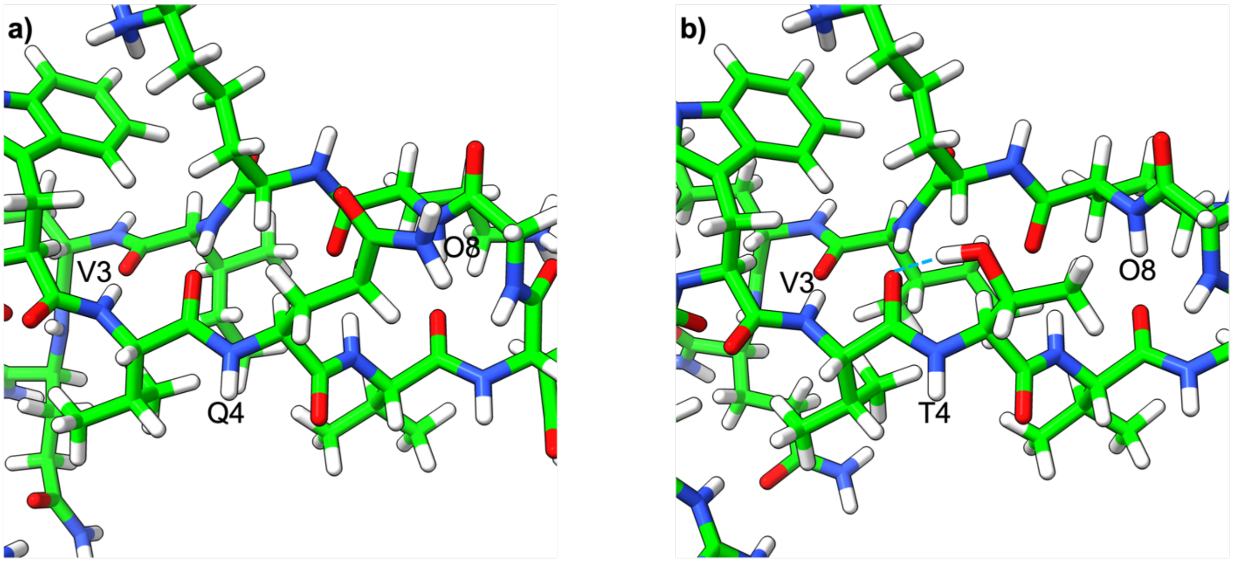

Figure S12. *Zoom-ins of energy minimized structure of a) peptide **1** and b) peptide **3**. In b), T4's hydroxyl group forms a hydrogen bond with V3 carbonyl group (highlighted by a dashed blue line).*

2.5. Side chain packing between R1 and Q12

The hydrogen bonding between R1 and Q12, along with the close packing adjacent to the sidechains (V3 and I10) depicted by the modelling. Energy minimized structure of three peptide **1-3** are depicted below.

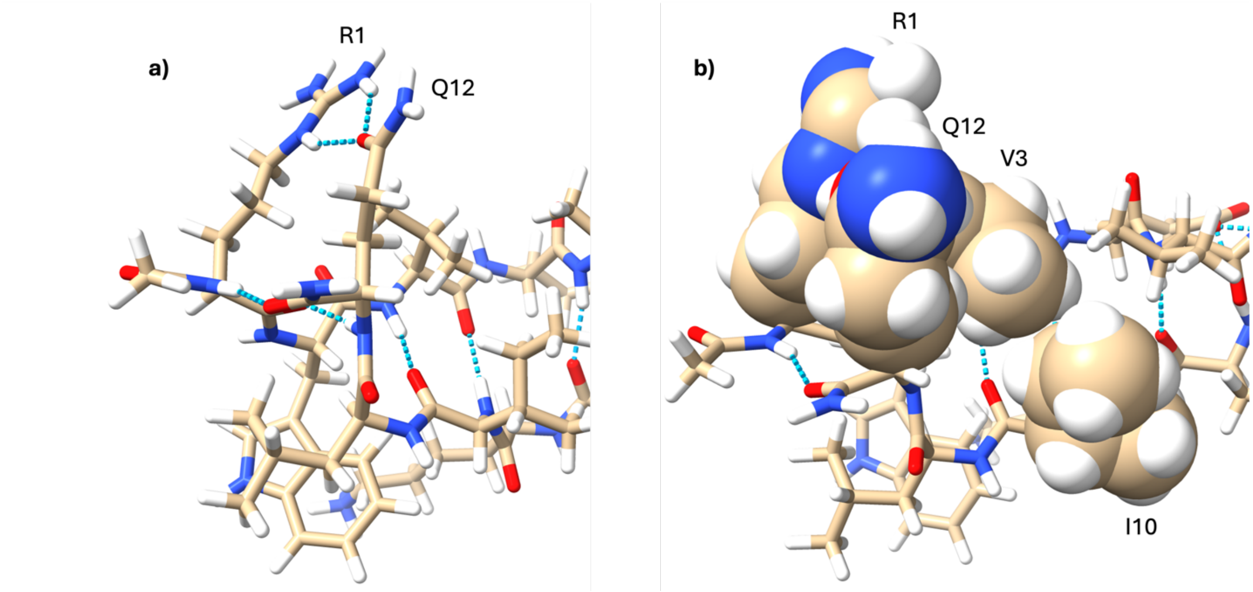

Figure S13. (a) Energy minimized structure of **1** showing the hydrogen bonding between R1 and Q12. (b) The close packing of R1 and Q12 to the side chains of V3 and I10, highlighted by CPK visuals.

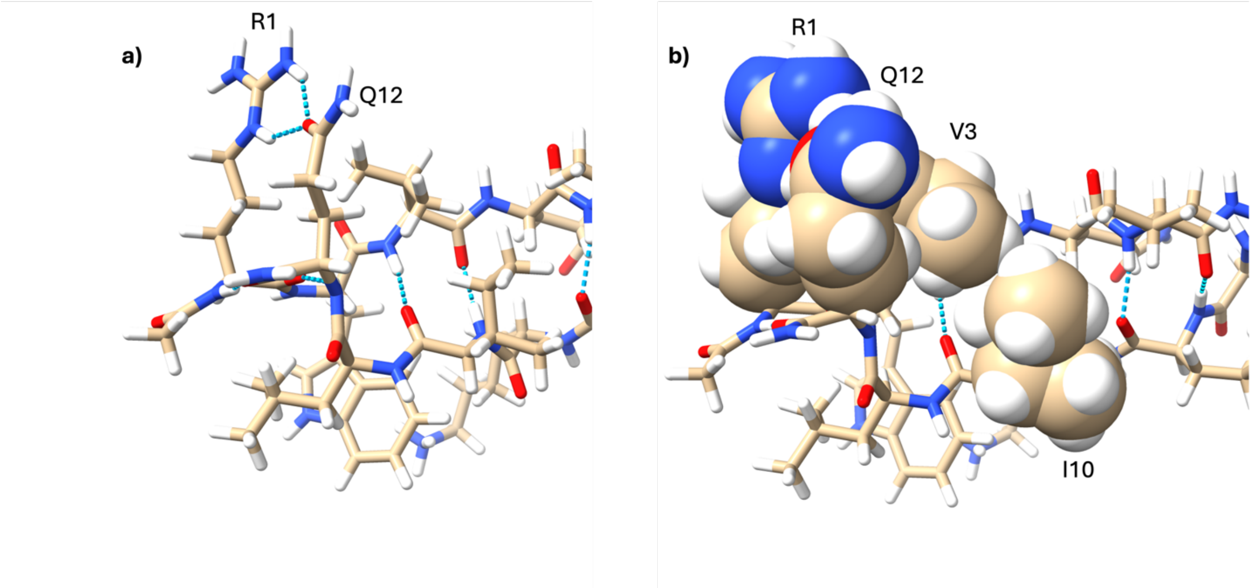

Figure S14. (a) Energy minimized structure of **2** showing the hydrogen bonding between R1 and Q12. (b) The close packing of R1 and Q12 to the side chains of V3 and I10, highlighted by CPK visuals.

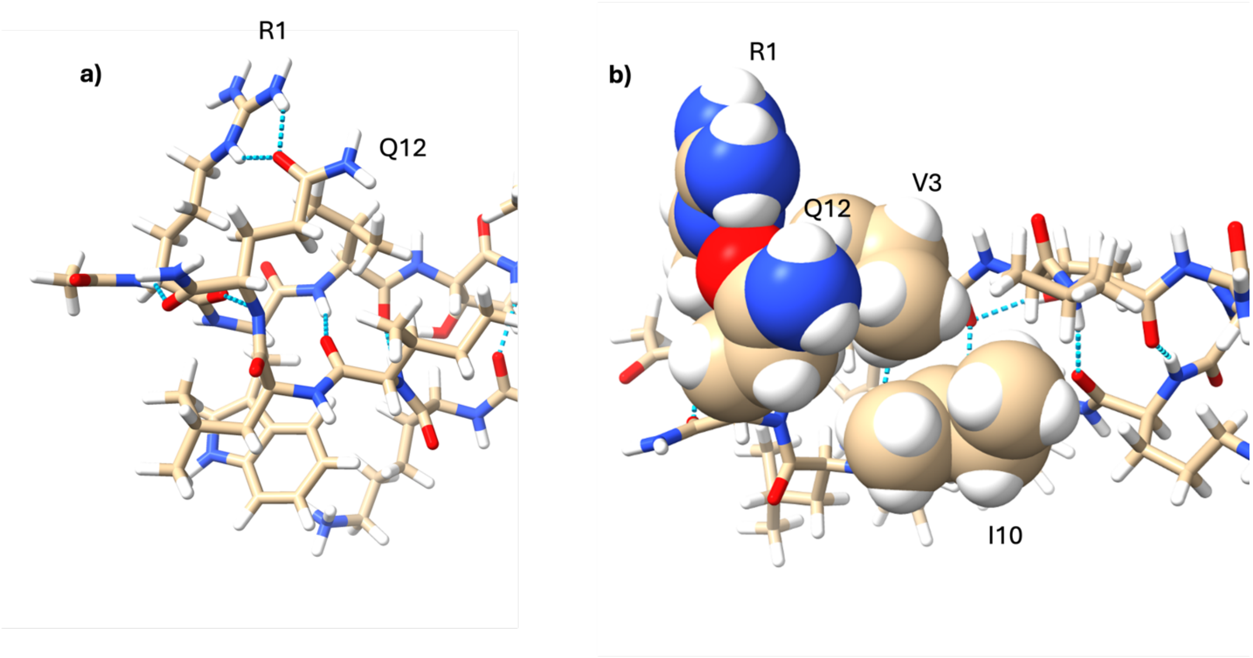

Figure S15. *(a) Energy minimized structure of* ***3*** *showing the hydrogen bonding between R1 and Q12. (b) The close packing of R1 and Q12 to the side chains of V3 and I10, highlighted by CPK visuals.*

3. VT NMR experiments

Figure S16-S26 show the VT NMR spectra of the peptides **1-11**, with each accompanied by a correspond table of backbone amide protons chemical shifts (Table S19-S29).

3.1. VT NMR data of peptide 1

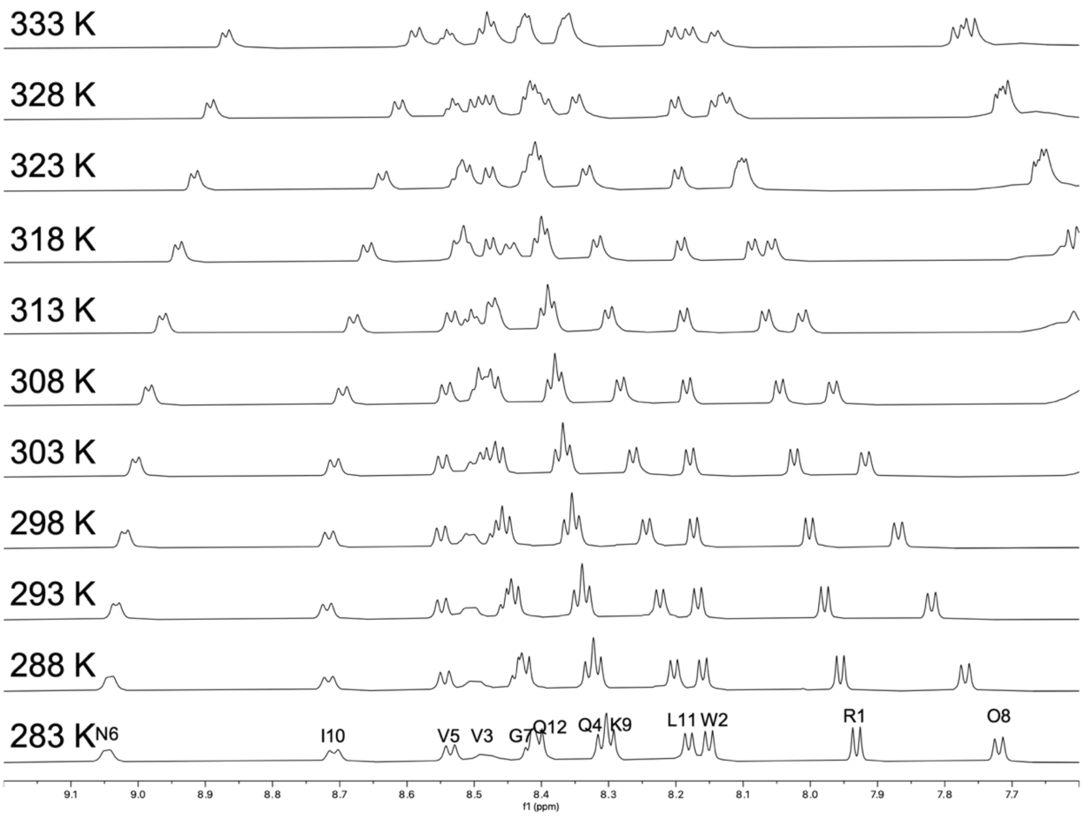

Figure S16. *Stack of* ^{1}*H NMR spectra showing the amide proton region of* ***1*** *from 10 °C (283 K) to 60 °C (333 K)*

Table S19. *Backbone amide proton chemical shift values (δ, ppm) of* ***1*** *from 283 K to 333 K*

Temperature (K)	R1	W2	V3	Q4	V5	N6	G7	O8	K9	I10	L11	Q12
283	7.937	8.157	8.478	8.316	8.542	9.041	8.415	7.726	8.303	8.715	8.186	8.399
288	7.961	8.166	8.494	8.335	8.550	9.037	8.435	7.776	8.323	8.723	8.208	8.418
293	7.984	8.173	8.501	8.352	8.555	9.028	8.452	7.826	8.340	8.726	8.229	8.434
298	8.008	8.180	8.501	8.367	8.556	9.015	8.468	7.876	8.355	8.722	8.250	8.447
303	8.030	8.185	8.491	8.380	8.554	8.999	8.482	7.925	8.369	8.715	8.269	8.458
308	8.052	8.19	8.486	8.392	8.549	8.980	8.494	7.973	8.381	8.703	8.289	8.465
313	8.073	8.195	8.470	8.402	8.541	8.959	8.505	8.019	8.391	8.686	8.306	8.470
318	8.093	8.199	8.441	8.411	8.531	8.936	8.516	8.065	8.400	8.666	8.323	8.472
323	8.107	8.203	8.417	8.419	8.519	8.912	8.525	8.107	8.412	8.643	8.340	8.473
328	8.131	8.208	8.390	8.428	8.506	8.888	8.533	8.148	8.420	8.619	8.355	8.472
333	8.148	8.213	8.358	8.436	8.493	8.865	8.542	8.187	8.429	8.594	8.369	8.471

3.2. VT NMR data of peptide 2

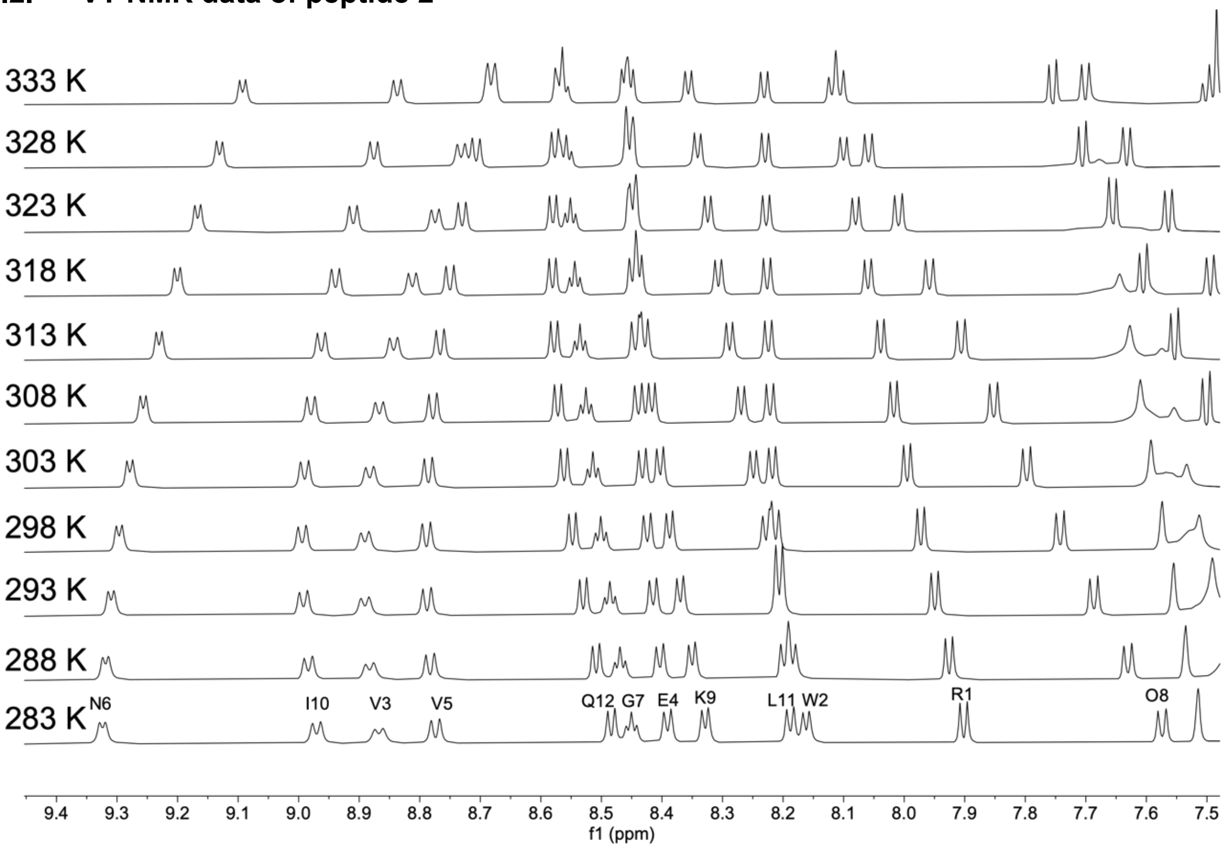

Figure S17. *Stack of ^{1}H NMR spectra showing the amide proton region of **2** from 10 °C (283 K) to 60 °C (333 K)*

Table S20. *Backbone amide proton chemical shift values (δ, ppm) of **2** from 283 K to 333 K*

Temperature (K)	R1	W2	V3	E4	V5	N6	G7	O8	K9	I10	L11	Q12
283	7.908	8.194	8.874	8.397	8.781	9.329	8.450	7.582	8.334	8.976	8.167	8.489
288	7.931	8.204	8.889	8.409	8.789	9.324	8.470	7.638	8.356	8.990	8.190	8.514
293	7.955	8.213	8.898	8.421	8.794	9.314	8.485	7.693	8.375	9.000	8.213	8.535
298	7.978	8.219	8.898	8.430	8.796	9.301	8.501	7.75	8.394	9.002	8.235	8.554
303	8.000	8.224	8.889	8.438	8.792	9.283	8.514	7.805	8.409	8.996	8.255	8.568
308	8.023	8.228	8.874	8.445	8.786	9.262	8.525	7.859	8.421	8.986	8.275	8.577
313	8.045	8.230	8.850	8.450	8.774	9.235	8.535	7.912	8.434	8.969	8.294	8.584
318	8.066	8.233	8.819	8.454	8.756	9.205	8.544	7.965	8.443	8.946	8.313	8.587
323	8.086	8.234	8.781	8.456	8.738	9.171	8.551	8.016	8.453	8.917	8.330	8.585
328	8.106	8.236	8.738	8.458	8.713	9.135	8.558	8.066	8.460	8.882	8.347	8.582
333	8.124	8.237	8.687	8.459	8.687	9.097	8.565	8.112	8.467	8.843	8.362	8.577

3.3. VT NMR data of peptide 3

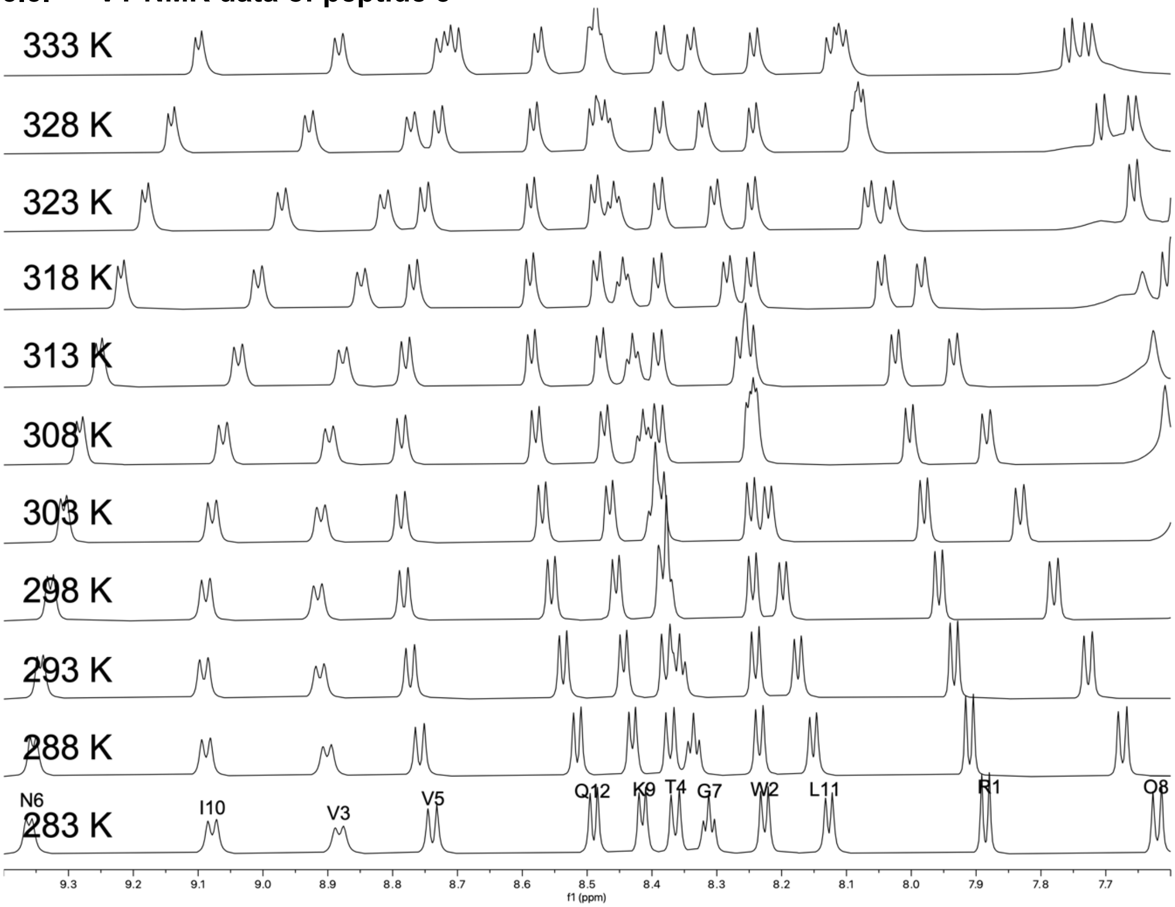

Figure S18. *Stack of ^{1}H NMR spectra showing the amide proton region of **3** from 10 °C (283 K) to 60 °C (333 K)*

Table S21. *Backbone amide proton chemical shift values (δ, ppm) of **3** from 283 K to 333 K*

Temperature (K)	R1	W2	V3	T4	V5	N6	G7	O8	K9	I10	L11	Q12
283	7.892	8.233	8.889	8.371	8.745	9.366	8.312	7.627	8.420	9.085	8.132	8.495
288	7.916	8.240	8.908	8.379	8.765	9.360	8.336	7.681	8.436	9.095	8.157	8.521
293	7.940	8.247	8.919	8.385	8.780	9.349	8.358	7.734	8.450	9.098	8.180	8.543
298	7.964	8.251	8.922	8.391	8.790	9.333	8.378	7.787	8.461	9.095	8.204	8.561
303	7.987	8.254	8.917	8.395	8.794	9.313	8.397	7.840	8.471	9.085	8.227	8.576
308	8.009	8.255	8.905	8.397	8.794	9.288	8.414	7.891	8.480	9.069	8.249	8.586
313	8.031	8.256	8.884	8.398	8.787	9.258	8.431	7.942	8.486	9.045	8.270	8.592
318	8.053	8.254	8.855	8.398	8.775	9.224	8.446	7.992	8.491	9.015	8.291	8.595
323	8.073	8.253	8.820	8.397	8.758	9.187	8.460	8.040	8.495	8.978	8.310	8.593
328	8.093	8.251	8.779	8.396	8.736	9.147	8.473	8.087	8.498	8.936	8.329	8.589
333	8.112	8.250	8.733	8.394	8.711	9.105	8.486	8.132	8.498	8.890	8.346	8.582

3.4. VT NMR data of peptide 4

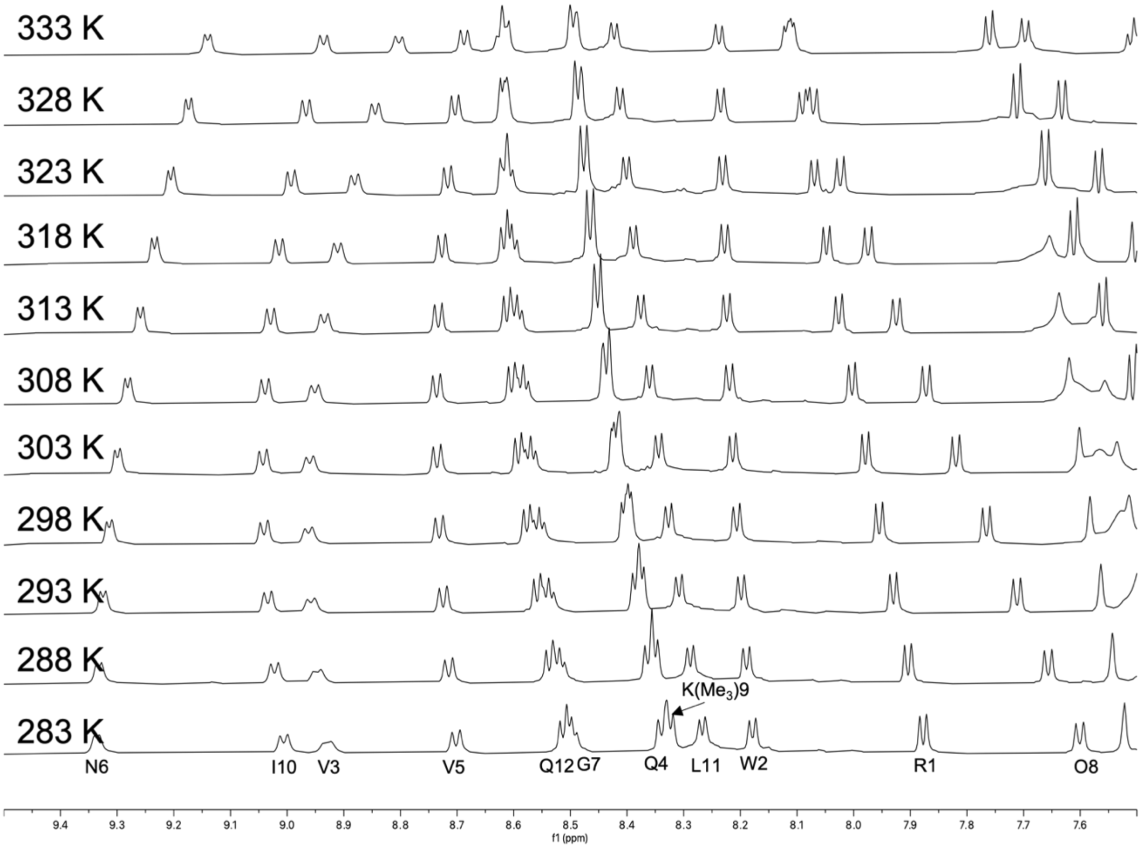

Figure S19. *Stack of* [1]*H NMR spectra showing the amide proton region of **4** from 10 °C (283 K) to 60 °C (333 K)*

Table S22*. Backbone amide proton chemical shift values (δ, ppm) of **4** from 283 K to 333 K*

Temperature (K)	R1	W2	V3	Q4	V5	N6	G7	O8	K(Me_3)9	I10	L11	Q12
283	7.883	8.185	8.936	8.345	8.709	9.341	8.489	7.608	8.319	9.013	8.272	8.518
288	7.910	8.196	8.953	8.369	8.721	9.337	8.510	7.663	8.346	9.029	8.294	8.543
293	7.936	8.205	8.964	8.391	8.731	9.330	8.530	7.718	8.370	9.041	8.314	8.565
298	7.961	8.213	8.969	8.410	8.738	9.319	8.547	7.772	8.392	9.048	8.332	8.583
303	7.985	8.220	8.967	8.429	8.742	9.305	8.561	7.826	8.413	9.049	8.350	8.598
308	8.009	8.225	8.958	8.445	8.743	9.286	8.574	7.879	8.431	9.046	8.366	8.609
313	8.032	8.230	8.941	8.458	8.740	9.264	8.585	7.931	8.447	9.036	8.381	8.618
318	8.054	8.234	8.918	8.471	8.733	9.239	8.595	7.980	8.460	9.021	8.395	8.623
323	8.075	8.238	8.887	8.483	8.723	9.211	8.602	8.030	8.471	9.000	8.407	8.624
328	8.096	8.241	8.851	8.492	8.710	9.179	8.608	8.077	8.479	8.973	8.418	8.624
333	8.123	8.244	8.809	8.500	8.694	9.145	8.610	8.124	8.488	8.942	8.428	8.620

3.5. VT NMR data of peptide 5

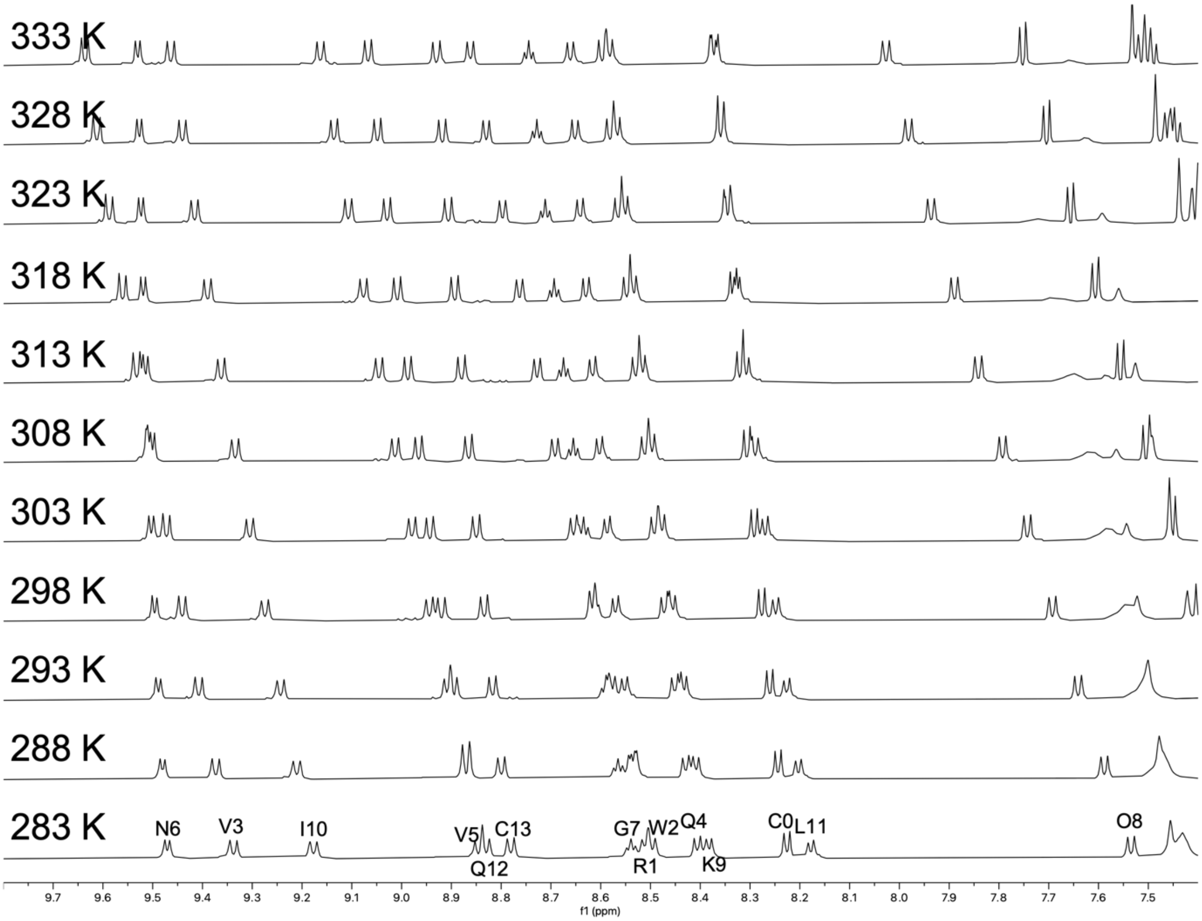

Figure S20. *Stack of ^{1}H NMR spectra showing the amide proton region of **5** from 10 °C (283 K) to 60 °C (333 K)*

Table S23. *Backbone amide proton chemical shift values (δ, ppm) of **5** from 283 K to 333 K†*

Temperature (K)	R1	W2	V3	Q4	V5	N6	G7	O8	K9	I10	L11	Q12
283	8.506	8.517	9.345	8.412	8.853	9.475	8.539	7.541	8.388	9.184	8.183	8.838
288	8.544	8.539	9.380	8.436	8.878	9.485	8.565	7.595	8.414	9.218	8.208	8.878
293	8.584	8.558	9.415	8.458	8.903	9.493	8.589	7.648	8.439	9.250	8.232	8.914
298	8.623	8.576	9.448	8.479	8.927	9.501	8.612	7.700	8.462	9.282	8.254	8.951
303	8.661	8.593	9.479	8.499	8.950	9.508	8.634	7.750	8.483	9.312	8.275	8.986
308	8.698	8.608	9.510	8.518	8.973	9.514	8.655	7.800	8.505	9.341	8.298	9.020
313	8.734	8.622	9.540	8.536	8.994	9.519	8.675	7.848	8.523	9.369	8.314	9.052
318	8.770	8.635	9.568	8.554	9.016	9.524	8.694	7.896	8.541	9.397	8.332	9.084
323	8.804	8.647	9.594	8.571	9.036	9.528	8.712	7.943	8.558	9.423	8.348	9.114
328	8.836	8.657	9.619	8.588	9.055	9.532	8.728	7.989	8.575	9.447	8.365	9.142
333	8.868	8.667	9.643	8.604	9.074	9.535	8.745	8.034	8.588	9.471	8.380	9.170

† C0 and C13 are not included.

3.6. VT NMR data of peptide 6

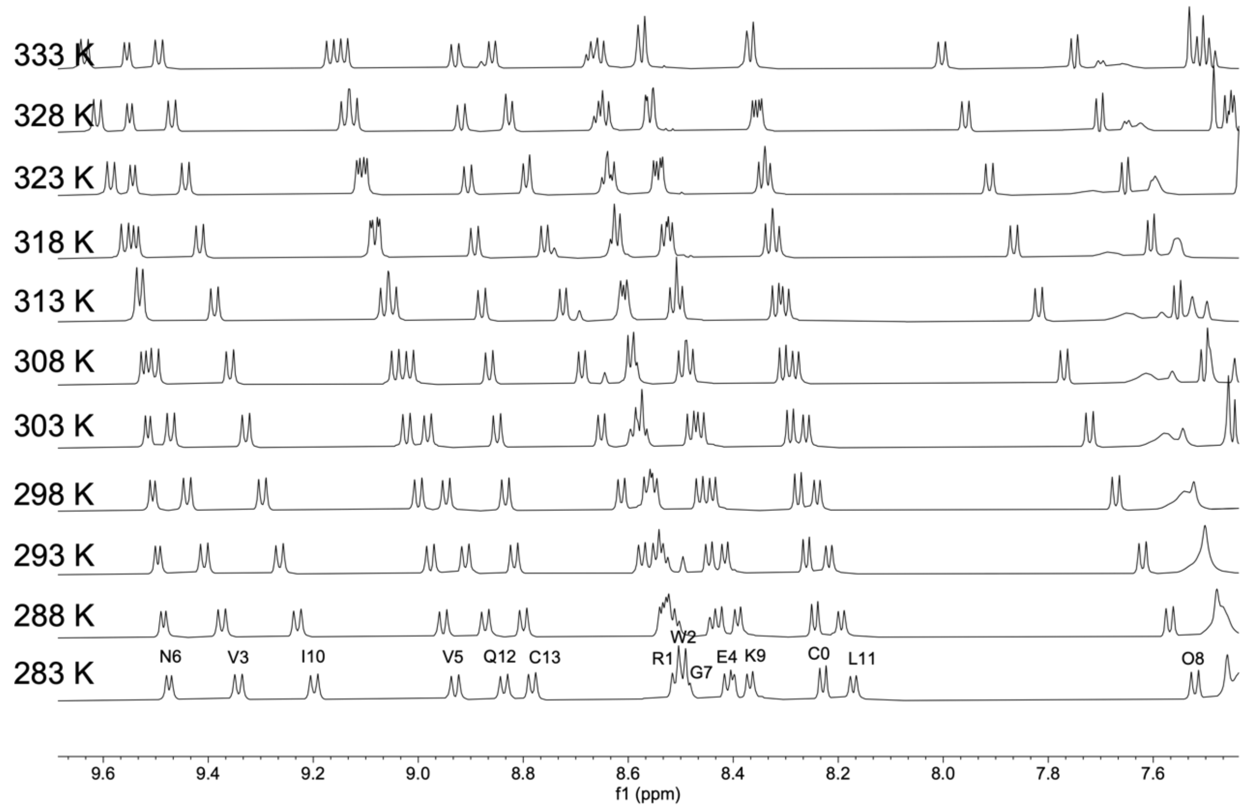

Figure S21. *Stack of ^{1}H NMR spectra showing the amide proton region of **6** from 10 °C (283 K) to 60 °C (333 K)*

Table S24. *Backbone amide proton chemical shift values (δ, ppm) of **6** from 283 K to 333 K†*

Temperature (K)	R1	W2	V3	E4	V5	N6	G7	O8	K9	I10	L11	Q12
283	8.517	8.504	9.349	8.417	8.936	9.479	8.491	7.529	8.376	9.205	8.178	8.844
288	8.541	8.530	9.381	8.435	8.961	9.491	8.512	7.576	8.398	9.237	8.200	8.879
293	8.580	8.552	9.415	8.453	8.985	9.501	8.534	7.628	8.422	9.272	8.224	8.918
298	8.620	8.570	9.447	8.471	9.007	9.510	8.554	7.678	8.444	9.303	8.247	8.953
303	8.658	8.585	9.478	8.488	9.030	9.520	8.574	7.728	8.468	9.335	8.267	8.988
308	8.696	8.601	9.509	8.504	9.051	9.528	8.595	7.778	8.491	9.366	8.288	9.022
313	8.731	8.615	9.537	8.522	9.071	9.537	8.610	7.826	8.508	9.395	8.306	9.057
318	8.766	8.627	9.565	8.537	9.092	9.543	8.627	7.872	8.524	9.423	8.326	9.079
323	8.802	8.640	9.592	8.553	9.118	9.549	8.640	7.920	8.545	9.450	8.339	9.105
328	8.834	8.649	9.618	8.568	9.147	9.556	8.656	7.964	8.566	9.476	8.350	9.132
333	8.865	8.660	9.642	8.581	9.175	9.560	8.671	8.010	8.581	9.502	8.363	9.147

† C0 and C13 are not included.

3.7. VT NMR data of peptide 7

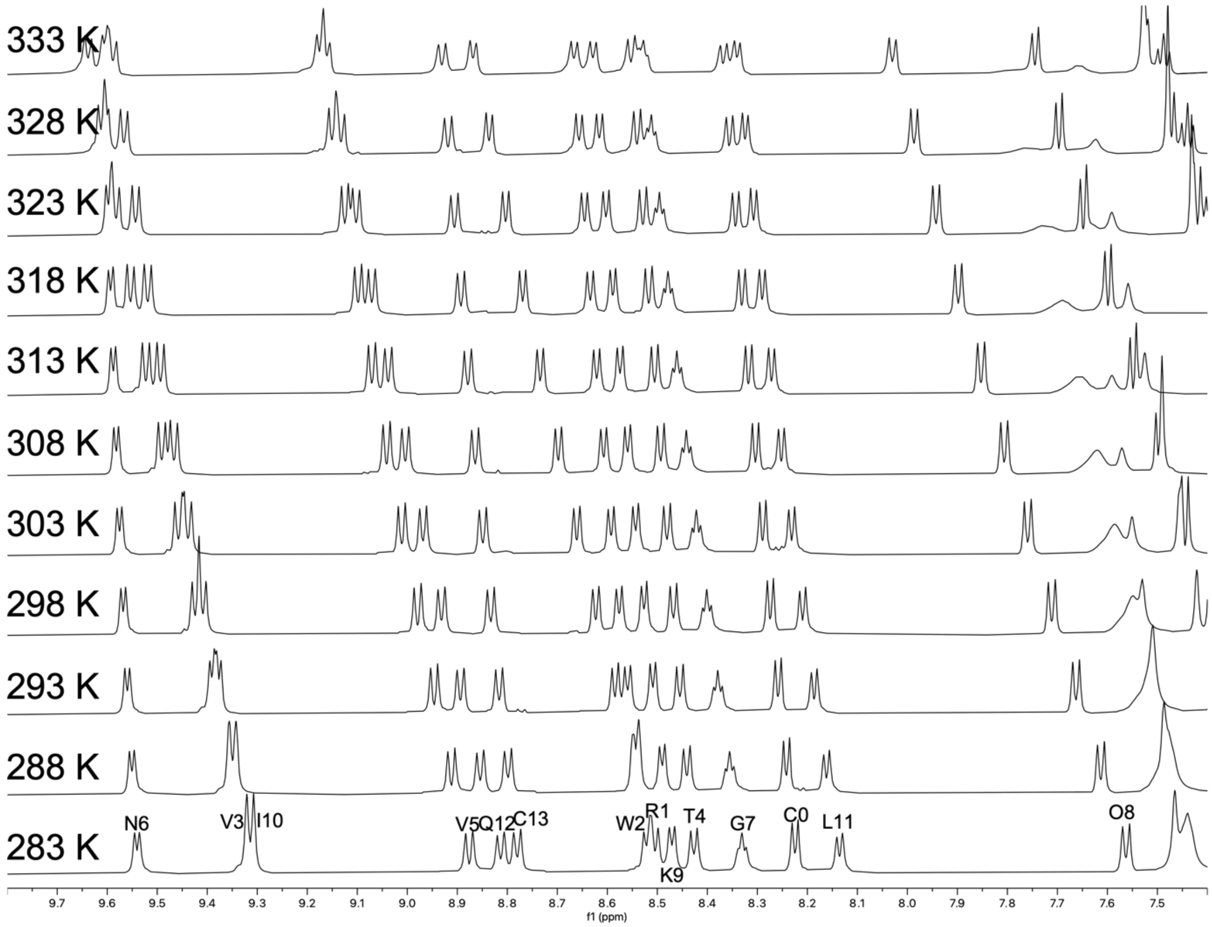

Figure S22. *Stack of ^{1}H NMR spectra showing the amide proton region of **7** from 10 °C (283 K) to 60 °C (333 K)*

Table S25. *Backbone amide proton chemical shift values (δ, ppm) of **7** from 283 K to 333 K†*

Temperature (K)	R1	W2	V3	T4	V5	N6	G7	O8	K9	I10	L11	Q12
283	8.511	8.527	9.322	8.433	8.884	9.545	8.331	7.570	8.476	9.322	8.142	8.820
288	8.550	8.547	9.358	8.448	8.920	9.556	8.356	7.620	8.496	9.355	8.168	8.861
293	8.591	8.565	9.395	8.461	8.954	9.564	8.379	7.670	8.514	9.382	8.193	8.901
298	8.629	8.582	9.430	8.474	8.987	9.573	8.401	7.718	8.532	9.417	8.215	8.938
303	8.667	8.599	9.464	8.488	9.018	9.580	8.422	7.767	8.549	9.446	8.237	8.975
308	8.704	8.614	9.498	8.500	9.048	9.587	8.442	7.814	8.565	9.474	8.258	9.011
313	8.740	8.628	9.530	8.512	9.077	9.593	8.461	7.860	8.580	9.500	8.278	9.045
318	8.775	8.640	9.560	8.524	9.105	9.598	8.479	7.905	8.595	9.526	8.296	9.078
323	8.810	8.652	9.590	8.535	9.132	9.603	8.495	7.950	8.609	9.550	8.314	9.109
328	8.842	8.663	9.617	8.547	9.157	9.606	8.512	7.993	8.621	9.574	8.330	9.139
333	8.874	8.672	9.645	8.559	9.180	9.610	8.528	8.037	8.635	9.596	8.346	9.167

† C0 and C13 are not included.

3.8. VT NMR data of peptide 8

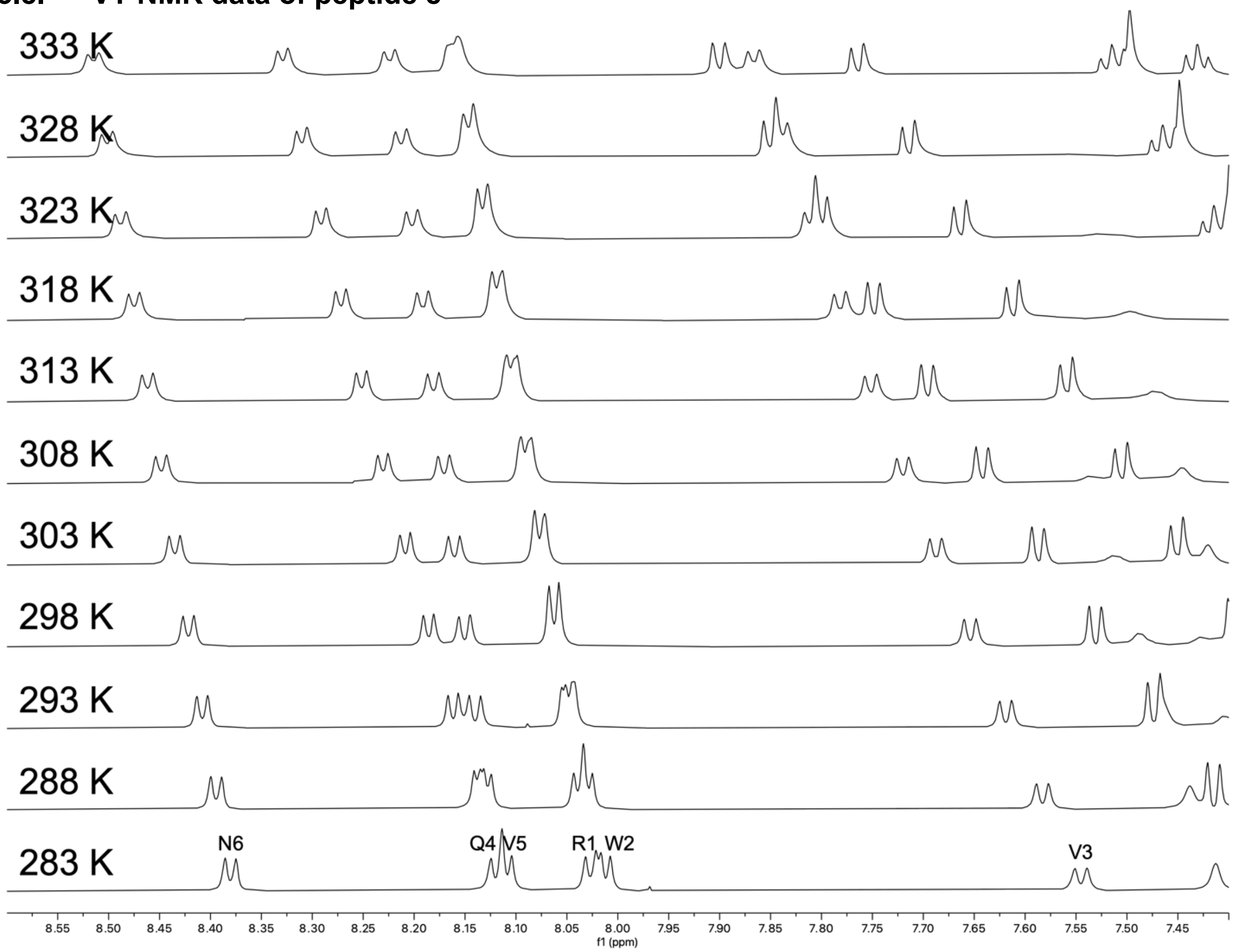

Figure S23. *Stack of* ^{1}*H NMR spectra showing the amide proton region of* ***8*** *from 10 °C (283 K) to 60 °C (333 K)*

Table S26. *Backbone amide proton chemical shift values (δ, ppm) of* ***8*** *from 283 K to 333 K*

Temperature (K)	R1	W2	V3	Q4	V5	N6
283	8.016	8.031	7.551	8.124	8.113	8.385
288	8.034	8.043	7.589	8.141	8.132	8.400
293	8.051	8.056	7.625	8.167	8.146	8.413
298	8.068	8.068	7.660	8.191	8.156	8.427
303	8.082	8.082	7.694	8.214	8.166	8.441
308	8.095	8.095	7.726	8.236	8.177	8.454
313	8.109	8.109	7.758	8.257	8.187	8.468
318	8.123	8.123	7.788	8.277	8.197	8.481
323	8.138	8.138	7.817	8.297	8.208	8.494
328	8.152	8.152	7.845	8.316	8.219	8.507
333	8.168	8.168	7.872	8.335	8.230	8.521

3.9. VT NMR data of peptide 9

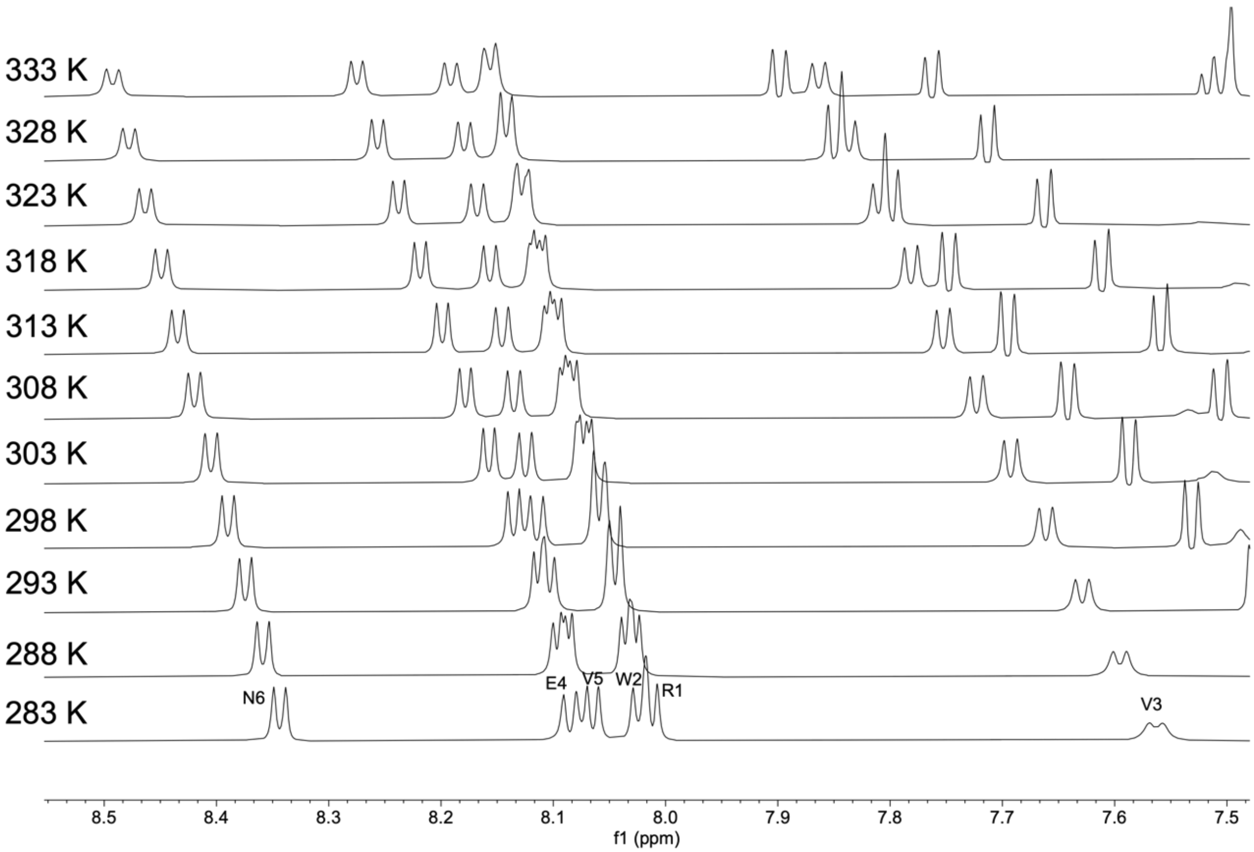

Figure S24. *Stack of ^{1}H NMR spectra showing the amide proton region of* ***9*** *from 10 °C (283 K) to 60 °C (333 K)*

Table S27. *Backbone amide proton chemical shift values (δ, ppm) of* ***9*** *from 283 K to 333 K*

Temperature (K)	R1	W2	V3	E4	V5	N6
283	8.018	8.030	7.570	8.069	8.091	8.349
288	8.031	8.039	7.601	8.093	8.100	8.364
293	8.040	8.050	7.635	8.118	8.113	8.379
298	8.055	8.064	7.667	8.141	8.121	8.395
303	8.070	8.075	7.699	8.162	8.131	8.410
308	8.085	8.085	7.728	8.183	8.141	8.425
313	8.099	8.099	7.758	8.204	8.152	8.440
318	8.112	8.112	7.787	8.224	8.162	8.454
323	8.122	8.122	7.815	8.243	8.174	8.469
328	8.137	8.137	7.843	8.262	8.185	8.484
333	8.152	8.151	7.869	8.280	8.197	8.498

3.10. VT NMR data of peptide 10

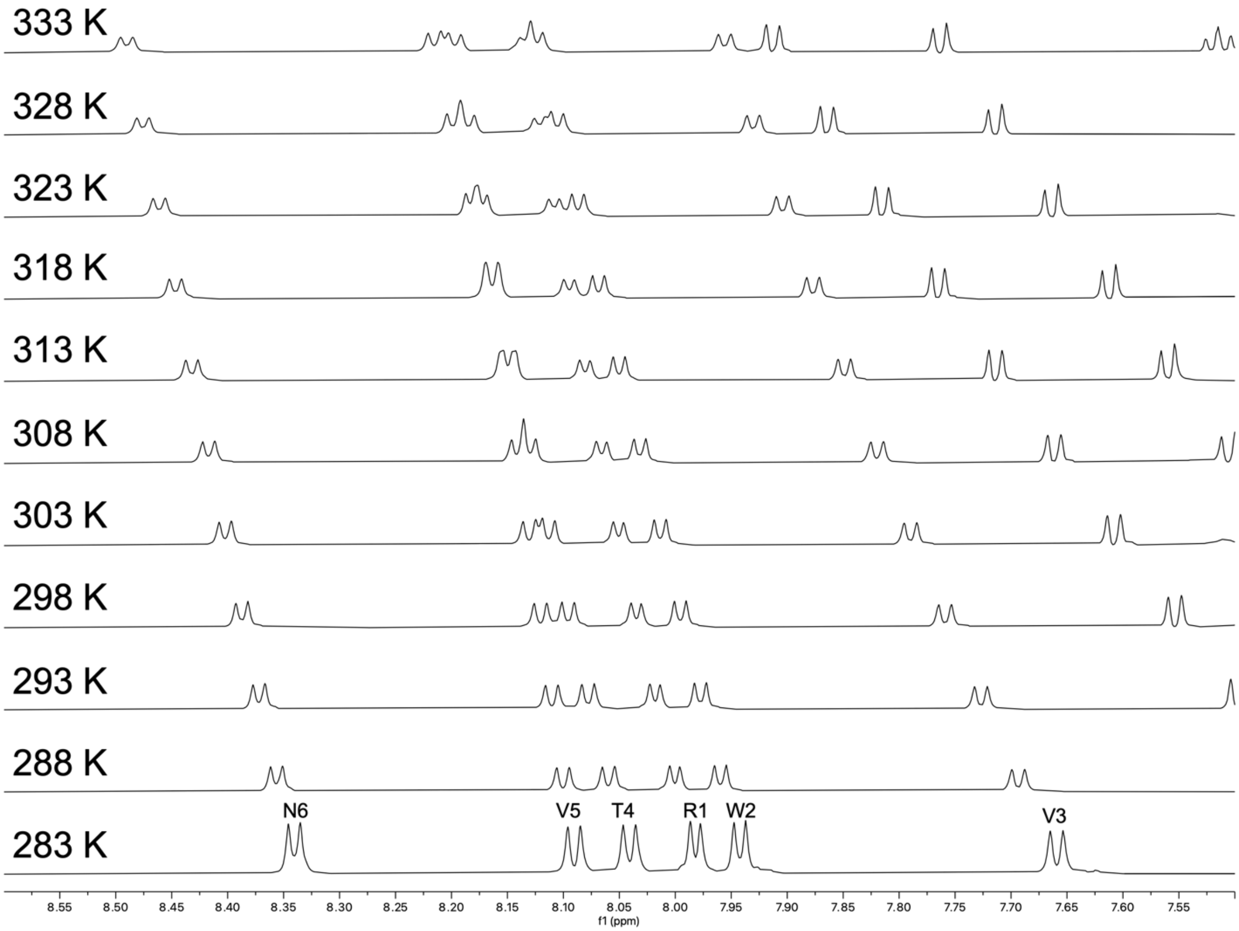

Figure S25. *Stack of ^{1}H NMR spectra showing the amide proton region of **10** from 10 °C (283 K) to 60 °C (333 K)*

Table S28. *Backbone amide proton chemical shift values (δ, ppm) of **10** from 283 K to 333 K*

Temperature (K)	R1	W2	V3	T4	V5	N6
283	7.987	7.948	7.665	8.046	8.096	8.346
288	8.005	7.965	7.699	8.065	8.106	8.361
293	8.023	7.983	7.732	8.084	8.116	8.378
298	8.040	8.001	7.765	8.102	8.127	8.393
303	8.056	8.018	7.795	8.119	8.136	8.407
308	8.070	8.037	7.825	8.136	8.147	8.423
313	8.086	8.056	7.855	8.154	8.154	8.437
318	8.100	8.074	7.883	8.170	8.169	8.453
323	8.113	8.093	7.909	8.187	8.177	8.467
328	8.126	8.111	7.936	8.204	8.192	8.482
333	8.138	8.129	7.961	8.221	8.203	8.496

3.11. VT NMR data of peptide 11

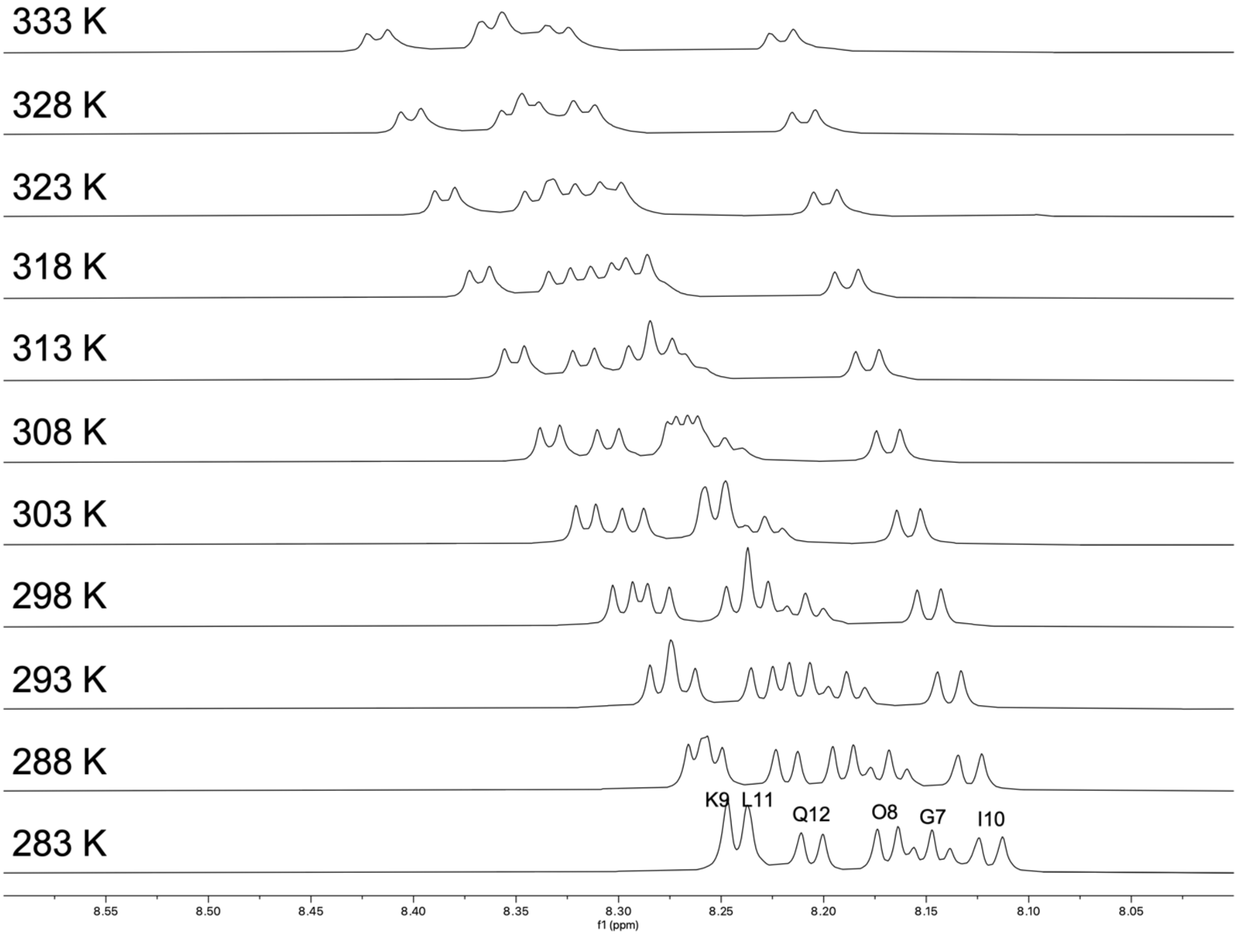

Figure S26. *Stack of ^{1}H NMR spectra showing the amide proton region of **11** from 10 °C (283 K) to 60 °C (333 K)*

Table S29. *Backbone amide proton chemical shift values (δ, ppm) of **11** from 283 K to 333 K*

Temperature (K)	G7	O8	K9	I10	L11	Q12
283	8.147	8.174	8.247	8.124	8.247	8.211
288	8.168	8.195	8.266	8.134	8.260	8.223
293	8.189	8.217	8.285	8.144	8.275	8.235
298	8.209	8.237	8.303	8.154	8.286	8.247
303	8.229	8.260	8.321	8.164	8.298	8.260
308	8.248	8.272	8.339	8.174	8.311	8.277
313	8.268	8.285	8.356	8.184	8.323	8.295
318	8.286	8.296	8.373	8.195	8.334	8.314
323	8.306	8.314	8.391	8.205	8.347	8.328
328	8.326	8.332	8.409	8.215	8.359	8.343
333	8.346	8.350	8.427	8.225	8.372	8.357

4. Data fitting

4.1. Error analysis

Generally, the error in chemical shift is ± 0.005 ppm, and the error in temperature is ± 0.1 K. These values correspond to ± 2-3% fraction folded (*f*) uncertainties. Furthermore, by performing error propagation analysis (see Appendix I), errors of Δ*G* can be demonstrated as below (Figure S27):

a) Maximum error in Δ*G* is greater than 33% if *f* > 0.9 or *f* < 0.1.
b) Maximum error in Δ*G* ranges from 14% to 33% if 0.1 < *f* < 0.3 or 0.7 < *f* < 0.9.
c) Maximum error in Δ*G* ranges from 12% to 14% if 0.3 < *f* < 0.7.

In this study, any data points with *f* > 0.90 (Δ*G* > 5444 J/mol) or *f* < 0.10 (Δ*G* < -5444 J/mol) were ignored when fitting data.

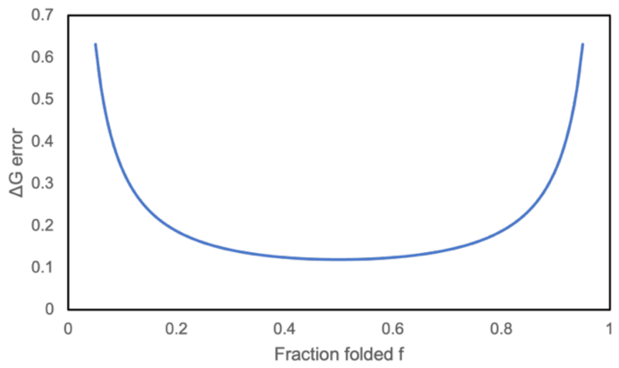

Figure S27. *Maximum relative error of ΔG as a function of fraction folded f, ranging from 0.05 < f < 0.95.*

4.2. Thermodynamic data of unfolding by glycine splitting

Glycine splitting serves as a global metric for assessing the stability of β-hairpins. Positioned within the type I/I' β-turn, the two diastereotopic α-protons of G7 exhibit sensitivity to the dynamics of folding and unfolding inherent in the antiparallel β-fold structure. Utilizing equation **(1)**, it is possible to ascertain the fraction folded *f* of the hairpin at various temperature as depicted below in Figure S28.

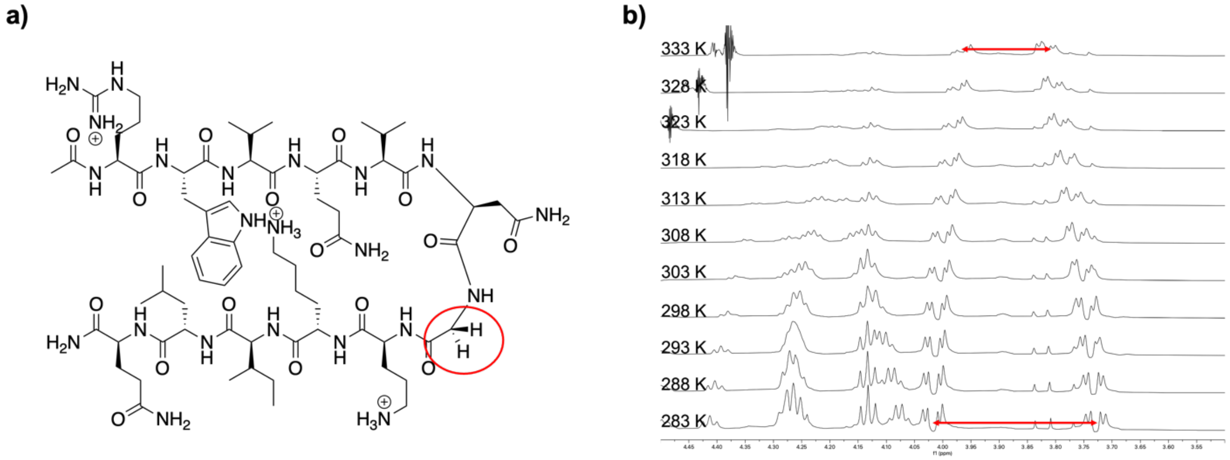

Figure S28. *a) Structure of* ***1****, G7's two diastereotopic protons are shown in the red circle*

b) Representative data showing the G7 splitting (red arrows) as a function of temperature

The fraction folded f can be subsequentially converted to ΔG_u:

$$\Delta G_u(T) = -RT\ln((1\text{-}f)/f) \qquad \textbf{(2)}$$

$\Delta G_u(T)$ was then fitted to Gibbs-Helmholtz equation[7] using iterative least squares method in MATLAB R2022a:

$$\Delta G_u(T) = (\Delta H^{\circ}_{u,298K} + \Delta C^{\circ}_{p,u}(T\text{-}298) - T(\Delta S^{\circ}_{u,298K} + \Delta C^{\circ}_{p,u}\ln(T/298)) \qquad \textbf{(3)}$$

The temperature where the peptide expresses the maximum stability $\Delta G_{u,max}$ is called T_{max}, which is derived by taking the first derivative of equation **(3)** with respect to T:

$$\frac{\partial \Delta G_u(T)}{\partial T} = 0 \Leftrightarrow T_{max} = 298 \times e^{-\frac{\Delta S^{\circ}_{u,298K}}{\Delta C^{\circ}_{p,u}}} \qquad \textbf{(4)}$$

Fitting the glycine splitting data from peptide **1-4** to equation **(3)** was carried out (Figure S29). Table S30 represent the fraction folded of **1** as a function of T, Table S31 shows the results obtained from equation **(2)**, and Table S32 summarizes the coefficients from the fitting from data from Table S31 to equation **(3)**.

Table S30. *Fraction folded f of* ***1-4*** *as a function of temperature.*

Temperature (K)	1	2	3	4
283	0.555	0.802	0.836	0.801
288	0.545	0.800	0.823	0.800
293	0.533	0.792	0.807	0.774
298	0.508	0.773	0.780	0.766
303	0.487	0.757	0.753	0.748
308	0.461	0.739	0.722	0.726
313	0.427	0.713	0.690	0.699
318	0.396	0.676	0.653	0.669
323	0.365	0.642	0.613	0.647
328	0.324	0.604	0.570	0.594
333	0.290	0.556	0.520	0.546

Table S31. *Unfolding free energy ($\Delta G_u(T)$, J mol^{-1})† of **1-4** as a function of temperature.*

Temperature (K)	1	2	3	4
283	519.5	3295.4	3837.2	3279.8
288	435.2	3323.4	3678.3	3325.5
293	321.5	3253.9	3477.6	2994.3
298	79.5	3036.4	3134.8	2939.7
303	-135.4	2865.2	2812.7	2742.9
308	-395.7	2669.7	2442.8	2494.2
313	-764.8	2362.5	2079.7	2197.3
318	-1116.8	1949.4	1669.7	1865
323	-1489.0	1571.6	1239.4	1628.7
328	-2000.0	1149.9	765.6	1036.3
333	-2478.3	626.7	222.3	509.9

† From $\Delta G_u(T) = -RT\ln((1-f)/f)$

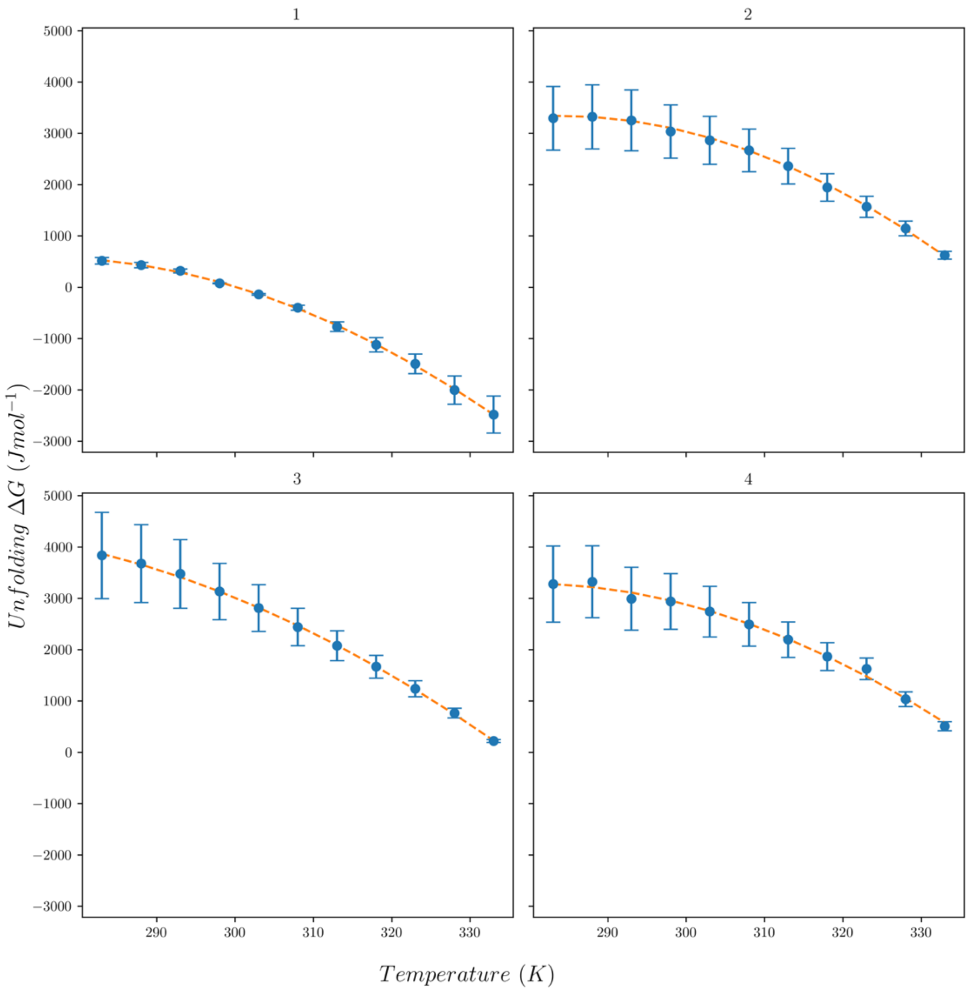

Figure S29. *Fitting (dashed lines) of $\Delta G_u(T)$ of **1-4** to equation **(3)** by measuring glycine splitting. The error bars are obtained from error propagation as described in Section 4.1.*

__Table S32.__ Unfolding thermodynamic parameters of __1-4__ from fitting $\Delta G_u(T)$ to equation __(3)__ by glycine splitting. Errors are reported in parentheses.

	**1**	**2**	**3**	**4**
$\Delta C^{\circ}_{p,u}$ (J/mol·K)‡	554.3 (43.8)	677.4 (156.4)	414.8 (24.6)	543.55 (27.1)
$\Delta H^{\circ}_{u,298K}$ (kJ/mol)‡	12.76 (0.51)	12.95 (1.83)	20.88 (0.29)	13.64 (1.78)
$\Delta S^{\circ}_{u,298K}$ (J/mol·K)‡	42.47 (1.73)	33.03 (6.16)	59.55 (0.97)	35.03 (6.01)
T_{max} (K)§	276 (2)	284 (4)	258 (2)	279 (3)

‡ Errors are obtained from the fitting.
§ Values are calculated from equation **(4)**, and the errors abide by propagation of uncertainties (see Appendix I).

4.3. Residue-wise thermodynamics fitting

4.3.1. Visualization of VT NMR data

Visualization of the tabulated NMR shifts of each mainchain amide of peptides **1**-**4** (and their corresponding macrocyclic peptides and half-peptides), as a function of temperature, are shown below (Figures S30-S33). To obtain a meaningful $\Delta G_u(T)$ at each residue, the chemical shift of the amide proton must be in the range defined by the cyclic and half-peptide references. For example, for N6: $\delta_{\mathbf{5}}^{N6} < \delta_{\mathbf{1}}^{N6} < \delta_{\mathbf{2}}^{N6}$ in the Figures S30-S33. In contrast, for the residue R1 it was always observed that: $\delta_{\mathbf{1}}^{R1} < \delta_{\mathbf{5}}^{R1} < \delta_{\mathbf{2}}^{R1}$. This yields a negative fraction folded f, which results in an undefined $\Delta G_u(T)$ (Equation **(2)**). This will be discussed more in Section 4.3.2.

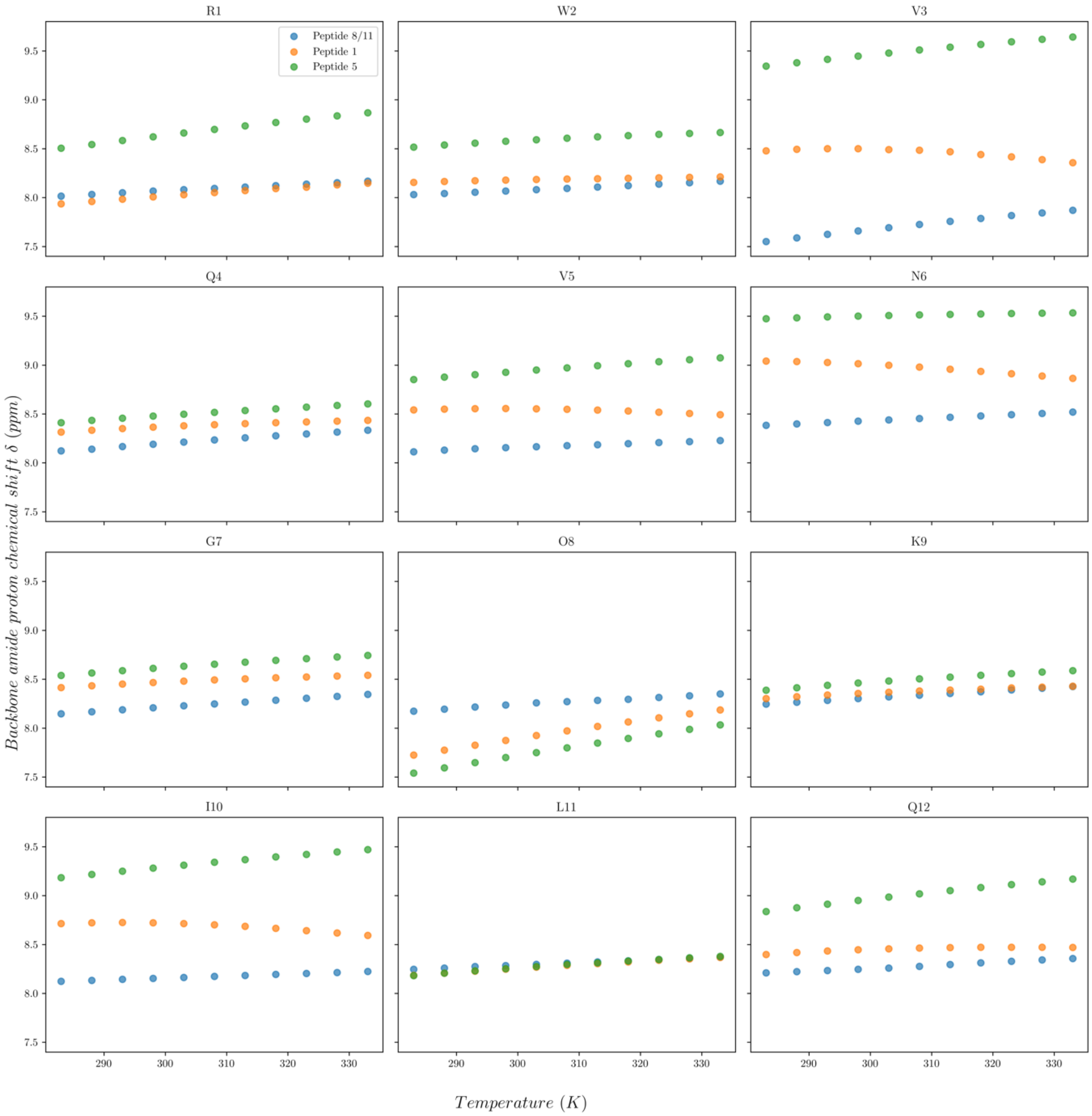

Figure S30. *Backbone amide proton chemical shift values (δ, ppm) of peptide **1**, **5**, **8** and **11**.*

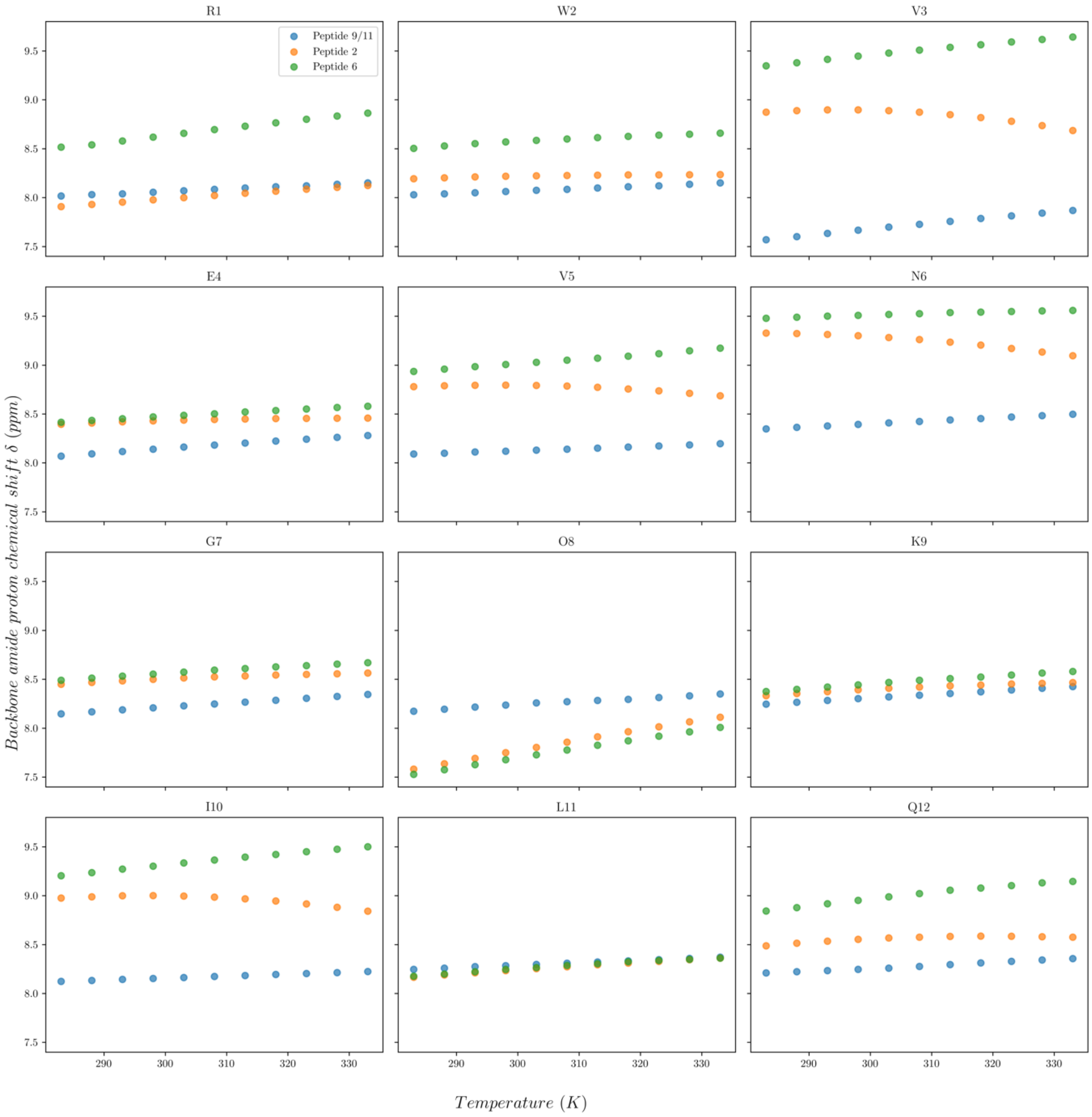

Figure S31. *Backbone amide proton chemical shift values (δ, ppm) of peptide* ***2****,* ***6****,* ***9*** *and* ***11****.*

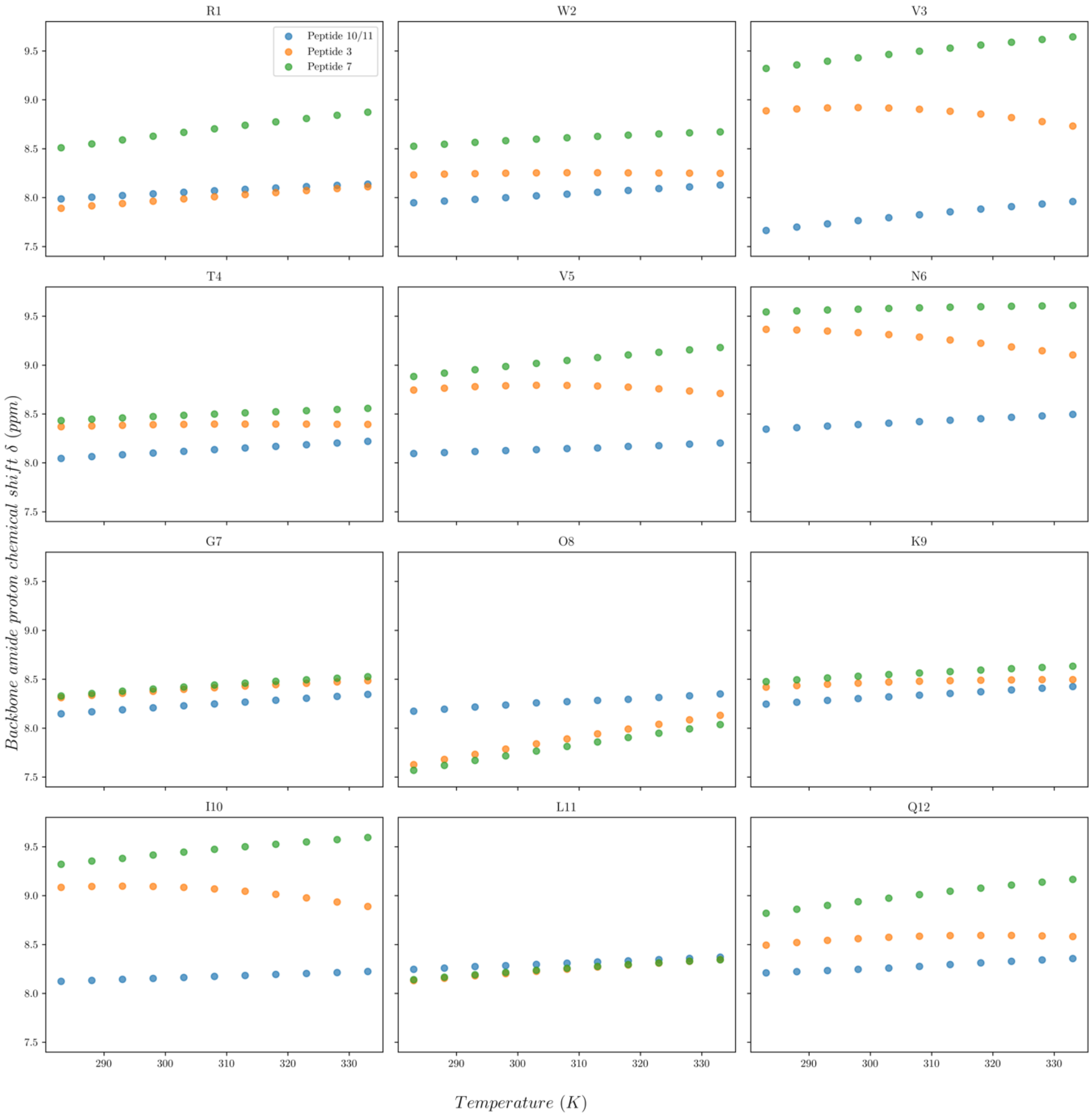

Figure S32. *Backbone amide proton chemical shift values (δ, ppm) of peptide **3**, **7**, **10** and **11**.*

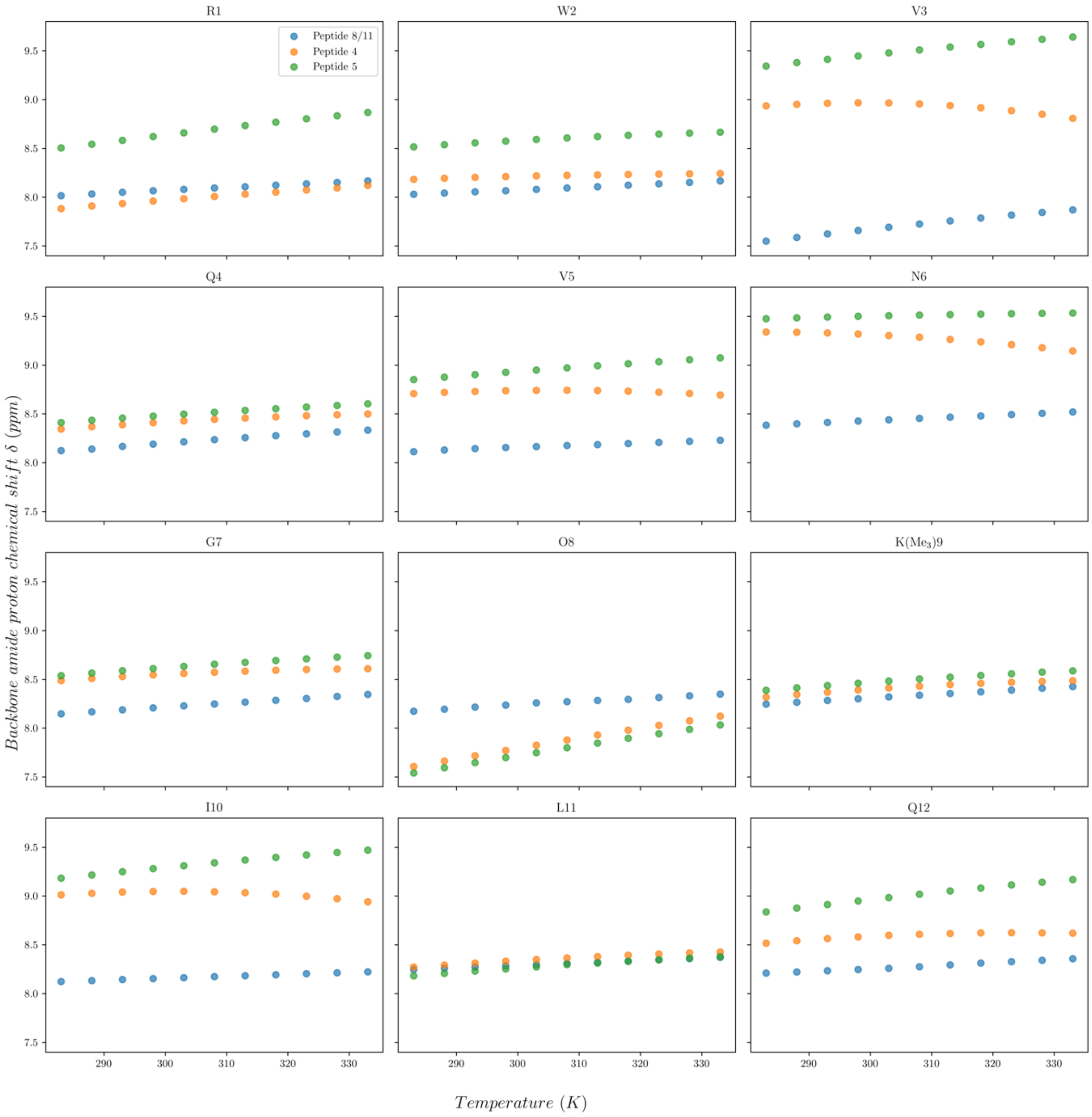

Figure S33. *Backbone amide proton chemical shift values (δ, ppm) of peptide **4**, **5**, **8** and **11**.*

4.3.2. Methodology

Given a chemical shift value or glycine splitting δ_{obs}, the fraction folded can be calculated from equation **(1)**: $f = [\delta_{obs} - \delta_0]/ [\delta_{100} - \delta_0]$, where δ_{100} and δ_0 are respectively chemical shift values of the same residue/species when the peptide is 100% and 0% folded. Subsequently, $\Delta G_u(T)$ can be calculated using equation **(2)**, the data from which will be fitting to the Gibbs-Helmholtz equation **(3)** to obtain $\Delta H^{\circ}_{u,298K}$, $\Delta S^{\circ}_{u,298K}$, $\Delta C^{\circ}_{p,298K}$, and T_{max} (equation **(4)**). The analysis workflow is summarized in Figure S34. As discussed in the previous section, there are two residues where this fitting scheme does not work: R1 (undefined $\Delta G_u(T)$) and L11 (large error and undefined $\Delta G_u(T)$). An interactive "widget" where user can see the shape of $\Delta G_u(T)$ when changing $\Delta H^{\circ}_{u,298K}$,

$\Delta S^{\circ}_{u,298K}$, $\Delta C^{\circ}_{p,298K}$ is provided with the Github link, in two formats: Jupyter notebook (with *ipywidgets*, *numpy*, and *matplotlib* installed) and HTML.

As described in subsection 4.1 above, intrinsic error of $\Delta G_u(T)$ is primarily caused by error propagation following the sequence: chemical shift values δ → fraction folded f → $\Delta G_u(T)$, and the significance of this error depends on the magnitude of fraction folded f (Figure S27, see more in Appendix I). In summary, during data fitting procedures, data points with f exceeding 0.90 (corresponding to a change in Gibbs free energy, ΔG, greater than 5444 J/mol) or falling below 0.10 (ΔG less than -5444 J/mol), where errors exceed 33%, are disregarded.

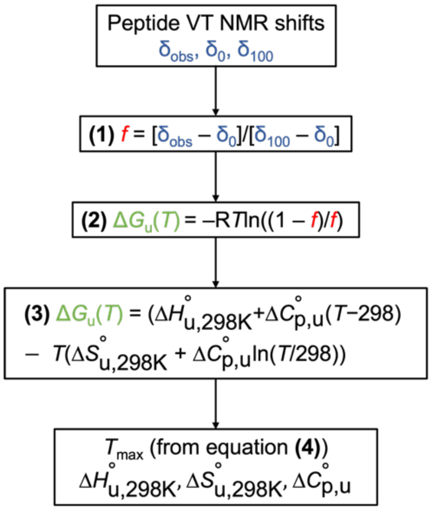

Figure S34. *Data analysis workflow in this study, as applied to both glycine splitting and residue-wise analysis.*

4.3.3. Residue-wise thermodynamics data

In this section, we show the visualization of $\Delta G_u(T)$, the fitting, and the corresponding errors. Fitting of **1-4** (Figure S35-S38) to the equation **(3)** are shown below. Overall, data for **1** incurred bad fitting at K9. Details of which data points were omitted when fitting are provided in the figure descriptions (see below).

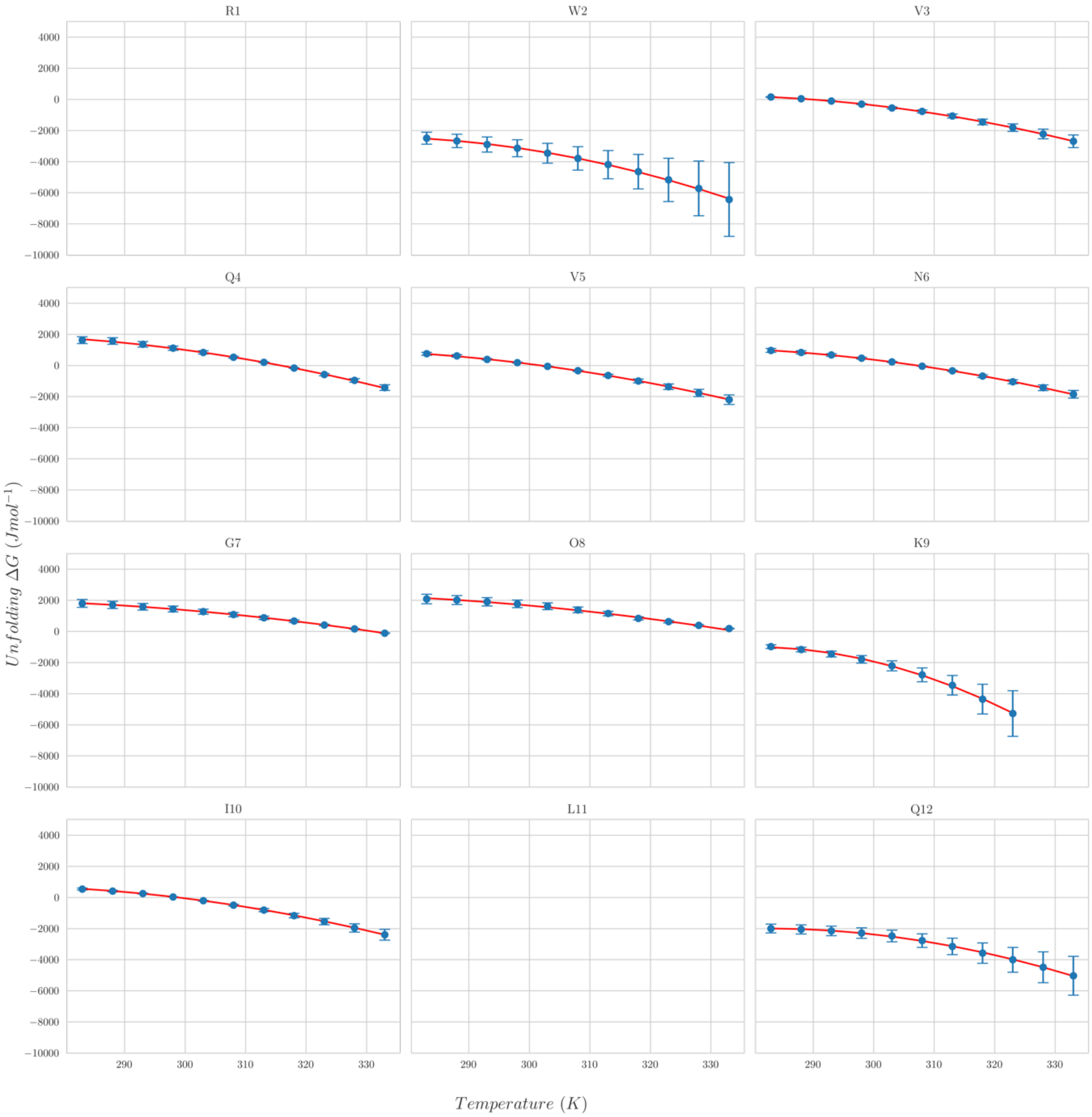

Figure S35. *Fitting (red curves) of $\Delta G_u(T)$ of **1** to equation **(3)** by each residue. The error bars are obtained from error propagation and described in Section 4.1 above. $\Delta G_u(T)$ of R1 and L11 were outside the range and not fitted. The last 2 points of K9 was ignored when fitting due to large errors ($\Delta G_u(T)$ < -5444 J/mol).*

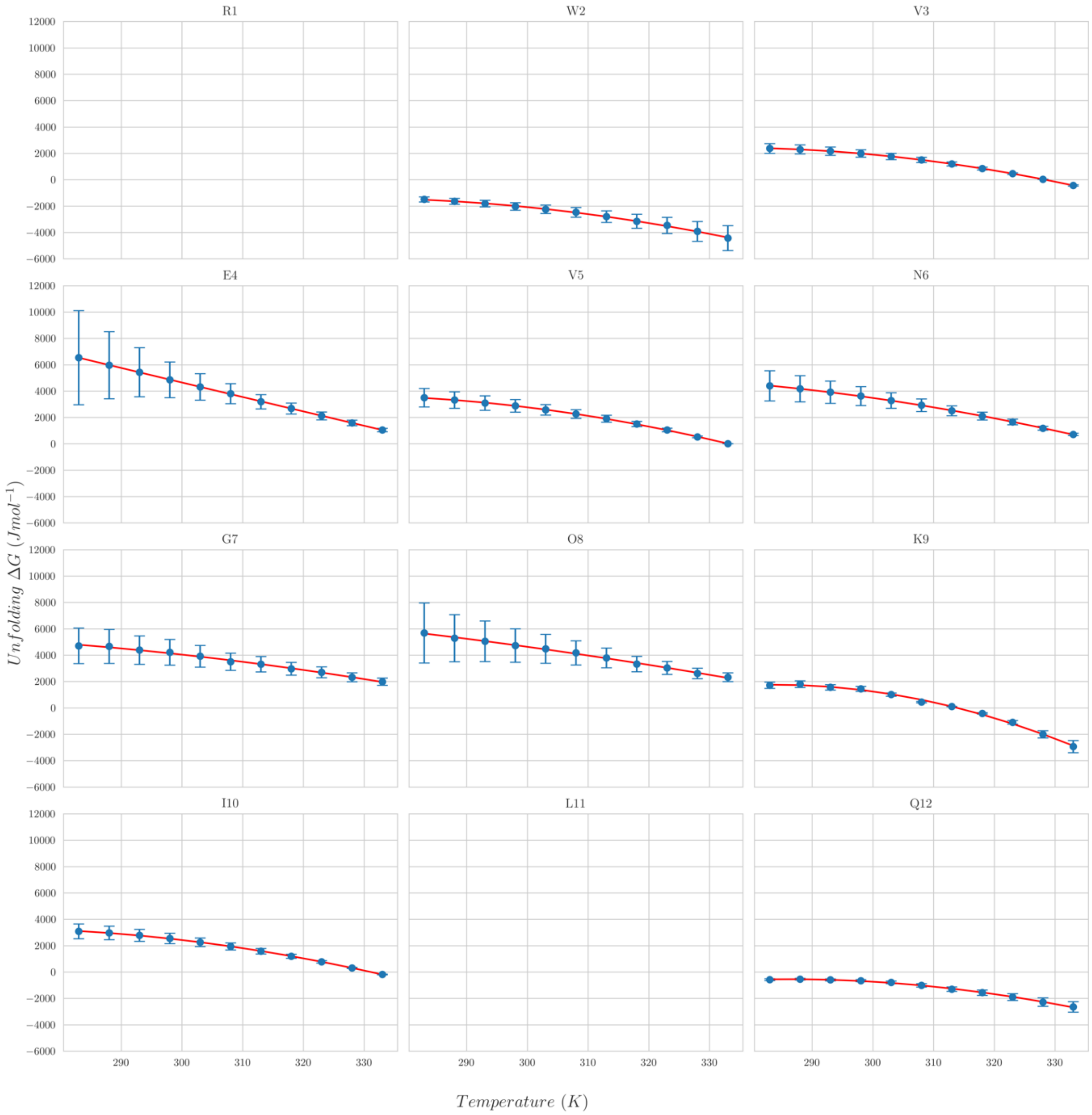

Figure S36. *Fitting (red curves) of $\Delta G_u(T)$ of* ***2*** *to equation* ***(3)*** *by each residue. The error bars are obtained from error propagation and described in Section 4.1 above. $\Delta G_u(T)$ of R1 and L11 were outside the range and not fitted.*

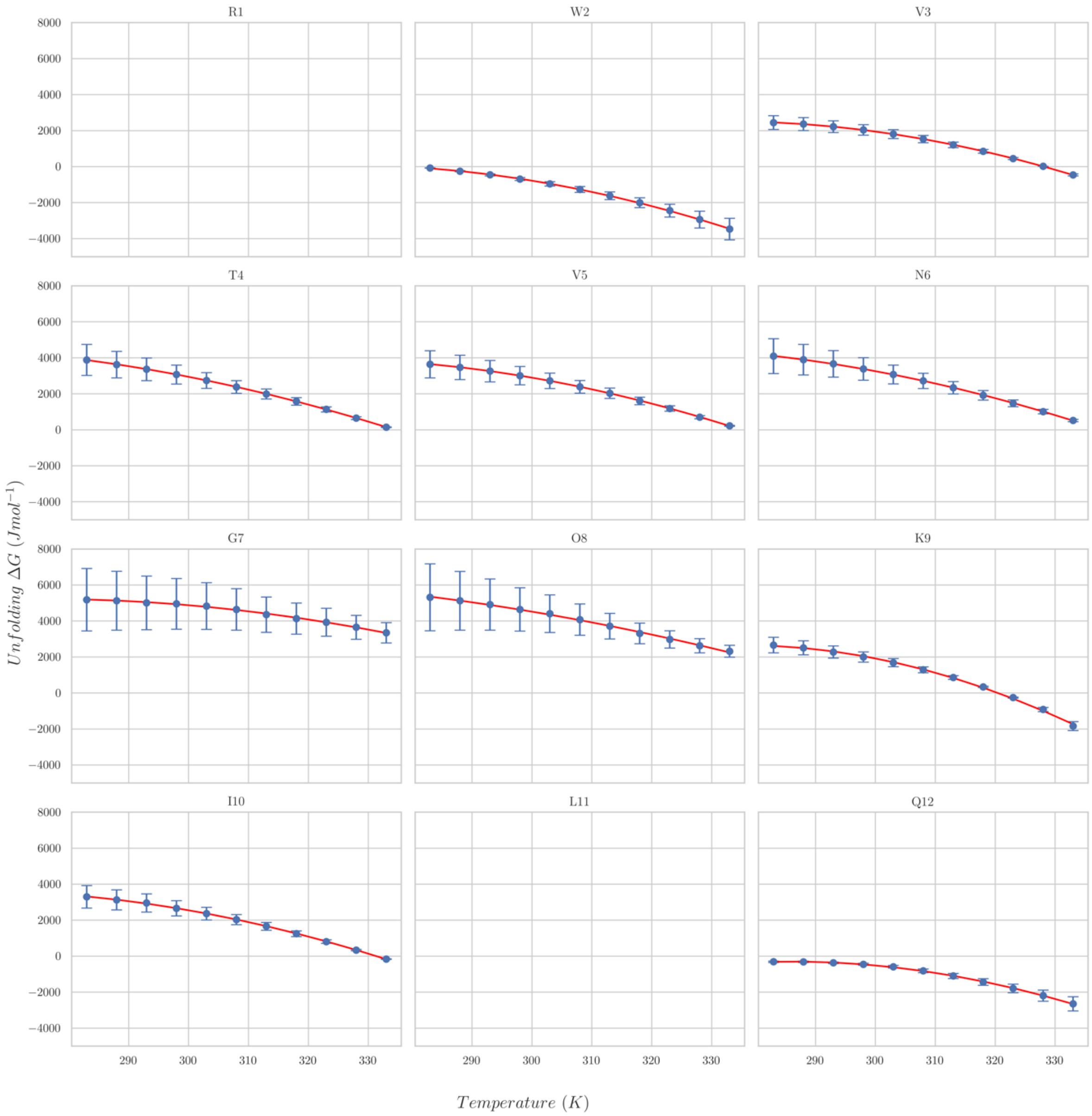

Figure S37. *Fitting (red curves) of $\Delta G_u(T)$ of* ***3*** *to equation* ***(3)*** *by each residue. The error bars are obtained from error propagation and described in Section 4.1 above. $\Delta G_u(T)$ of R1 and L11 were outside the range and not fitted.*

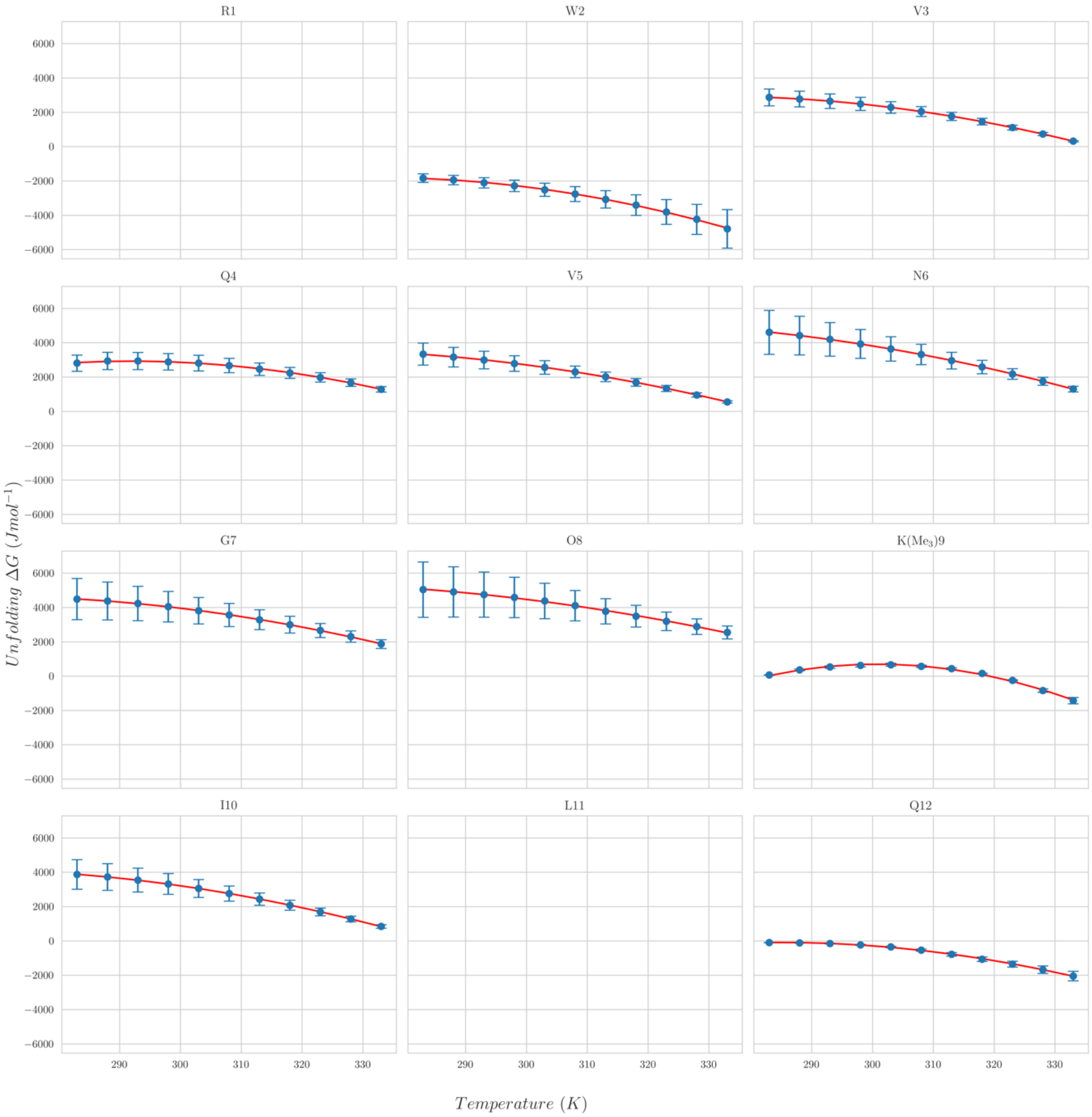

Figure S38. *Fitting (red curves) of $\Delta G_u(T)$ of **4** to equation **(3)** by each residue. The error bars are obtained from error propagation and described in Section 4.1 above. $\Delta G_u(T)$ of R1 and L11 were outside the range and not fitted.*

4.4. Comparison between local fitting and global fitting

To justify for the results of residue-wise fitting, a comparison is made with the data obtained from section 4.2 (Table S33). The average column is calculated based on the results of 10 residues that are fittable (i.e. excluding R1 and L11). Average T_{max} is deducted from the new average $\Delta H°$ and $\Delta S°$. Since those values are merely an average of all residue, no errors are reported.

Table S33. Comparison of unfolding thermodynamics parameters of global fitting (G7 splitting) and by taking average of individual fitting (Average) of 4 peptides

	1		2		3		4	
	Average	G7 splitting[d]	Average	G7 splitting[d]	Average	G7 splitting[d]	Average	G7 splitting[d]
$\Delta G°$ (kJ mol^{-1})[a]	-0.25	0.20 (0.02)	2.37	3.04 (0.52)	2.46	3.28 (0.36)	2.22	3.14 (0.41)
$\Delta H°$ (kJ mol^{-1})[b]	13.76	12.76 (0.51)	18.76	12.95 (1.83)	17.06	20.88 (0.29)	12.05	13.64 (1.78)
$\Delta S°$ (J mol^{-1} K^{-1})[b]	47.00	42.47 (1.73)	55.05	33.03 (6.16)	49.00	59.55 (0.97)	32.96	35.03 (6.01)
$\Delta C_p°$ (J mol^{-1} K^{-1})[b]	530.19	554.3 (43.8)	443.5	677.4 (156.4)	480.6	414.8 (24.6)	512.1	543.6 (27.1)
T_{max} (K)[c]	273	276 (2)	263	284 (4)	269	258 (2)	279	279 (3)

[a] $\Delta G°$ values of G7 splitting are from experiments, not from indirect deduction ($\Delta G° = \Delta H° - T\Delta S°$)
[b] Fitting coefficients from Equation **(3)**
[c] Derived from Equation **(4)**
[d] Errors are reported in brackets

5. Data visualizations

This section summarizes the data obtained from Section 4.

5.1. Unfolding of 1-4

Unfolding heat capacity $\Delta C^{\circ}_{p,u}$, unfolding enthalpy $\Delta H^{\circ}_{u,298K}$, unfolding entropy $\Delta S^{\circ}_{u,298K}$ and maximum temperature of stability T_{max} of **1-4** are visualized respectively in Figure S39-S41 and Table S34.

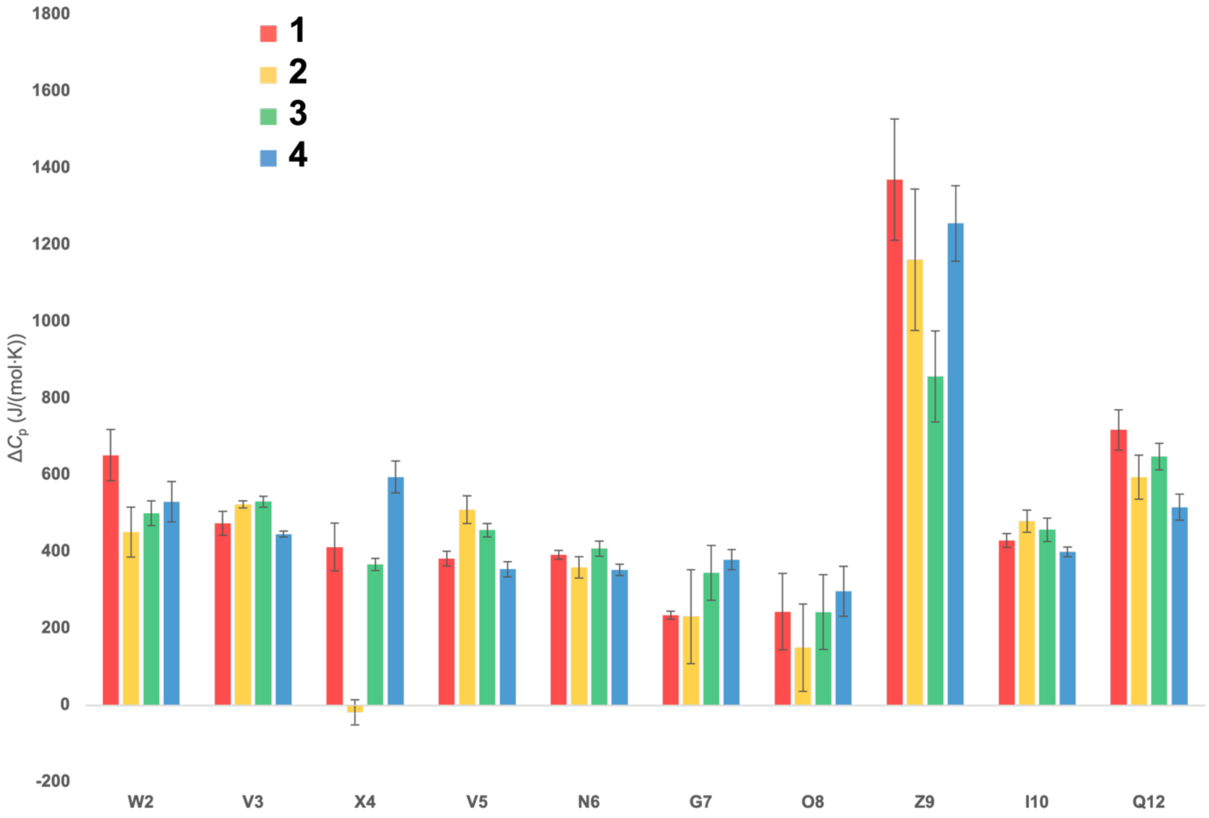

Figure S39. *Heat capacity of unfolding $\Delta C^{\circ}_{p,u}$ (J/(mol·K)) of 4 peptides by residue (**1**: **X** = Q, **Z** = K; **2**: **X** = E, **Z** = K; **3**: **X** = T, **Z** = K; **4**: **X** = Q, **Z** = KMe_3). The data for R1 and L11 were not obtained.*

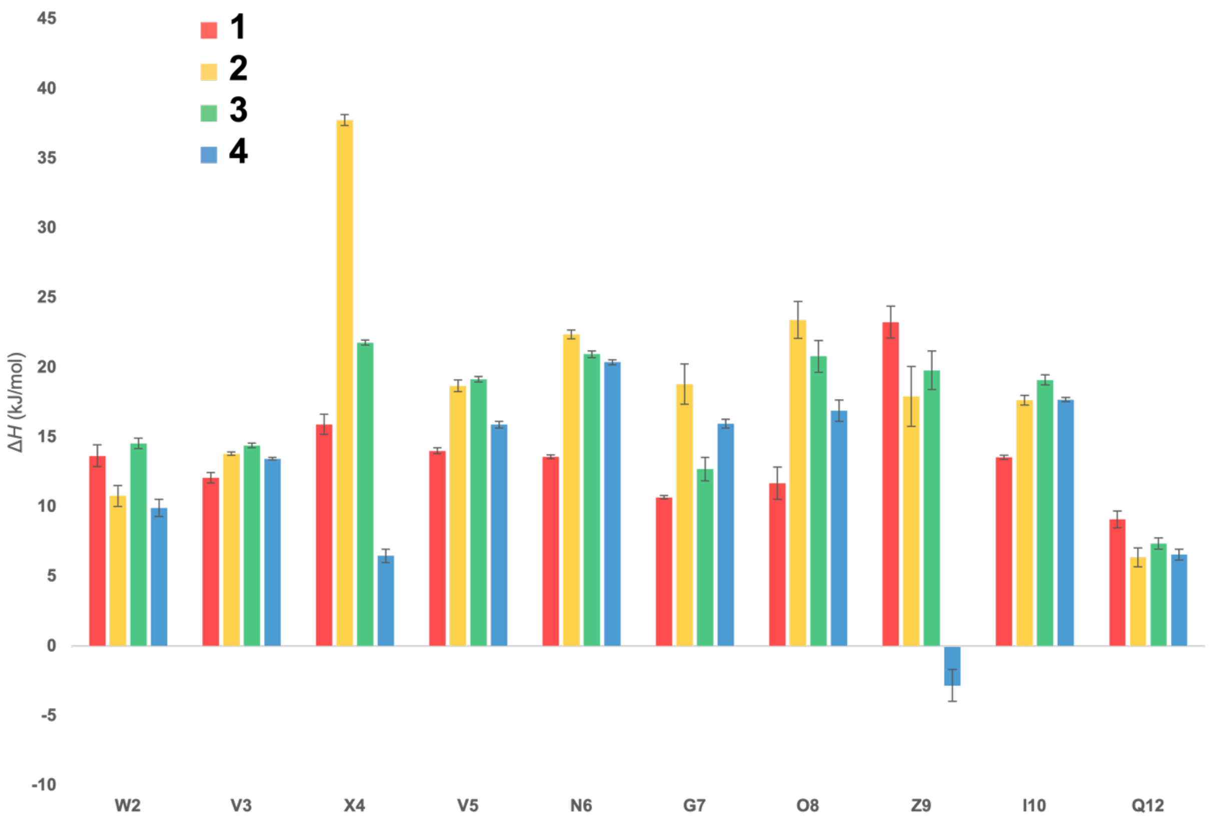

Figure S40. *Enthalpy of unfolding* $\Delta H^{\circ}_{u,298\,K}$ *(kJ/mol) of 4 peptides by residue (**1**: **X** = Q, **Z** = K; **2**: **X** = E, **Z** = K; **3**: **X** = T, **Z** = K; **4**: **X** = Q, **Z** = KMe*$_3$*) at 298 K. The data for R1 and L11 were not obtained.*

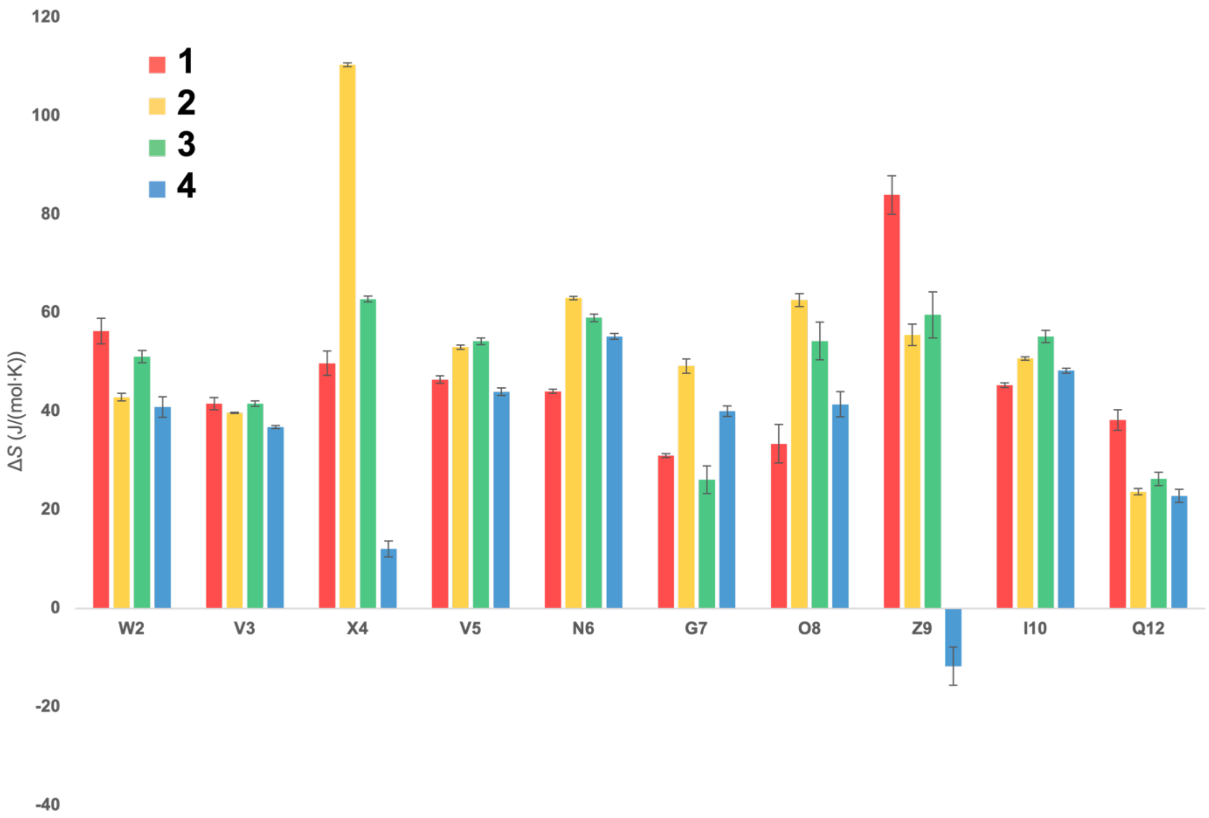

Figure S41. *Entropy of unfolding* $\Delta S^{\circ}_{u,298\,K}$ *(kJ/mol) of 4 peptides by residue (**1**: **X** = Q, **Z** = K; **2**: **X** = E, **Z** = K; **3**: **X** = T, **Z** = K; **4**: **X** = Q, **Z** = KMe*$_3$*) at 298 K. The data for R1 and L11 were not obtained.*

Table S34. *Maximum stability temperature* T_{max} *(K) of 4 peptides by residue (**1**: **X** = Q, **Z** = K; **2**: **X** = E, **Z** = K; **3**: **X** = T, **Z** = K; **4**: **X** = Q, **Z** = KMe*$_3$*). The data for R1 and L11 were not obtained.*

Peptide	W2	V3	X4	V5	N6	G7	O8	Z9	I10	Q12
**1**	273(3)	273(2)	265(5)	264(2)	266(1)	261(2)	260(15)	280(2)	268(1)	283(1)
**2**	271(2)	276(1)	- †	269(2)	250(2)	241(7)	196(10)	284(2)	268(1)	286(2)
**3**	269(2)	276(1)	251(2)	265(1)	258(2)	276(5)	238(22)	278(3)	264(2)	286(1)
**4**	276(2)	274(0)	292(2)	263(1)	255(1)	268(1)	259(3)	301(4)	264(1)	285(1)

† No T_{max} since $\Delta C^{\circ}_{p,u} < 0$.

5.2. Enthalpy-entropy compensation of 1-4

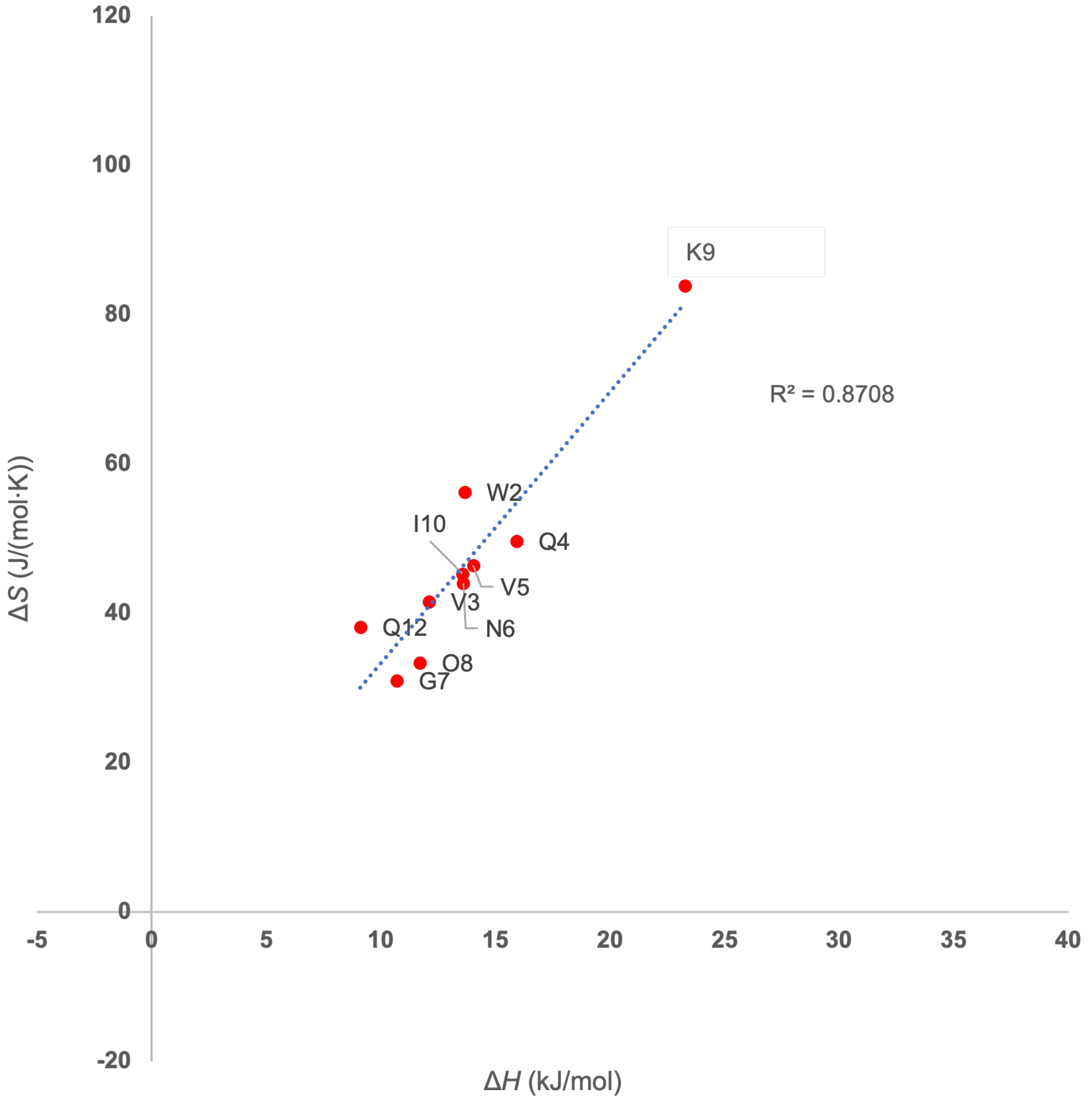

Figure S42. *Enthalpy-entropy compensation of **1**. Coefficient of determination R^2 incurred by linear regression is shown in the plot. Error bars of each data point are omitted for clarity.*

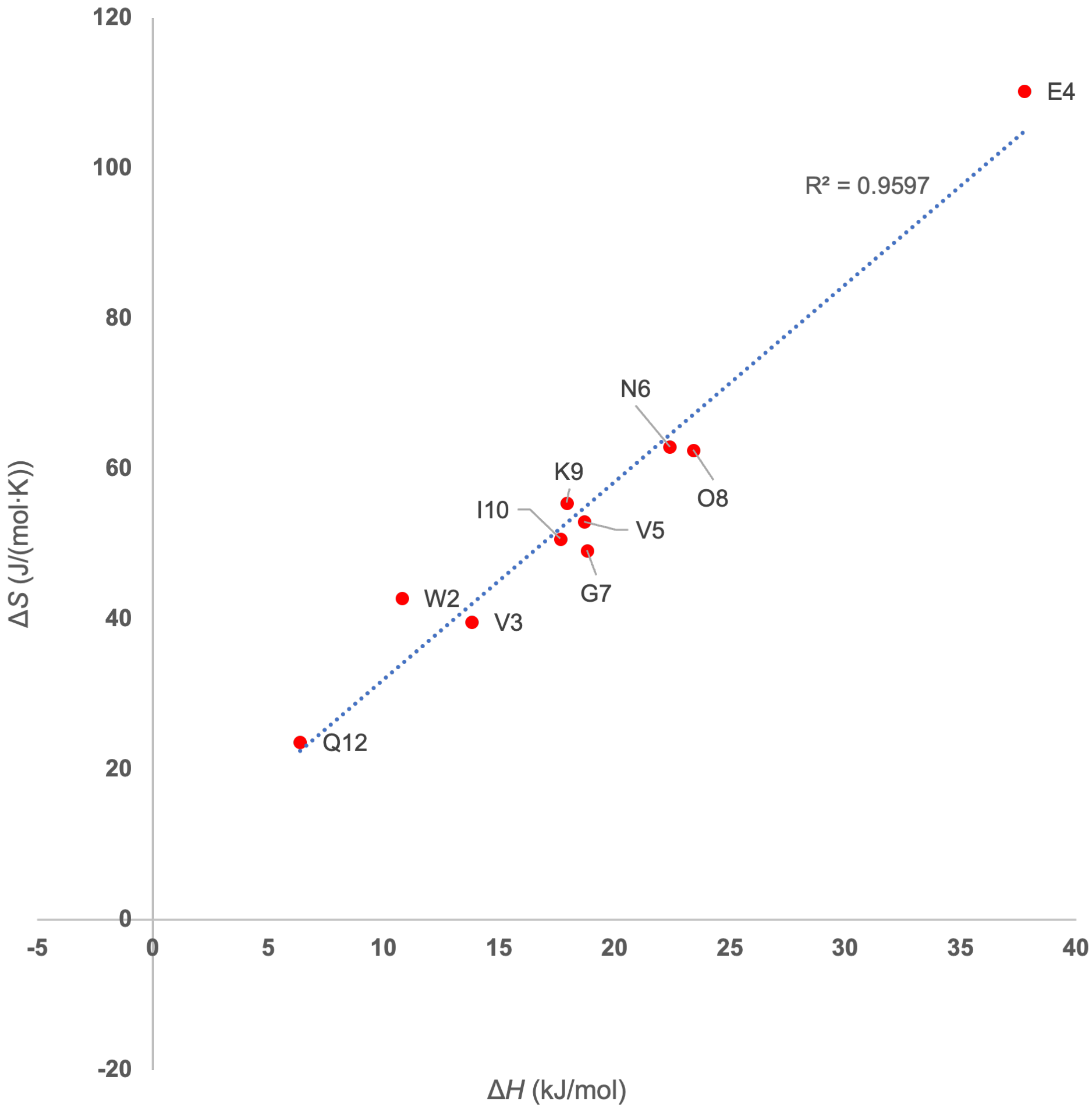

Figure S43. *Enthalpy-entropy compensation of **2**. Coefficient of determination R^2 incurred by linear regression is shown in the plot. Error bars of each data point are omitted for clarity.*

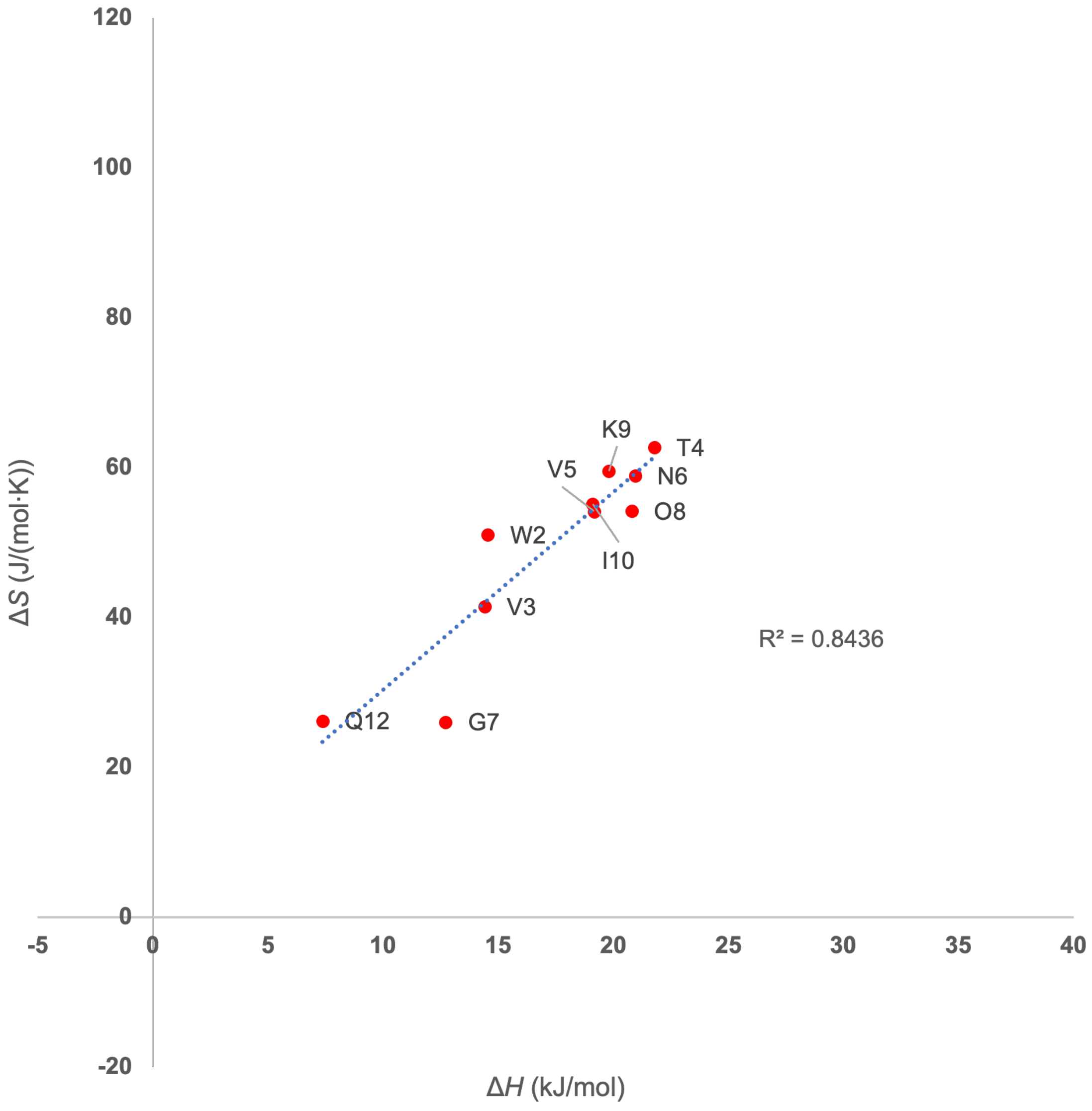

Figure S44. *Enthalpy-entropy compensation of* ***3****. Coefficient of determination* R^2 *incurred by linear regression is shown in the plot. Error bars of each data point are omitted for clarity.*

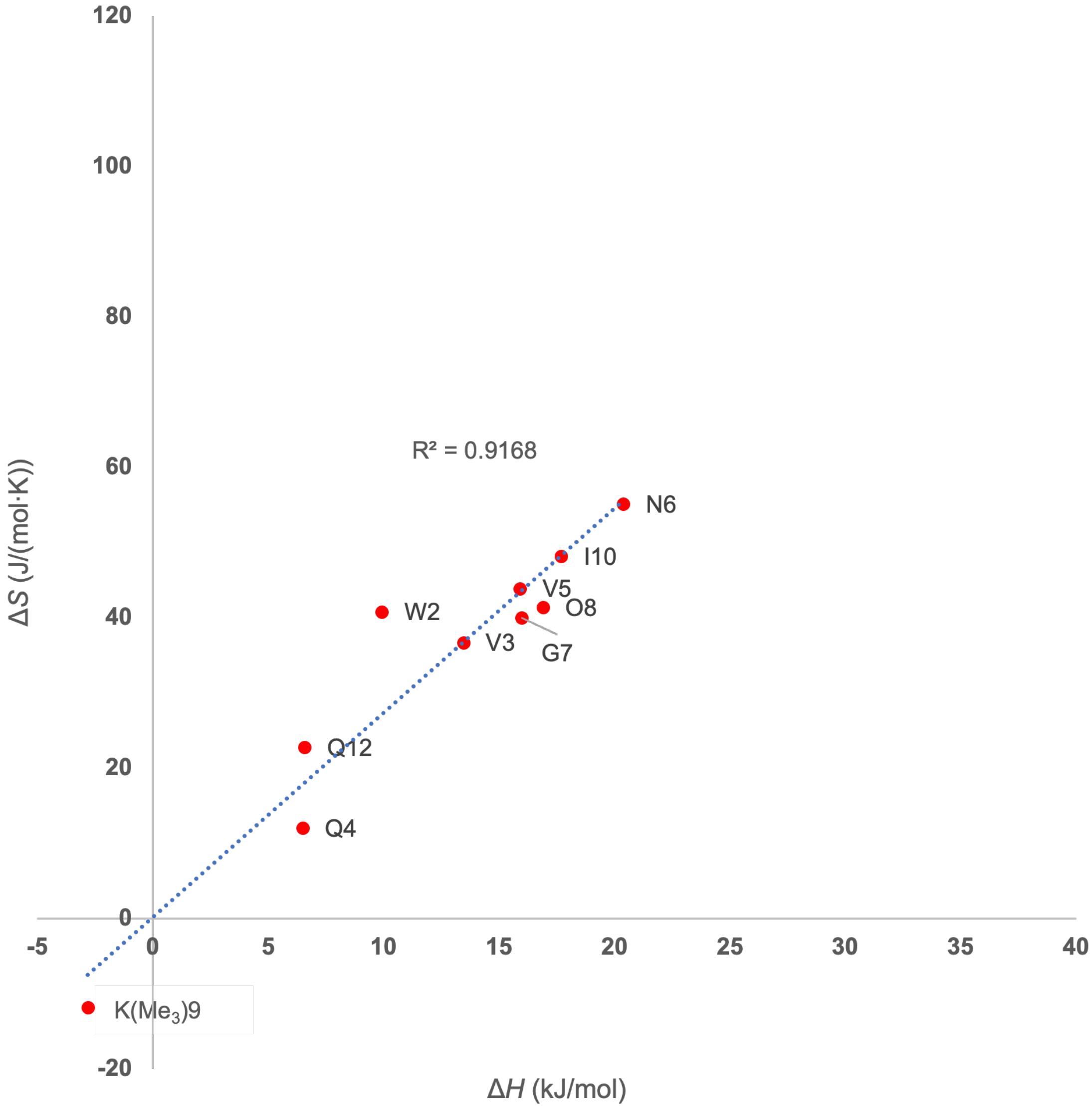

Figure S45. *Enthalpy-entropy compensation of **4**. Coefficient of determination R^2 incurred by linear regression is shown in the plot. Error bars of each data point are omitted for clarity.*

5.3. Enthalpy-entropy compensation of single mutations

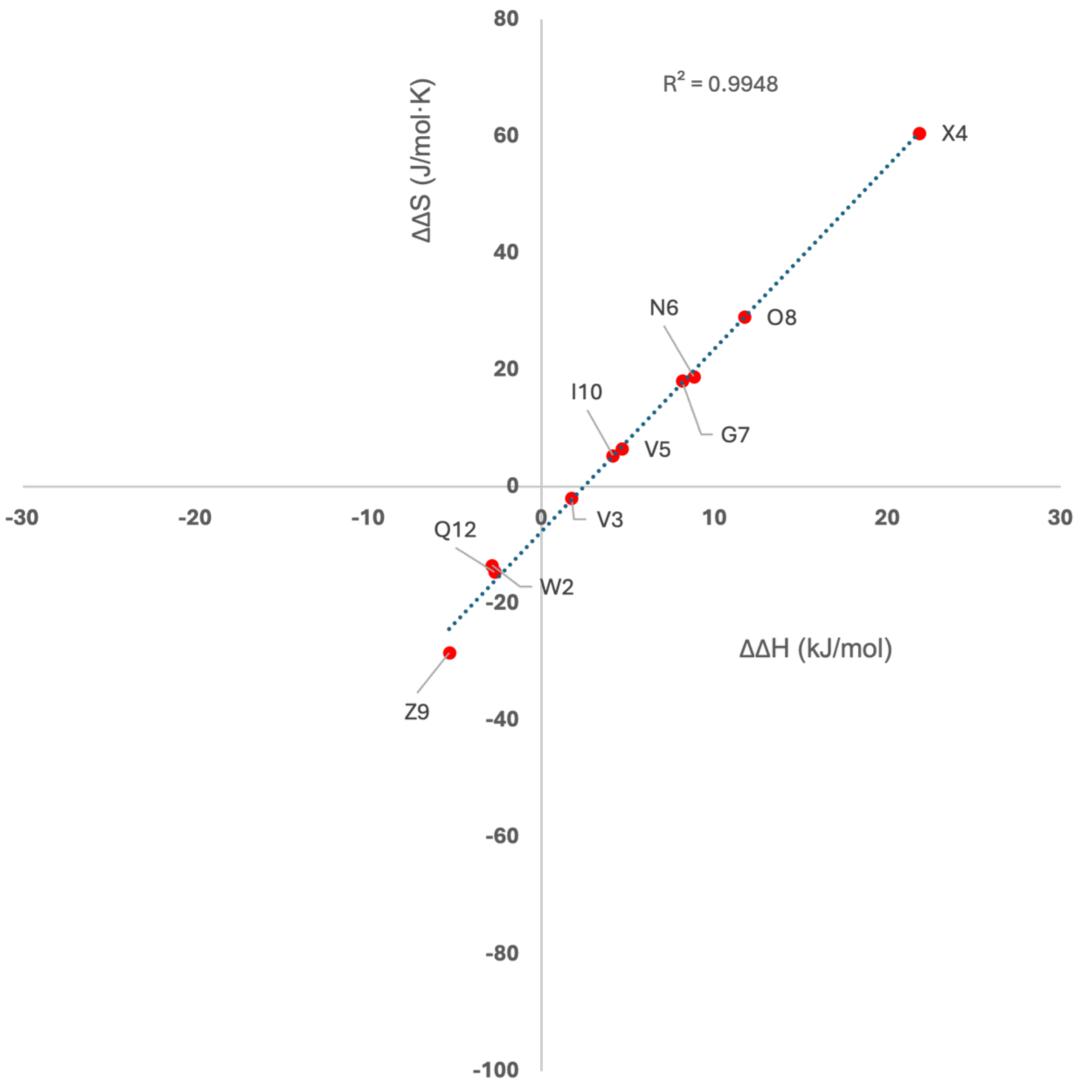

Figure S46. *Enthalpy-entropy compensation of* ***Q4E*** *mutation. Coefficient of determination* R^2 *incurred by linear regression is shown in the plot. Error bars of each data point are omitted for clarity.*

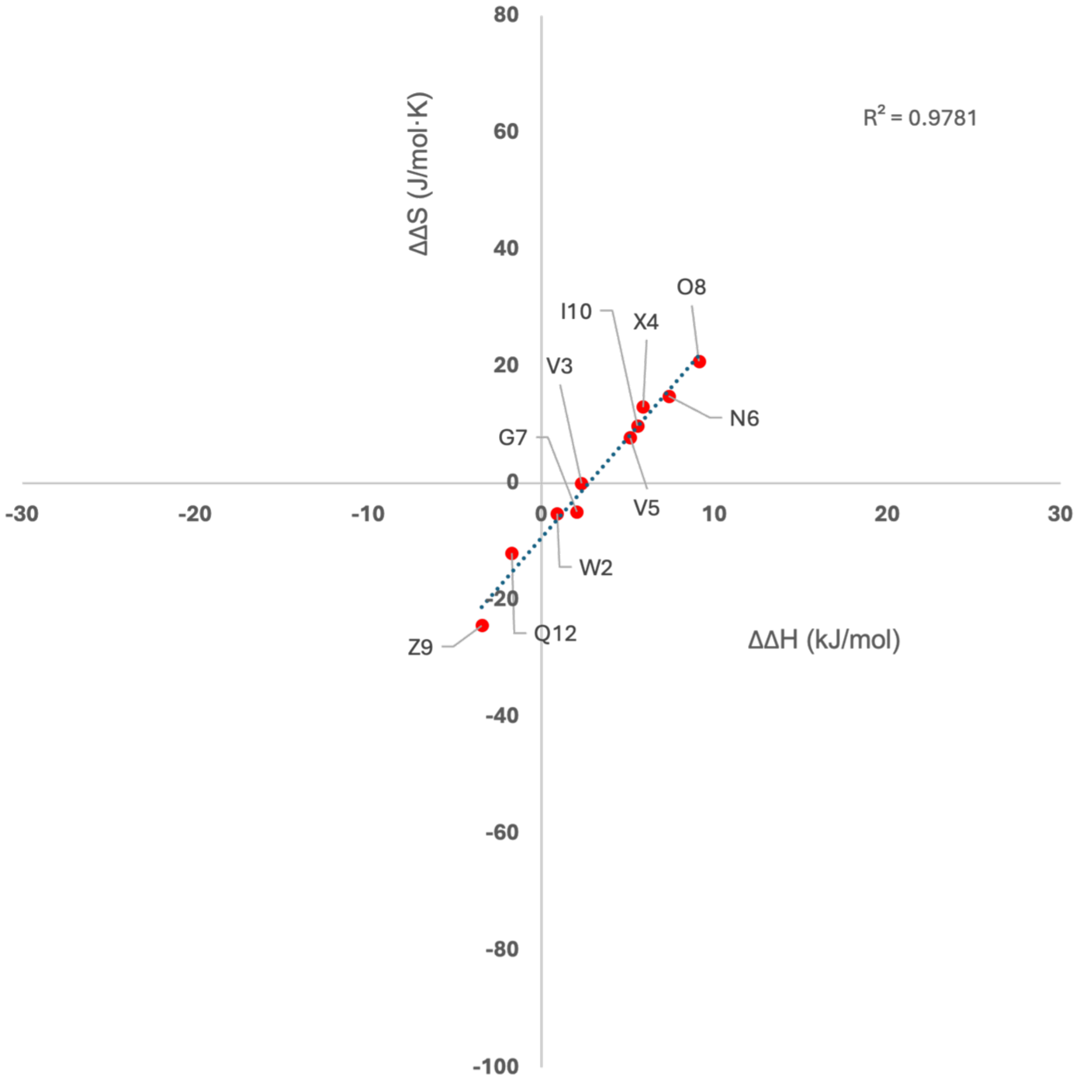

Figure S47. *Enthalpy-entropy compensation of* ***Q4T*** *mutation. Coefficient of determination* R^2 *incurred by linear regression is shown in the plot. Error bars of each data point are omitted for clarity.*

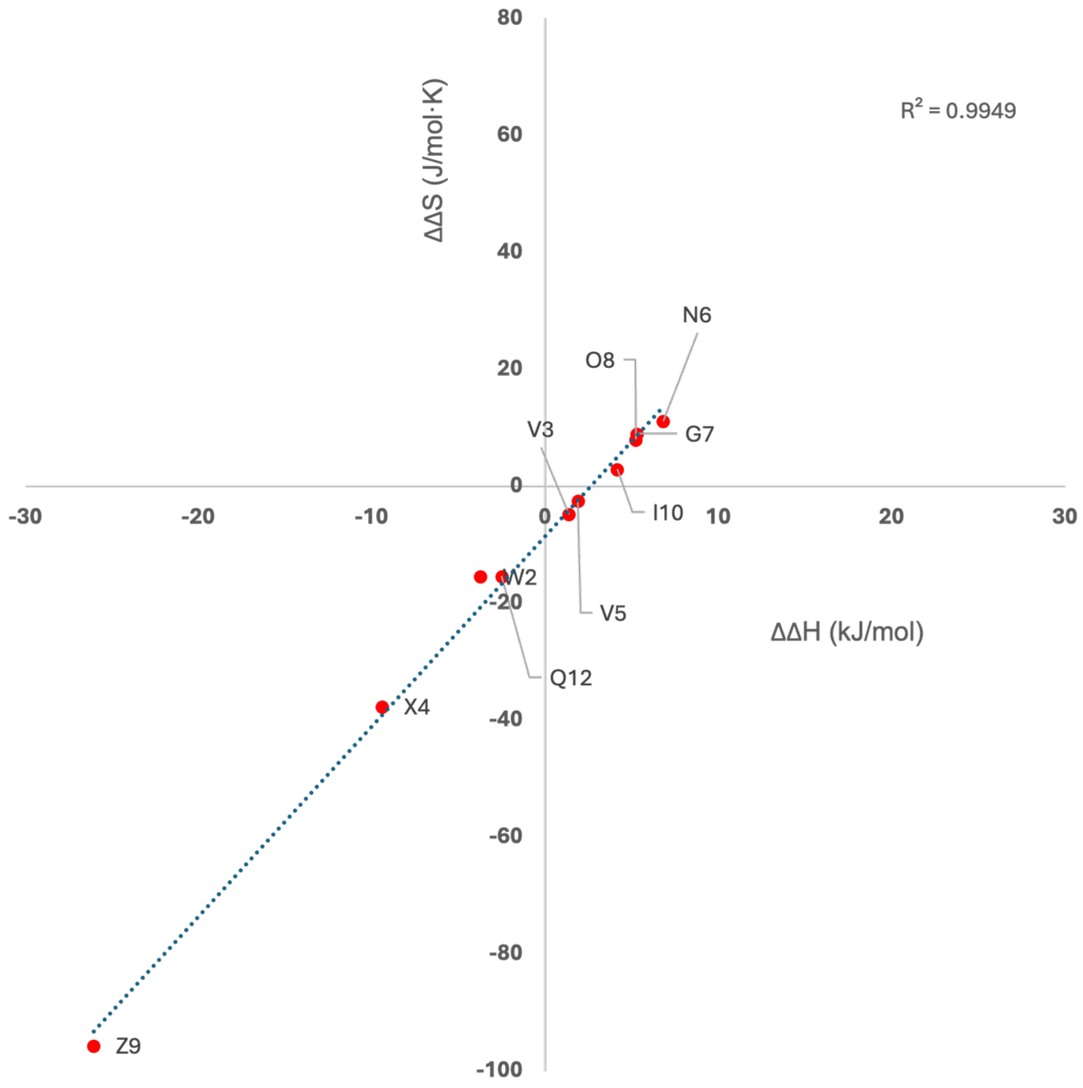

Figure S48. *Enthalpy-entropy compensation of* ***K9KMe₃*** *mutation. Coefficient of determination* R^2 *incurred by linear regression is shown in the plot. Error bars of each data point are omitted for clarity.*

6. Side chain non-monotonicity

As the Δ*G*(T) curves shown above (Figure S35-S38) demonstrate most have their maxima (T_{max}) < 10 °C, which is the lowest temperature investigated here. One could be able to go lower to see the 'real' T_{max}, however, the behavior of water and solutes would become more unpredictable due to several factors (expanding of water density below 4 °C, phase transitions, etc.). The most conspicuous T_{max} values that can be observed in the temperature range of our study are from Q4 and K(Me$_3$)9 of **4** (T_{max} = 19 °C and 28 °C, respectively). Owing to these high T_{max} values, we were able to look at their side chain aliphatic proton, and also observed non-mono-tonicities (Figure S49-S50).

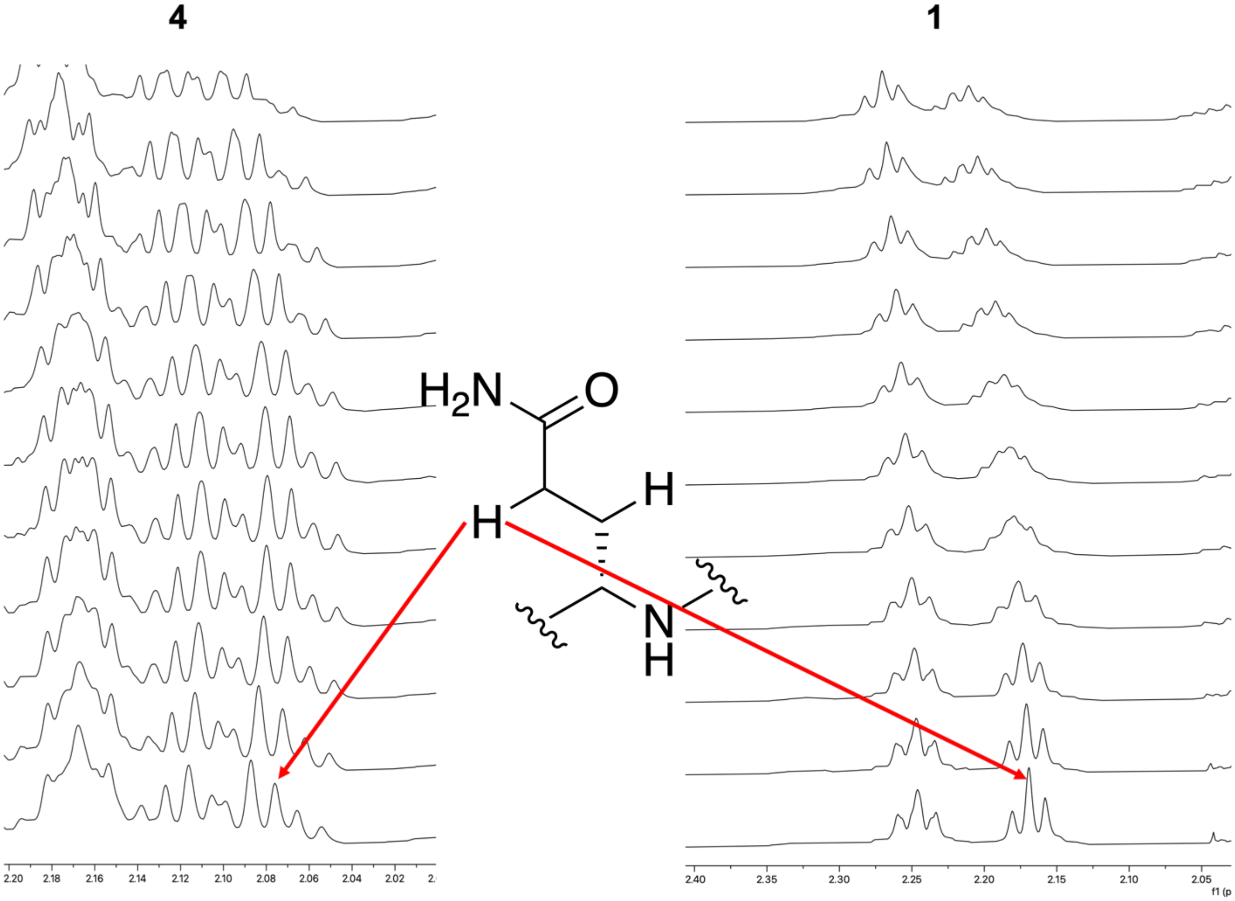

Figure S49. *Representative VT NMR data of side chain non-monotonicity. H_γ of Q4 in **4** (left, δ at 298K = 2.07 ppm) show non-monotonicity, whilst H_γ of Q4 in **1** (right, δ at 298K = 2.17 ppm) does not.*

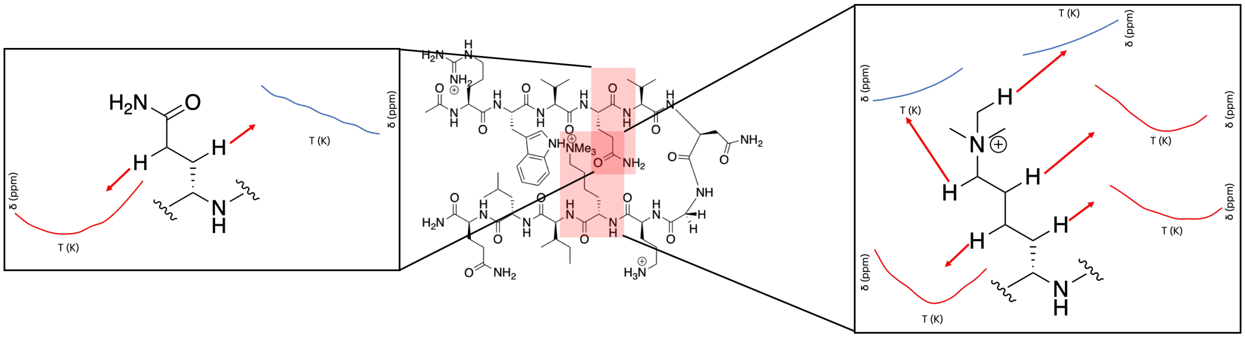

Figure S50. *δ(T) of all aliphatic protons of Q4 (left) and $K(Me_3)9$ (right) in 4. Except H_β of Q4, H_ε and H_η of $K(Me_3)9$, the rest all show curvature, suggesting observable cold denaturation.*

References

1. Hughes, R. M.; Benshoff, M. L.; Waters, M. L., Effects of chain length and N-methylation on a cation-pi interaction in a beta-hairpin peptide. *Chemistry* **2007,** *13* (20), 5753-64.
2. Albanese, K. I.; Waters, M. L., Contributions of methionine to recognition of trimethyllysine in aromatic cage of PHD domains: implications of polarizability, hydrophobicity, and charge on binding. *Chemical Science* **2021,** *12* (25), 8900-8908.
3. Albanese, K. I.; Leaver-Fay, A.; Treacy, J. W.; Park, R.; Houk, K. N.; Kuhlman, B.; Waters, M. L., Comparative Analysis of Sulfonium−π, Ammonium−π, and Sulfur−π Interactions and Relevance to SAM-Dependent Methyltransferases. *Journal of the American Chemical Society* **2022,** *144* (6), 2535-2545.
4. Jumper, J.; Evans, R.; Pritzel, A.; Green, T.; Figurnov, M.; Ronneberger, O.; Tunyasuvunakool, K.; Bates, R.; Žídek, A.; Potapenko, A.; Bridgland, A.; Meyer, C.; Kohl, S. A. A.; Ballard, A. J.; Cowie, A.; Romera-Paredes, B.; Nikolov, S.; Jain, R.; Adler, J.; Back, T.; Petersen, S.; Reiman, D.; Clancy, E.; Zielinski, M.; Steinegger, M.; Pacholska, M.; Berghammer, T.; Bodenstein, S.; Silver, D.; Vinyals, O.; Senior, A. W.; Kavukcuoglu, K.; Kohli, P.; Hassabis, D., Highly accurate protein structure prediction with AlphaFold. *Nature* **2021,** *596* (7873), 583-589.
5. Mirdita, M.; Schütze, K.; Moriwaki, Y.; Heo, L.; Ovchinnikov, S.; Steinegger, M., ColabFold: making protein folding accessible to all. *Nat. Methods* **2022,** *19* (6), 679-682.
6. Davis, M. R.; Dougherty, D. A., Cation-π interactions: computational analyses of the aromatic box motif and the fluorination strategy for experimental evaluation. *Phys Chem Chem Phys* **2015,** *17* (43), 29262-29270.
7. Searle, M. S.; Griffiths-Jones, S.; Skinner-Smith, H., Energetics of Weak Interactions in a β-hairpin Peptide: Electrostatic and Hydrophobic Contributions to Stability from Lysine Salt Bridges. *Journal of the American Chemical Society* **1999,** *121*, 11615-11620.

Appendix I

The formula for the propagation of uncertainty for a function y = *f*(a, b, …) of multiple variables is:

$$\delta y=\sqrt{\left(\frac{\partial y}{\partial a}\delta a\right)^2+\left(\frac{\partial y}{\partial b}\delta b\right)^2+...}$$

where δy, δa, δb, … are corresponding errors of y, a, b,… . In all cases, we assume the variables a, b, … are independent (δ(ab) = 0). The principles of the following derived equations are based on this reference.[1]

1. Error propagation of fraction folded *f*

$$f=\frac{\delta_{G7}}{\delta_{max}}$$

$$\sigma_\delta=\sqrt{(0.005\text{ ppm})^2+(0.005\text{ ppm})^2}=0.007\text{ ppm}$$

$$\sigma_f=\sqrt{\left(\frac{0.007}{\delta_{G7}}\right)^2+\left(\frac{0.007}{\delta_{max}}\right)^2}$$

where 0.005 ppm is the error of chemical shift values. Then the error of fraction folded ranges from 2-3% depending on each peptide.

2. Error propagation of Δ*G*(*T*)

Let *f* and δ*f* be respectively the fraction folded and its error, *T* and δ*T* the corresponding temperature and its error, then the uncertainty of Δ*G*(*T*) deriving from Δ*G*(*T*) = –R*T*ln(*f*/(1–*f*)) is determined by:

$$\delta\Delta G(T)=\sqrt{\left(\frac{\partial\Delta G(T)}{\partial T}\delta T\right)^2+\left(\frac{\partial\Delta G(T)}{\partial f}\delta f\right)^2}$$

$$\Leftrightarrow \delta\Delta G(T)=\Delta G(T)*\sqrt{\left(\frac{0.1\text{ K}}{T}\right)^2+\left(\left|\frac{-1}{1-f}-\frac{1}{f}\right|*0.03\right)^2} \qquad \textbf{(i)}$$

$$\Leftrightarrow \frac{\delta\Delta G(T)}{\Delta G(T)}=\sqrt{\left(\frac{0.1\text{ K}}{T}\right)^2+\left(\left|\frac{-1}{1-f}-\frac{1}{f}\right|*0.03\right)^2}\approx\left|\frac{-1}{1-f}-\frac{1}{f}\right|*0.03 \qquad \textbf{(ii)}$$

where 0.1 K is the temperature error, and 0.03 is the maximum error of fraction folded (see Section I.1 above). We can consider equation **(ii)** as a single variable. Hence, the %error of Δ*G*(*T*) is a function of fraction folded *f*, as visualized in Figure S20 in the Supporting Information.

3. Error propagation of T_{max}

From the Gibbs-Helmholtz equation we have:

$$T_{max} = 298 \times e^{-\frac{\Delta S^{\circ}_{u,298K}}{\Delta C^{\circ}_{p,u}}}$$

Let δT_{max} be the error in T_{max}, and $\delta\Delta C^{\circ}_{p,u}$ and $\delta\Delta S^{\circ}_{u,298K}$ be respectively the errors of $\Delta C^{\circ}_{p,u}$ and $\Delta S^{\circ}_{u,298K}$, then the uncertainty of T_{max} is determined by:

$$\delta T_{max} = \sqrt{\left(\frac{\partial T_{max}}{\partial \Delta C^{\circ}_{p,u}} \delta\Delta C^{\circ}_{p,u}\right)^2 + \left(\frac{\partial T_{max}}{\partial \Delta S^{\circ}_{u,298K}} \delta\Delta S^{\circ}_{u,298K}\right)^2}$$

$$\Leftrightarrow \delta T_{max} = \sqrt{\left(\frac{T_{max} * \delta\Delta S^{\circ}_{u,298K}}{\Delta C^{\circ}_{p,u}}\right)^2 + \left(\frac{T_{max} * \Delta S^{\circ}_{u,298K} * \delta\Delta C^{\circ}_{p,u}}{{\Delta C^{\circ}_{p,u}}^2}\right)^2}$$

References

(1) Taylor, J. R.; Thompson, W. An introduction to error analysis: the study of uncertainties in physical measurements; Springer, 1982.
